# Integrating Phenotypic and Chemoproteomic Approaches to Identify Covalent Targets of Dietary Electrophiles in Platelets

**DOI:** 10.1021/acscentsci.3c00822

**Published:** 2024-01-29

**Authors:** Ivy A. Guan, Joanna S. T. Liu, Renata C. Sawyer, Xiang Li, Wanting Jiao, Yannasittha Jiramongkol, Mark D. White, Lejla Hagimola, Freda H. Passam, Denise P. Tran, Xiaoming Liu, Simone M. Schoenwaelder, Shaun P. Jackson, Richard J. Payne, Xuyu Liu

**Affiliations:** † School of Chemistry, Faculty of Science, The University of Sydney, Sydney, New South Wales 2006, Australia; ‡ The Heart Research Institute, The University of Sydney, Newtown, New South Wales 2042, Australia; § School of Medical Sciences, Faculty of Medicine and Health, The University of Sydney, Sydney, New South Wales 2006, Australia; ∥ Department of Medicine, Washington University in St. Louis, St. Louis, Missouri 63110, United States; ⊥ McDonnell Genome Institute, Washington University in St. Louis, St. Louis, Missouri 63108, United States; # Ferrier Research Institute, Victoria University of Wellington, Wellington 6140, New Zealand; ∇ Maurice Wilkins Centre for Molecular Biodiscovery, Auckland 1142, New Zealand; • Sydney Mass Spectrometry, The University of Sydney, Camperdown, New South Wales 2006, Australia; ° Charles Perkins Centre, The University of Sydney, Sydney, New South Wales 2006, Australia; ¶ Australian Research Council Centre of Excellence for Innovations in Peptide and Protein Science, The University of Sydney, Sydney, New South Wales 2006, Australia

## Abstract

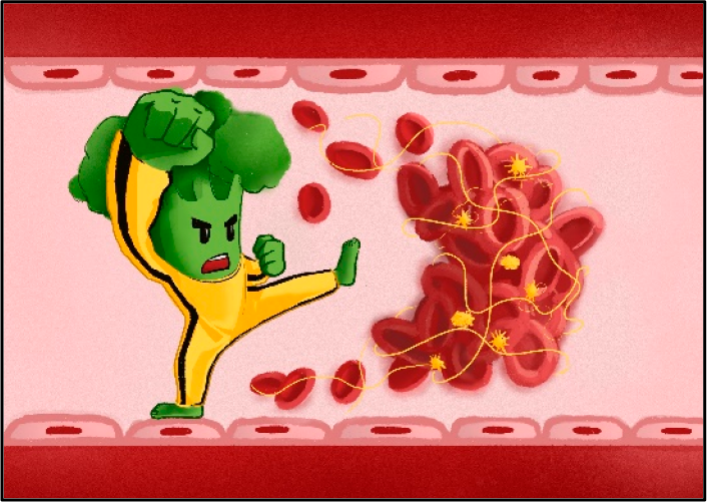

A large variety of dietary phytochemicals has been shown to improve thrombosis and stroke outcomes in preclinical studies. Many of these compounds feature electrophilic functionalities that potentially undergo covalent addition to the sulfhydryl side chain of cysteine residues within proteins. However, the impact of such covalent modifications on the platelet activity and function remains unclear. This study explores the irreversible engagement of 23 electrophilic phytochemicals with platelets, unveiling the unique antiplatelet selectivity of sulforaphane (SFN). SFN impairs platelet responses to adenosine diphosphate (ADP) and a thromboxane A2 receptor agonist while not affecting thrombin and collagen-related peptide activation. It also substantially reduces platelet thrombus formation under arterial flow conditions. Using an alkyne-integrated probe, protein disulfide isomerase A6 (PDIA6) was identified as a rapid kinetic responder to SFN. Mechanistic profiling studies revealed SFN’s nuanced modulation of PDIA6 activity and substrate specificity. In an electrolytic injury model of thrombosis, SFN enhanced the thrombolytic activity of recombinant tissue plasminogen activator (rtPA) without increasing blood loss. Our results serve as a catalyst for further investigations into the preventive and therapeutic mechanisms of dietary antiplatelets, aiming to enhance the clot-busting power of rtPA, currently the only approved therapeutic for stroke recanalization that has significant limitations.

## Abstract 2

Sulforaphane, found in cruciferous vegetables such as broccoli, offers selective, irreversible antiplatelet effects, synergizing with vascular recanalization therapies without increasing bleeding risks.

## Introduction

Platelets are crucial cellular components of the blood that orchestrate hemostasis, preventing blood loss by forming stable clots in response to vascular injury. However, they also play a major role in thrombosis, eliciting an exaggerated thrombotic response when exposed to pathological conditions including atherosclerotic plaque rupture,^1^ disturbed fluid flow,^1^ and elevated blood velocity.^2^ Pathological thrombosis may obstruct blood flow in veins, arteries, or smaller vessels, inhibiting the delivery of essential nutrients, such as glucose and oxygen, to critical organs. Platelet-mediated thrombosis underlies the development of numerous cardiovascular diseases, including ischemic stroke^3^ and myocardial infarction (heart attack).^4^ Collectively, these diseases are the leading cause of death and disability globally, making them a massive burden on healthcare systems and caregivers.^5,6^

Over the past three decades, significant advancements have been made in identifying the fundamental mechanisms of platelet function and activation.^1,7^ These discoveries have advanced antiplatelet therapeutic development. However, currently approved antithrombotic strategies do not effectively discriminate between hemostasis and thrombosis and inhibit critical platelet functions relevant for hemostasis, for example, the inhibition of the major platelet integrin α_IIb_β_3_,^8^ which can cause life-threatening bleeding complications. Therefore, a better understanding of the unique signaling processes that differentiate platelet-mediated thrombosis and hemostasis could facilitate the development of targeted antithrombotic therapies that do not increase the bleeding risk. Antithrombotics, including antiplatelet agents, have been shown to improve reperfusion therapy in acute coronary syndromes and are now incorporated into the standard-of-care regimen as adjuncts for thrombolytic therapy;^9,10^ the efficiency of vessel recanalization/reperfusion is closely linked to improved patient outcomes.^11^ However, all current antiplatelet agents are contraindicated for adjunctive therapies for thrombolysis in stroke patients due to the high risk of symptomatic brain hemorrhage, which is the most feared complication of thrombolytic therapy.^8,12^

A large variety of dietary phytochemicals have been identified as potential thromboprophylaxis that could enhance the outcomes of stroke treatment and management.^13−16^ These compounds also demonstrate favorable tolerability and safety profiles in individuals with thrombotic or bleeding disorders.^15,17−19^ Many dietary phytochemicals possessing α,β-unsaturated carbonyl,^20,21^ isothiocyanate,^22^ and other electrophilic functionalities have been associated with the covalent modulation of proteins influencing transcription factors^23^ and other gene-regulatory machineries.^24^ An archetypal response occurs through covalent inhibition of the E3 ligase Keap1, which in turn liberates the Nrf2 transcription factor to upregulate the expression of antioxidant and detoxification enzymes.^23,25^ Despite these advances, our understanding of their influence on platelet reactivity is still limited, as the conventional model of transcriptional regulation plays a limited role in regulating platelet function due to the absence of a functional genome in platelets.

**Figure 1:**
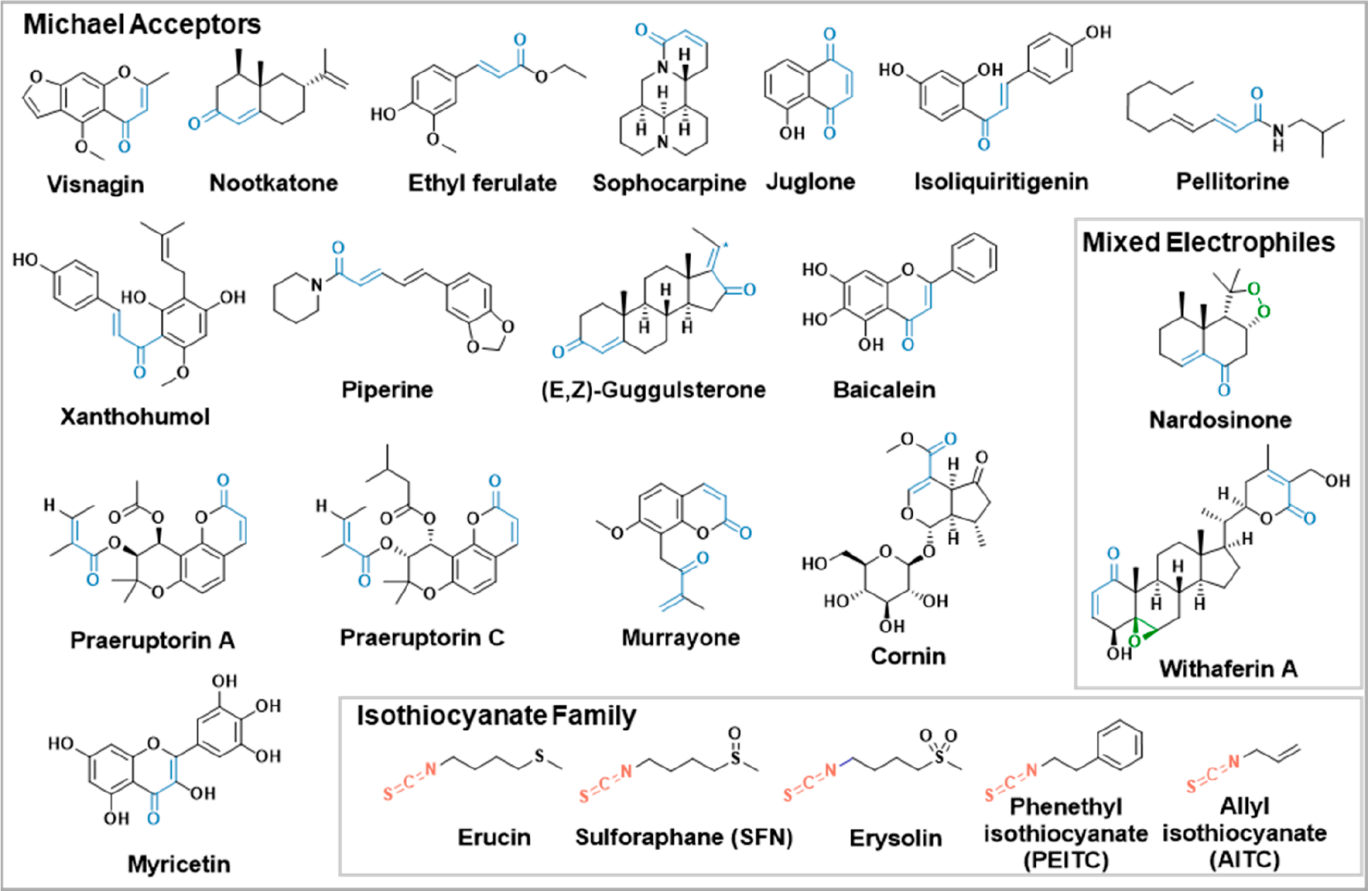
Chemical structures of selected natural products with electrophilic functional groups highlighted in blue (Michael acceptors), green (epoxide and peroxide), and red (isothiocyanate).

In this study, our objective was to characterize the antiplatelet phenotypes associated with the covalent modification of proteins by dietary phytochemicals (Figure 1). As part of this work, we sought to identify the most impacted targets and also highlight the potential long-term consequences related to the dietary consumption of these compounds. Given the limited capacity of platelets to resynthesize proteins,^26,27^ covalent protein modifications are anticipated to have a more profound impact on platelet (patho)physiology compared to other human cell types. In this report, we introduce an integrated phenotypic and chemical proteomics approach to examine the prevalence of covalent modifications induced by plant-derived natural products with α,β-unsaturated, isothiocyanate, and a mixture of electrophilic moieties.

## Results and Discussion

### Streamlined Preparation Protocol for Interrogating Irreversible Platelet Inhibition

Numerous natural products interact with protein targets through a blend of reversible and irreversible binding modes.^28^ In particular, several archetypal phytochemicals found in diets and in herbal medicines, once thought to promote health benefits via reversible binding to proteins, have now been found to operate via covalent modes of inhibition that result in long-lasting changes in protein activity and function.^29−35^ We envisaged that the biological influence of covalent engagement could be effectively assessed via jump-dilution and washout experiments, two methods frequently employed to examine the impact of irreversible inhibitors on cellular activity.^36,37^ We began with integrating a washout procedure into our platelet preparation protocol (Figure S2). This involved pelleting compound- or vehicle-treated platelets and subjecting them to a wash protocol with a standard platelet wash buffer (PWB, see Supporting Information Section 2.1). Subsequently, the platelets were resuspended in physiological Tyrode’s buffer for aggregation assays. Although encouraging antiplatelet effects of several natural products were initially uncovered, a marked decrease in platelet activity, including in the control sample, was observed 1 h postwashout. This phenomenon may be attributed to the increased handling of platelets, which may inadvertently activate the cells and decrease the overall viability of the platelet population. As such, we devised an alternative approach by incorporating the jump-dilution method into our platelet preparation protocol (see Supporting Information Section 2.1). Briefly, freshly isolated platelets were incubated with PWB containing 20 μM natural product for 120 min at 37 °C to facilitate potential covalent engagement. Subsequently, the platelets were pelleted and subjected to a 200-fold dilution in volume with Tyrode’s buffer to achieve a final concentration of 3 × 10^8^ cells/mL. The functional activity of these platelets was examined in a medium-throughput manner by employing light transmission aggregometry.

### Distinct Antiplatelet Activity Profiles Associated with Irreversible Modulation by Dietary Electrophiles

**Figure 2:**
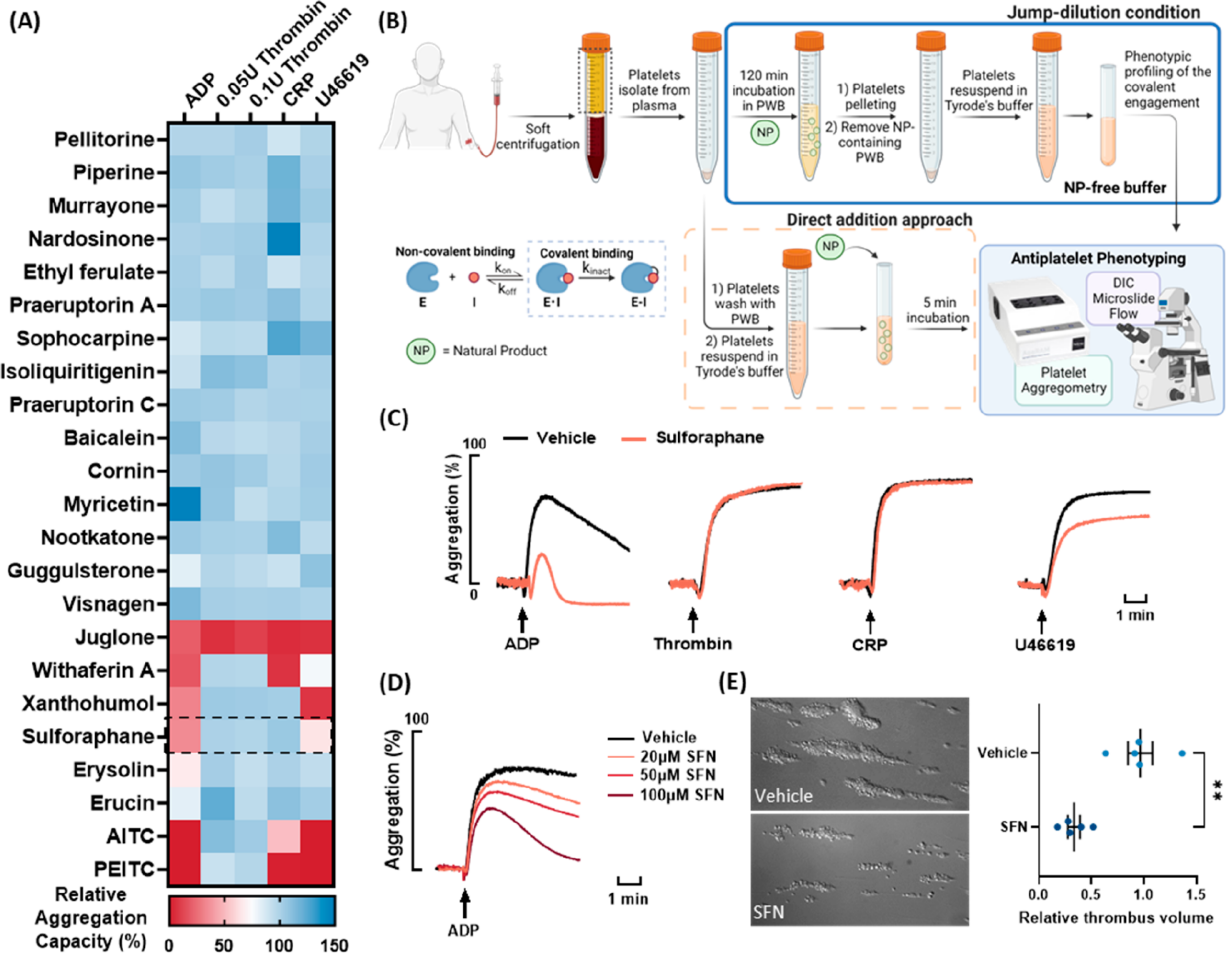
Antiplatelet phenotypic screening of electrophilic phytochemicals. (A) Heat map depicting the effects of natural products (20 μM) on platelet activities in response to ADP (2–5 μM), thrombin (0.05–0.1 U/mL), collagen-related peptide (CRP, 0.5 μg/mL), and U46619 (0.2–0.5 μM) induced aggregation following a jump-dilution preparation procedure. The relative aggregation capacity (%) was calculated according to Figure S3. (B) Schematic illustration of the platelet washing and natural product incubation workflow: the “jump-dilution” approach and the conventional “direct addition” approach employed to examine the antiplatelet activities. (C) Representative light-transmission platelet aggregometry profiles: the aggregation activity profiles of SFN and vehicle-treated platelets (under jump-dilution conditions) in response to ADP (5 μM), thrombin (0.05 U/mL), CRP (0.5 μg/mL), and U46619 (0.5 μM) are shown. (D) Representative light-transmission platelet aggregometry profiles: the ADP aggregation profiles (5 μM) of platelets treated with 20–100 μM SFN through the conventional “direct addition” method. (E) Thrombi formation in 80 μM SFN- or vehicle-treated whole blood at a shear rate of 1800 s^–1^ for 2 min was visualized under a differential interference contrast (DIC) microscope (63×, water objective). SFN inhibits platelet thrombus formation under arterial flow conditions (1800 s^–1^). Relative thrombus volume was measured by labeling the platelets with DiOC6 for 1 h followed by fixation with 2% paraformaldehyde, confocal volumetric imaging (Nikon Eclipse Ti, 40× water objective), and 3D quantification with an NIS-Elements AR.5.21.03 (Figure S7). Volumetric data was shown as mean ± SEM, and an unpaired *t* test was used to compare the treatment.

Here we present a summary of the activity profiles for 23 dietary natural products^21,22,38−48^ (Figure 1), depicted as a heat map to illustrate their effects against four commonly encountered agonists: ADP, thrombin, collagen-related peptide (CRP), and the thromboxane A2 analog U46619 [Figures 2A and S4(A)]. The natural product library of choice includes 18 α,β-unsaturated carbonyl compounds and 5 isothiocyanate compounds, all of which were selected based on their reported antithrombotic roles as constituents of heart-healthy diets, nutraceutical supplements, and herbal medicines.^21,22,38,39^ To account for donor variability, residual platelet activities were normalized to the vehicle control from the same donor. Interestingly, we found that a substantial portion (65%) of these compounds, featuring Michael acceptor functionalities, did not demonstrate inhibitory effects on platelets under our jump-dilution conditions. The observation notably differs from earlier reports,^49,50^ where some natural products exhibited antiplatelet activities when introduced concurrently with an agonist (Table S1). This process, referred to as the “direct addition” method (Figure 2B), implies that their antiplatelet effects are likely to be reversible.

The active samples identified through the “jump-dilution” protocol can be categorized into two main clusters according to their selectivity profiles: cluster 1 includes juglone, and cluster 2 consists of the isothiocyanate family, withaferin A, and xanthohumol. Juglone exhibited a wide range of inhibitory activities, effectively eliminating platelet responsiveness in all aggregometry settings. Our finding aligns with the antiplatelet activity of juglone documented by Wu and colleagues^38^ and further substantiates the notion that juglone irreversibly modifies the platelet proteome, as expected for the highly reactive nature of the quinone motif found in the molecule. In contrast to cluster 1, molecules in cluster 2 displayed selectivity toward certain thrombogenic biochemical stimuli while preserving platelet reactivity to thrombin (0.05–0.1 U/mL). This observation is potentially important, as currently approved antiplatelet agents cannot distinguish signaling initiated by thrombin, the central protease in blood coagulation, from other agonistic pathways.^51^ In particular, the capacity of these agents to impair the activation of the platelet glycoprotein (GP) Ib complex during thrombin-induced platelet aggregation has become an important parameter to evaluate their bleeding risks.^52−55^

### Sulforaphane Manifests a Novel Agonist Selectivity Profile in Platelet Inhibition

When comparing the antiplatelet profiles of natural products in cluster 2 to the aggregation phenotypes obtained through the “direct addition” protocol (Table S1), we were excited to uncover new patterns of antiplatelet effects. These patterns could offer new insights into basic platelet biology as well as therapeutic applications of these electrophilic natural products. For instance, under “direct addition” conditions at a concentration of 20 μM, neither allyl isothiocyanate (AITC) nor phenethyl isothiocyanate (PEITC) had a significant impact on platelet aggregation. However, a dramatic decrease in platelet activity in response to ADP, CRP, and U46619 was noted after preincubation with these natural products for 2 h at the same concentration. The marked differences observed in this comparative analysis underscore the profound impacts that such naturally occurring covalent modifications can have on platelet (patho)physiology.

Among the seven electrophilic natural products in cluster 2, the selectivity exhibited by SFN, a natural product derived from broccoli sprouts, caught our attention: SFN exhibited a unique preference for inhibiting ADP, while its minimal or absent effect on U46619-induced aggregation varied, depending on the donor. Notably, platelet aggregation induced by thrombin and CRP remained unaffected across a wide spectrum of agonist concentrations with the jump-dilution approach (Figure 2A), while previous studies using the direct addition approach have shown significant inhibitory activity.^56^ This ADP selective profile was consistently observed with 12 healthy human donors, spanning both sexes and an age range of 18 to 60 years. Assaying platelets prepared through our “washout” protocol also confirmed the selectivity [Figure S4(B),(C)]. Our subsequent comparative studies with other isothiocyanate natural products revealed the importance of SFN’s sulfoxide center for biological activity. Reducing or oxidizing the sulfoxide moiety to form erucin or erysolin resulted in a decrease in the antiplatelet activity (Figure 2A). Further alterations to the aliphatic chain of SFN may also jeopardize its agonist selectivity, as evidenced by the antiplatelet activity profiles of AITC and PEITC.

### SFN Suppresses Shear-Induced Thrombus Formation

To further investigate the functional influence of SFN under more physiologically relevant conditions, we evaluated the dynamics of platelet adhesion on type I collagen under arterial flow conditions using a microslide perfusion system.^57^ Anticoagulated whole blood was obtained from healthy donors and perfused through collagen-coated microslides at a high arterial shear rate (1800 s^–1^). As illustrated in Figure 2E, during a 2 min perfusion of whole blood, platelets adhered to the collagen-coated surfaces and formed large aggregates in vehicle-treated controls. However, a significant reduction in platelet adhesion and thrombus size was observed when whole blood was treated with 80 μM SFN for 1 h followed by immediate perfusion. The platelets within the thrombi were subsequently labeled with DiOC6, and the total thrombus volume across the entire microslide was quantified, revealing a 64% decrease in thrombus size compared to that of the vehicle-treated controls (Figure S7).

### Leveraging an Alkyne-Integrated Probe of SFN to Map the Covalently Modulated Protein Targets

Given that SFN is known to covalently modify the side-chain functionalities of cysteine and lysine residues within proteins, it is widely recognized as a polypharmacological agent that influences multiple targets simultaneously.^22^ A few protein targets modulated by SFN have been discovered and characterized, providing a preliminary understanding of its chemo-preventive and anti-inflammatory benefits.^58,59^ The antiplatelet activity of SFN was also known to correlate to the PI3K/Akt pathway; however, its role in the modulation of platelet function and prevention of thrombotic disorders at the molecular level has yet to be confirmed.^56,60^

**Figure 3:**
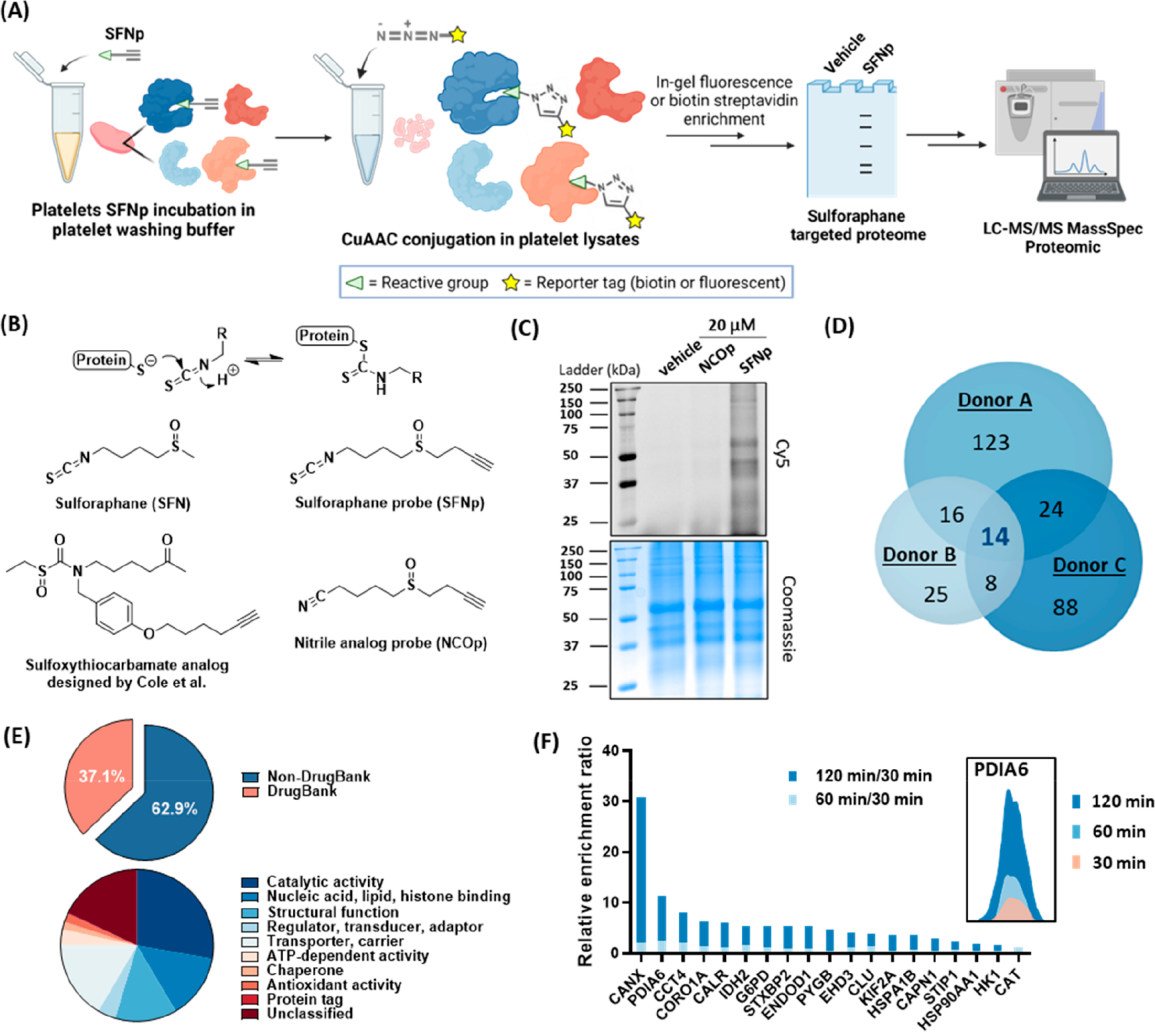
Proteomic mapping of platelet targets covalently modulated by SFN alkyne probe. (A) Schematic illustration showing the treatment of live platelets with SFNp and downstream target analysis. (B) Chemical structures of sulforaphane (SFN), sulfoxythiocarbamate analog probe designed by Cole et al.,^58^ SFN alkyne probe (SFNp), and the nitrile analog probe (NCOp). (C) In-gel fluorescence analysis of proteins revealed from SFNp, NCOp, or vehicle (DMSO) pretreated platelets. The structural analog NCOp (20 μM) without an isothiocyanate functionality was incapable of labeling the proteome as compared to SFNp and the vehicle. Coomassie-stained proteins were used as the loading control. (D) Venn diagrams depict protein targets enriched by SFNp from three healthy adult donors using a biotin–streptavidin affinity purification workflow, followed by in-gel tryptic digestion for label-free quantification LC-MS/MS analysis. Platelets pre-exposed to NCOp (20 μM) or an equivalent volume of vehicle (DMSO) served as controls, with the resulting proteomic samples prepared through the same process. Peptide intensities from the SFNp treatment samples, as determined by mass spectrometry, have been normalized against control samples. Significant hits are defined as those showing a 4-fold enrichment relative to the controls. This Venn diagram specifically outlines these hits relative to the NCOp sample. For significant hits normalized against the vehicle sample, refer to Figure S10. (E) Functional classification of all identified SFNp-enriched protein targets (found in ≥1 donor) based on the DrugBank protein database released on 04/01/2023. (F) Protein disulfide isomerase A6 (PDIA6) was a common significant hit identified in all donor samples (*n* = 4) and exhibited a preponderance in covalent labeling by SFNp compared to the other common hits (found in two to three donors).

While competitive cysteine reactivity profiling and stable isotope labeling by amino acids in conjunction with quantitative mass spectrometry have been utilized to map protein targets of isothiocyanates in cancer cells previously,^61,62^ chemical probe-assisted proteomic profiling presents itself as a promising and robust technique for providing a holistic understanding of the covalently modified proteome.^58,59^ In particular, direct capture of protein targets using an alkyne-tagged analog serves as a gold-standard approach for profiling both on- and off-target effects of covalent drugs.^63−67^ Furthermore, recent technological advancements in clickable chemical probes enable the quantification of target occupancy and the selectivity and measurement of labeling kinetics. This advancement sets the stage for identifying the most sensitive druggable sites based on locale, temporal factors, and disease specificity, which is of great significance for electrophilic drug discovery.^68−74^ Cole and colleagues previously documented the development and utilization of a cell-permeable alkyne-tagged probe to mimic SFN.^58^ In this probe, key functionalities such as isothiocyanate and sulfoxide groups were replaced with sulfoxythiocarbamate and a ketone (Figure 3B). In light of our comparative studies with the isothiocyanate family, which highlight the critical roles of sulfoxide and isothiocyanate functionalities of SFN for agonist selectivity, we aimed to develop SFNp, an SFN-based probe that retains the essential functionalities with an alkyne reporter to facilitate target enrichment and mapping studies. A nitrile analog (NCOp) of the SFNp was also prepared and served as a noncovalent control. It has been shown not to irreversibly bind to proteins under our pull-down conditions (Figure 3B). The antiplatelet activities of SFNp and NCOp were first examined under standard jump-dilution conditions; SFNp phenocopied SFN in light-transmission aggregometry and microslide flow assays, whereas NCOp did not display significant antiplatelet activities in these experiments (Figure S8). To validate the intracellular covalent engagement, live platelets were preincubated with SFNp (20 μM), NCOp (20 μM), or an equivalent volume of vehicle (DMSO) in PWB for varying durations followed by centrifugation and removal of the probe-containing buffer. The resulting platelet pellets were immediately lysed and subjected to Cu(I)-catalyzed azide–alkyne cycloaddition (CuAAC) coupling with Cy5 azide for in-gel fluorescence analysis or with biotin azide for streptavidin-mediated target enrichment. Through in-gel fluorescence analysis, we confirmed the establishment of covalent bonds between SFNp and protein targets through reactions at the isothiocyanate group, as compared to the lack of Cy5 fluorescence signals detected in the proteome treated with NCOp (Figure 3C). On the other hand, biotin-enriched SFNp-modified proteins were first resolved on SDS-PAGE gels, followed by gel excision, in-gel digestion, and label-free quantification (LFQ) through LC-MS/MS analysis to identify the target proteins. The protein signals obtained were normalized against background signals, which were enriched through an identical workflow, except that DMSO (vehicle) or NCOp was employed instead of SFNp (shown as the Abundance Ratio in Supporting Information proteomic data file 1).

We recruited platelet samples from three healthy donors who had not taken antiplatelet medication in the prior 2 weeks for our target mapping studies. Following a 2 h treatment with SFNp, we identified a total of 298 significant hits as compared to the NCOp control (Figure 3D); significant hits are defined as those showing a 4-fold enrichment relative to the control by LFQ. These hits encompass a wide variety of proteins with diverse biological functions, such as catalytic activity, transport functions, and carrier responsibilities (Figure 3E). Although some of these protein functions might not be immediately relevant to platelet (patho)physiology, we found that 63% of these hits have not been previously investigated for drug discovery applications, which underscores the effectiveness of this direct capture of an alkyne-tagged probe in identifying novel targets (Figure 3E). Among the 298 hits, 14 proteins were identified as common hits shared by all 3 donors, some of which are already known to regulate platelet activity (Supporting Information proteomic data file 1). For instance, transforming growth factor β-1 proprotein (TFGB1) is involved in the wound healing and scarring pathway and is released from α-granules upon platelet activation.^75^ However, for the majority of these common hits, their functional roles in platelet-induced thrombosis are still unclear.

### Kinetic Analysis Reveals PDIA6 as a Dominant Responder to SFN in Live Platelets

We anticipated that by comparing the target labeling kinetics of SFN in live platelets, we would be able to identify the genuine target(s) responsible for the time-dependent inhibition of platelet aggregation induced by ADP [Figure S4(B), middle graph]. As such, a new kinetic study was performed, in which a donor’s platelets were incubated with 20 μM SFNp for 30, 60, and 120 min. Subsequently, extensive washing was carried out to remove unbound SFNp, followed by cell lysis and CuAAC conjugation to biotin for enrichment and LC-MS/MS analysis (Figure 3F). The MS1 signals derived from the 30 min labeling sample were employed as a control group for mass spectrometry signal normalization, as the influence on platelet activity at this specific time point was found to be minor. From our analysis, we identified 19 proteins exhibiting a kinetic response to SFNp covalent modification (Figure 3F). Of these, calnexin and protein disulfide isomerase A6 (PDIA6) stood out as major kinetic responders to SFN treatment, showing a more than 10-fold increase in covalent engagement after a 2 h incubation with SFNp in live platelets, compared to the control. Calnexin, an endoplasmic reticulum (ER)-resident chaperone, has a crucial role in folding N-linked glycoproteins within the ER and is known to regulate the biogenesis of integrin α_IIb_β_3_ in megakaryocytes.^76^ However, given that platelets inherit the folded α_IIb_β_3_ from megakaryocytes and possess a limited ability to produce new integrin proteins,^26,27^ the impact of SFN-modified calnexin on platelet adhesion and aggregation via integrin over a short period, such as within the period of 2 h incubation, is expected to be minimal. Therefore, attention was shifted to the second prominent kinetic responder PDIA6, which was consistently identified across all tested donor samples (*n* = 4) via this alkyne probe target profiling approach.

PDIA6, a member of the 20-enzyme thiol isomerase family, primarily facilitates protein folding by catalyzing the formation and cleavage of disulfide bonds in the ER while also acting as a negative regulator of ER stress pathways.^77^ Recent studies have underscored the importance of PDIA6, alongside PDIA1 and PDIA3, in refolding and activating integrins and receptors on the surface of platelets, endothelial cells, and lymphocytes.^78^ In particular, PDIA6 has been found to facilitate thrombus formation in a mouse model of laser-induced thrombosis.^79^ PDIA6, as well as other thiol isomerase enzymes, are characterized by the presence of thioredoxin-like domains,^80,81^ and in PDIA6, three such domains are present: **a**, **a′**, and **b**. Both the **a** and **a′** domains are catalytically active and contain a CGHC motif in the active site. In contrast, the **b** domain lacks this motif but possesses two cysteine residues with functions that have not yet been determined.^82^

### C291 and C297 of PDIA6 Are Isoform-Specific Cysteines Modified by SFN

To determine whether PDIA6 was responsible at least in part for the action of SFN on platelet function that we observed, we first assessed the influence of SFN on PDIA6 structure, function, and activity. We expressed the full-length 48 kDa His6-PDIA6 recombinantly in accordance with the protocol published previously.^78^ Its disulfide-reducing activity was assessed using the standard insulin aggregation assay^83^ before proceeding with Cy5 labeling and mass spectrometry studies.

**Figure 4:**
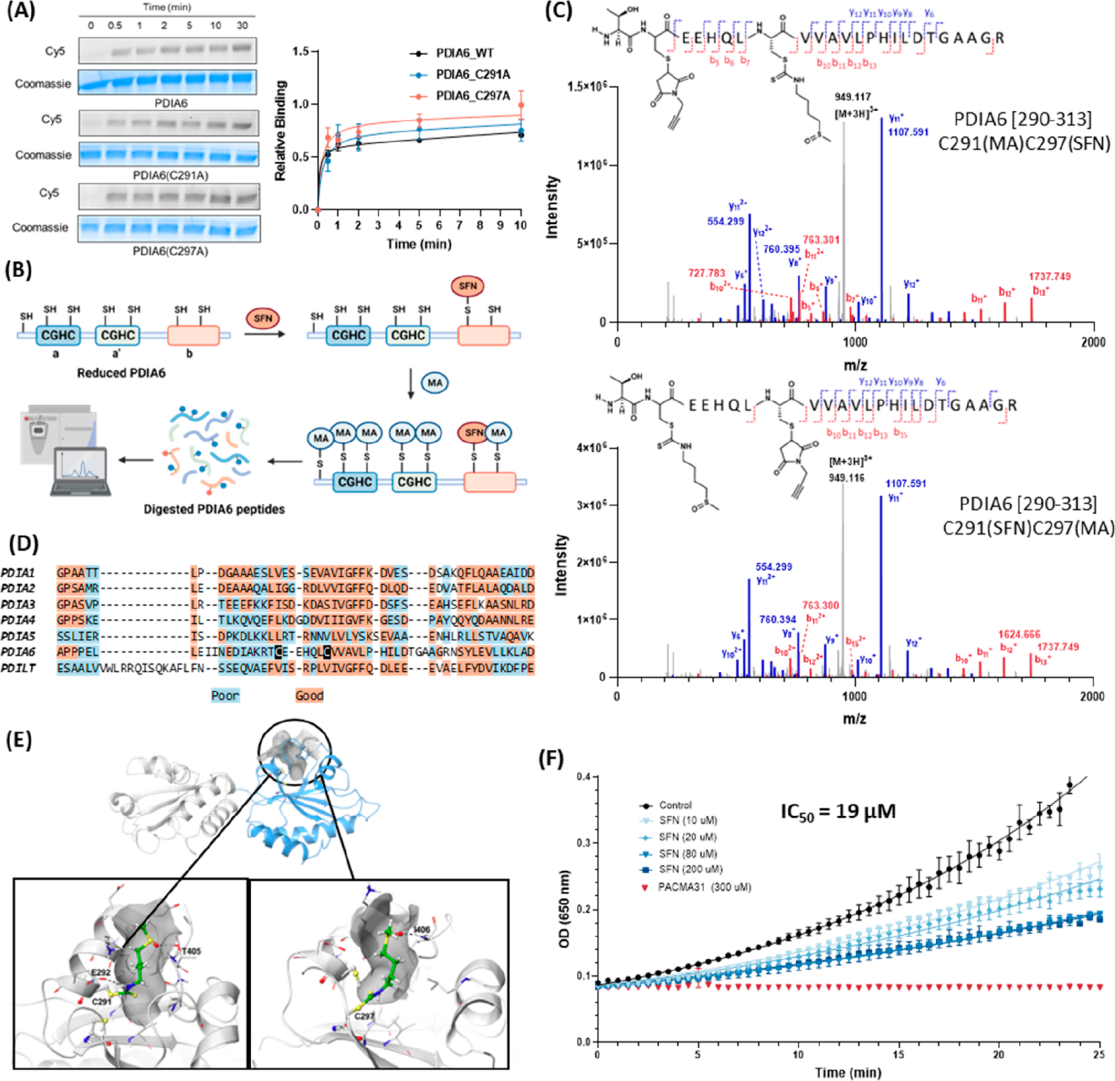
PDIA6 is an isoform-selective and kinetically privileged sensor of SFN. (A) Time-responsive covalent association analysis of PDIA6, PDIA6(C291A), and PDIA6(C297A) labeling by SFNp. Recombinantly expressed PDIA6 was first reduced by DTT (10 mM) for 30 min before undergoing gel filtration on a Zeba Spin desalting column (7 K molecular weight cutoff, 0.5 mL). The resulting PDIA6 (20 μM) was treated with SFNp (25 μM) in 50 mM HEPES/150 mM NaCl (pH 7.4) for varying time intervals (0, 0.5, 1, 2, 5, 10, and 30 min) at 37 °C prior to CuAAC conjugation to Cy5 under denaturing conditions. The relative binding at each time point was normalized upon the respective 30 min Cy5 signal. (B) Schematic illustration of the workflow for PDIA6 modification site identification by LC-MS/MS. Reduced PDIA6 (20 μM, 1.0 equiv) was treated with SFN (22 μM, 1.1 equiv) in 50 mM HEPES/150 mM NaCl (pH 7.4) for 1 h before adding 1 mM *N*-propargylmaleimide (MA). The resulting mixture was subjected to standard trypsin digestion and LC-MS/MS analysis. (C) Representative MS/MS spectra of the PDIA6 [290–313] peptide labeled by SFN and MA at C291 and C297. (D) Sequence alignment of the **b** domain of seven PDI isoforms. SFN-sensing sites (C291 and C297 residues) in PDIA6 are highlighted in black. (E) Molecular modeling of SFN in the potential binding pocket on the **b** domain of PDIA6. Molecular dynamics simulation on the AlphaFold model of the **a′**-**b** domains of PDIA6 revealed a potential binding pocket near the C291 and C297 (gray surface representation). Conformations of SFN (green carbon) covalently linked to either C291 (box on the left) or C297 (box on the right) were predicted through a series of molecular modeling studies, including rigid receptor ligand docking, induced fit docking, and covalent docking calculations. Details of the computational methods are described in Supporting Information Section 4.4. (F) Insulin turbidity assay demonstrated the comparative impact on PDIA6 activity via covalent modulation by SFN and PACMA31, respectively. Reduced PDIA6 (5 μM) was treated with SFN or PACMA31 at varying concentrations (10, 20, 80, 200, and 300 μM) at 37 °C for 1 h before adding to 0.1 M K_2_HPO_4_ (pH 7.0), 2 mM EDTA, 0.13 mM bovine insulin, and 0.33 mM DTT, yielding a final concentration of 100 nM PDIA6.

We began by determining the *in vitro* labeling kinetics. Reduced PDIA6 (20 μM) was incubated with 1.25 equiv of SFNp (25 μM) followed by a 20-fold dilution in denaturing buffer (0.5% SDS, 50 μM GSH, 50 mM HEPES, 150 mM NaCl, pH 7.4) and assayed with CuAAC conjugation to Cy5 azide. Interestingly, we discovered that the covalent reaction between SFN (25 μM) and PDIA6 (20 μM) occurs remarkably rapidly, achieving about 50% labeling within just 30 s (Figure 4A). The observation distinguishes this protein as one of the fastest kinetic sensors for SFN reported to date.

To identify the covalent modification site(s) responsible for such rapid sensing, PACMA31, a covalent pan-PDI inhibitor, was used as a benchmark for targeting the catalytic cysteines within the **a** domain of PDIA6;^84^ it was our initial hypothesis that SFN would target a similar site. Reduced PDIA6 (20 μM) was labeled with 1.1 or 10 equiv of SFN followed by treatment with 1 mM *N*-propargylmaleimide (MA) to cap any unmodified cysteines. To our surprise, the **b**-domain cysteines in PDIA6 emerged as the primary modification sites for SFN (Figure 4B and C). When using 1.1 equiv, either one of the **b**-domain cysteine residues (C291 or C297) was labeled, together with Lys85 without any other off-site labeling identified (Figure S12). Despite being unable to identify the CGHC motif in the **a′** domain, SFN labeling of catalytic cysteine residues within the **a** domain was not observed at either concentration, as determined by comparative analysis with the control sample involving the addition of MA alone or PACMA31 followed by MA capping (Figures S12 and S13). To the best of our knowledge, SFN is the first PDI modulator that covalently modifies noncatalytic cysteines within the **b** domain of PDIA6. Notably, among the seven highly homologous human PDIs, PDIA6 stands out as the sole member containing two cysteines at this specific position (Figure 4D). This discovery raises the possibility of PDI isoform-selective modulation through a naturally occurring covalent modification strategy. Subsequently, we generated the C291A and C297A mutants of PDIA6 and showed that either **b**-domain cysteine can rapidly engage with SFN in a covalent manner, with a labeling half-life of approximately 30 s. Similar to the PDIA6 wild type (WT), the covalent labeling of **b**-domain cysteines reaches a plateau at 30 min based on in-gel Cy5 analysis and comparative MS-based LFQ (Figure 4A and Figure S14)

### Molecular Modeling Studies Reveal the Unique Binding Mode of SFN

Molecular modeling studies were subsequently performed to investigate how SFN may bind to the **b** domain of PDIA6. A 400 ns molecular dynamics (MD) simulation on the AlphaFold model^85^ of the **a′**-**b** domains of human PDIA6 revealed a potential binding pocket near the two cysteine residues of interest. An analysis of the protein surface using SiteMap^86,87^ identified a narrow binding pocket located between the helix consisting of residues 284–292 and the loop comprising residues 403–406 (Figure 4E). A series of ligand docking calculations using the software Glide,^88−90^ including rigid receptor ligand docking and induced fit docking,^91−93^ were conducted (Supporting Information Section 4.4) to refine the binding pocket conformations and optimize interactions with SFN. The final conformations of SFN covalently bound to either C291 or C297 were obtained from covalent docking calculations (Figure S19) and evaluated using MM-GBSA (molecular mechanics with generalized Born and surface area) scoring. MM-GBSA binding energies, representing approximate free energies of binding, were calculated as the difference between the energy of the SFN-PDIA6 complex and the sum of the energies of free SFN and free PDIA6. A more negative value indicates stronger binding. The calculated MM-GBSA binding energies for interactions of SFN with surrounding residues were −37.7 and −34.8 kcal/mol (for SFN bound to C291 and C297, respectively), indicating favorable binding to PDIA6. In line with our comparative studies, the sulfoxide functionality was anticipated to improve site selectivity and binding affinity via polar interactions with the side chains of Glu292 and Thr405 as well as the backbone amide of Ile406 (Figure 4E).

### Bioinformatic Analysis Reveals the Altered Interactome of PDIA6 upon SFN Covalent Labeling and Supports the Agonist Selectivity Profile of SFN

Next, we characterized the impact of C291/C297 covalent modifications on the disulfide isomerase activity. Reduced PDIA6 (5 μM) was preincubated with varying concentrations of SFN (10, 20, 80, and 200 μM) at 37 °C for 1 h, with the intention of establishing the covalent engagement prior to enzymatic assessment. This was followed by a 50-fold dilution in the assay buffer containing 0.1 mM K_2_HPO_4_ (pH 7.0), 2 mM EDTA, 0.13 mM bovine insulin, and 0.1 mM DTT. We observed that SFN exhibited the characteristics of a partial antagonist. At 20 μM, a concentration known to facilitate efficient covalent modification of **b**-domain cysteines, it provided only a modest reduction (50%) in PDIA6 activity (Figure 4F). Furthermore, through in-gel fluorescence experiments, we inferred that the further suppression of PDIA6 (mutant) activity at 200 μM SFN might result from covalent modifications on residues other than C291/C297 (Figures S11 and S20).

**Figure 5:**
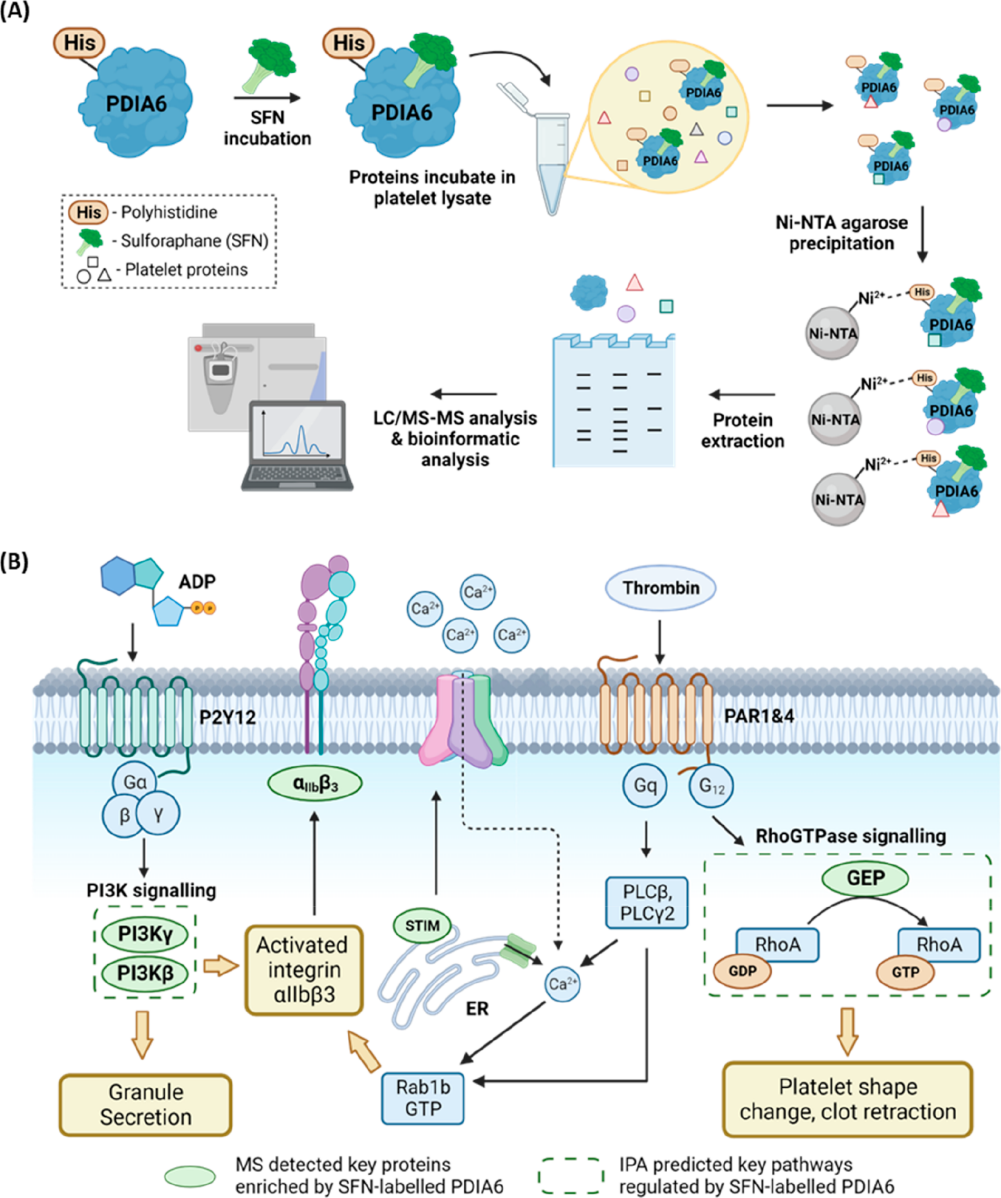
Integrated bioinformatic analysis reveals the pathways modulated by SFN-modified PDIA6. (A) Schematic illustration of the workflow of PDIA6 interactome enrichment. His6-PDIA6 (0.84 μM) was incubated with 20 μM SFN followed by addition to platelet lysates in a standard, non-denaturing lysis buffer. Correlated proteins were enriched on Ni-NTA agarose beads, followed by extraction and resolution on an SDS-PAGE gel. In-gel tryptic digestion followed by LC-MS/MS analysis was conducted to reveal the binding profiles. (B) The reconstruction of protein networks was based on results from IPA as well as literature searching. The relevant signal transduction pathways and selected proteins enriched in these pathways are annotated.

This phenomenon may be attributed to the structural nuances induced by the covalent modification of the **b** domain cysteines, which could influence PDIA6’s substrate preference without eliminating the catalytic activity toward disulfide substrates. Previous studies on kinetically privileged sensor proteins for lipid-derived electrophiles have illustrated that such covalent modifications can potentially lead to the emergence of novel functions, dominant phenotypes, and new organelle localization that may not be fully recapitulated by employing cysteine point mutants.^70,94,95^ We were therefore interested in studying the substrate scope of covalently modified PDIA6 by conducting an interactome coprecipitation experiment using SFN-modified PDIA6 (Figure 5A). His6-PDIA6 protein with or without prior incubation with SFN was utilized to enrich its interactome from platelet lysate on Ni-NTA agarose beads. Following this, the proteins were eluted from the beads and resolved on SDS-PAGE gels for in-gel trypsin digestion and LC-MS/MS analysis. This experiment was replicated on three donor samples (Supporting Information Section 5.1). Our study uncovered clear evidence of a distinct interactome of PDIA6 as a result of covalent modulation by SFN (Figure 5B and Supporting Information Section 5.1).

LC-MS/MS in conjunction with ingenuity pathway analysis (IPA) bioinformatic analysis reveals that the SFN-labeled PDIA6 displays an increased affinity for integrin α_IIb_β_3_ (refer to ITGA2B and ITGB3 in Supporting Information proteomic Table 2). Previous research has emphasized the crucial role of PDI proteins in assisting the refolding of the heterodimeric integrin to its active conformation.^96−98^ Once activated, this conformation readily binds to extracellular matrix proteins, a fundamental step in driving clot formation. During this process, the noncatalytic domains of PDI proteins cooperate with the **a** and **a′** domains to interact with the integrin.^96^ Based on these insights, we postulate that the augmented PDIA6-SFN interaction with α_IIb_β_3_ could impede the refolding mechanism toward the active conformation. To explore this further, we assessed the proportion of platelets adopting the active conformation of α_IIb_β_3_ post-SFN treatment. Upon stimulation with PAR1- and PAR4-receptor agonists, we observed a reduced proportion possessing the active conformation of α_IIb_β_3_ that could be labeled by the FITC-PAC-1 antibody, which supports our hypothesis (Figure S23).

Another notable enrichment was related to the various isoforms of PI3K (Supporting Information proteomic data file 2). In particular, the Gi-coupled ADP receptor P2Y12 is known to facilitate PI3Kβ and PI3Kγ isoform activation upon platelet stimulation, supporting platelet function by stimulating and maintaining integrin α_IIb_β_3_ activation.^99^ Notably, the PI3Kγ isoform has also been found to support platelet activation in a selective manner; the aggregation response to ADP, but not collagen and thrombin, is significantly reduced in platelets deficient in PI3Kγ *in vivo*.^99−101^ Aligning with our hypothesis that SFN increases PDIA6’s affinity for PI3K isoforms, leading to selective modulation of ADP signaling, our flow cytometry experiments revealed a synergistic effect between SFN and the PI3Kβ isoform-specific inhibitor, TGX221. This synergy amplified the antiplatelet action of TGX221 upon PAR1 agonist activation (Figure S23). Our observation also aligns with previous studies where SFN was demonstrated to exhibit characteristics of PI3K inhibitors against platelets under flow conditions.^56^

Our study further reveals that SFN enhances PDIA6’s affinity toward multiple GDP/GTP exchange factors (GEF), potentially influencing RhoA activity, a regulator of platelet contractility and thrombus stability.^102,103^ Reduced RhoA signaling could impact thrombus stability and clot retraction, consistent with the observed decrease in stable platelet aggregate formation under arterial flow conditions (Figure 2E). Our findings align with previous studies that reveal the functional connections between various PDI isoforms and signaling players in RhoGTPase pathways through coimmunoprecipitation studies and conserved gene microsynteny approaches.^104,105^ Moreover, SFN-labeled PDIA6 also exhibits an elevated binding affinity for STIM, implying a potential influence on Ca^2+^ influx, an essential factor governing platelet shape changes, adhesion, and coagulation.^106^

### SFNp Enhances Thrombolysis Efficacy without Causing Excess Bleeding *In Vivo*

**Figure 6:**
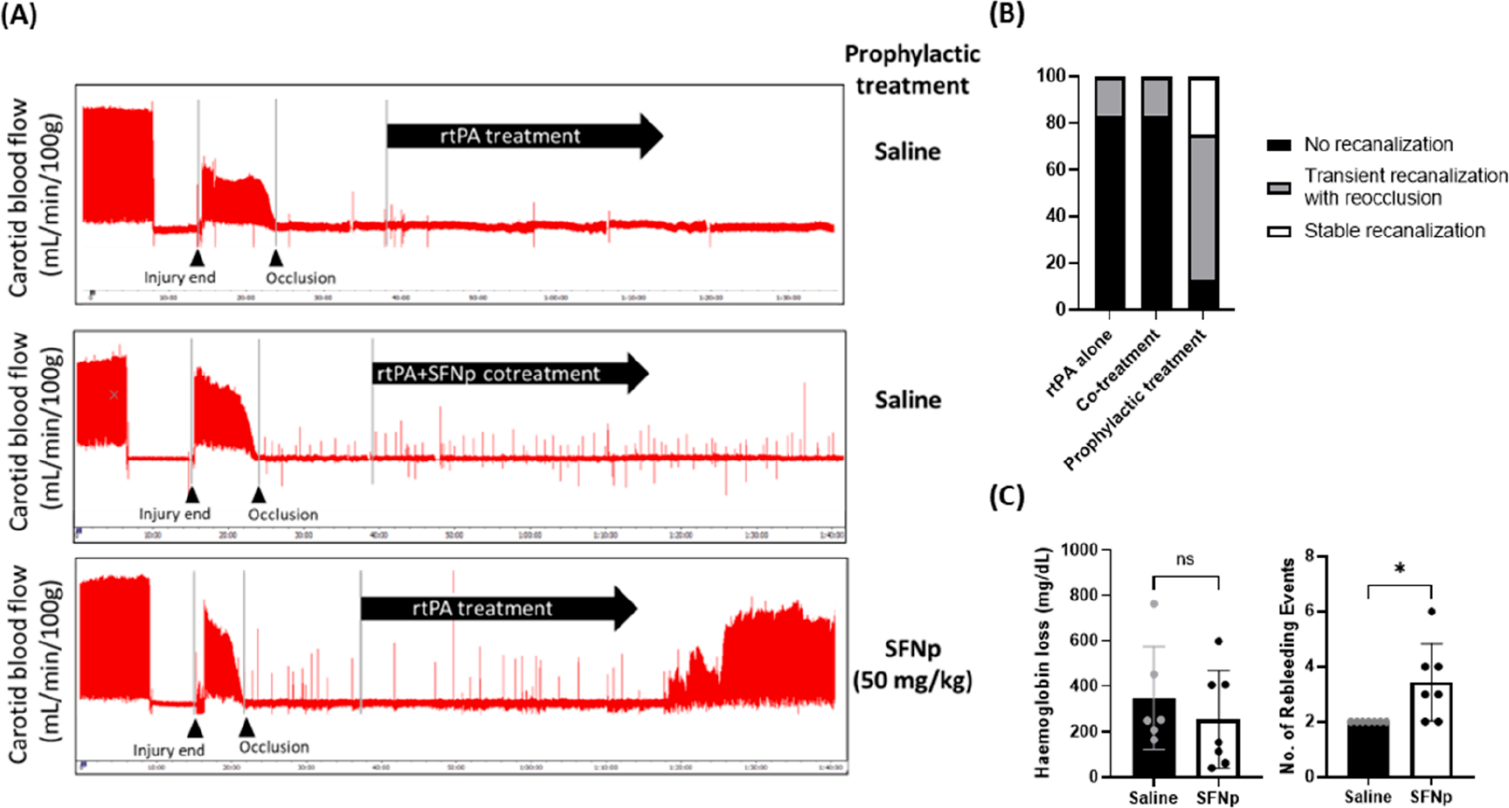
SFNp improves recanalization outcomes prophylactically and does not increase bleeding. (A) Real-time flow traces at the baseline and following electrolytic injury: rtPA treatment administered 15 min after stable occlusion (top panel), cotreatment of rtPA and SFNp given 15 min after stable occlusion (middle panel), and prophylactic SFNp treatment injected 1 h before injury followed by rtPA administration 15 min postinjury (bottom panel). In all scenarios, rtPA was administered via a catheter (1 mg/kg bolus, 9 mg/kg infusion) and SFNp was injected via the femoral vein. (B) Recanalization outcomes of treatment cohorts in (A) were as described. (C) Tail bleeding outcomes demonstrate no significant difference in hemoglobin loss (left) when comparing a treatment with 50 mg/kg of SFNp to an equal volume of saline treatment. The number of rebleeding events (right) when mice were treated with 50 mg/kg SFNp or saline is shown. Statistical significance was calculated via an unpaired *t* test.

Based on our *in vitro* data and bioinformatic analysis of the PDIA6 interactome, we hypothesized that platelets pre-exposed to SFNp would form a less stable clot, rendering it more susceptible to thrombolytic therapy. Therefore, we next focused on investigating the therapeutic synergy between SFNp and thrombolysis with recombinant tissue plasminogen activator (rtPA), the only approved therapy for stroke, utilizing an *in vivo* electrolytic model of thrombosis (Figure 6A). Briefly, C57Bl/6 mice were anaesthetized and subjected to an electrolytic injury (8 mA, 3 min),^107^ forming an occlusive thrombus in the left carotid artery. To assess the effectiveness of SFNp to promote the thrombolytic activity of rtPA, we examined recanalization outcomes compared to those of mice treated with rtPA alone. Recanalization was evaluated by assessing blood flow (mL/min) using a Doppler flow probe and defined and categorized as specified: no recanalization (no return of flow following injury and occlusion), transient recanalization [momentary return of flow after clot formation, followed by reocclusion (no flow)], or stable recanalization (flow returns to the vessel at a level similar to that before the injury and is sustained). Initial studies in untreated mice revealed that recanalization rates achieved with rtPA alone (1 mg/kg bolus, 9 mg/kg infusion) were relatively low, with only 17% of mice demonstrating a transient recanalization event (*n* = 2/12) and the majority of mice (83%) demonstrating sustained occlusion (*n* = 10/12), with no mice demonstrating stable recanalization (0%). Our initial experiments confirmed that without prior exposure to SFNp, coadministration of SFNp (50 mg/kg bolus) with rtPA (1 mg/kg bolus, 9 mg/kg infusion) did not significantly improve carotid recanalization [83% no recanalization (*n* = 5/6), 17% transient recanalization (*n* = 1/6), and 0% stable recanalization] (Figure 6B). In stark contrast, preincubation with SFNp for 1 h prior to electrolytic injury resulted in a significant improvement in recanalization efficacy, with 12.5% no recanalization (*n* = 1/8), 62.5% transient recanalization (*n* = 5/8), and 25% stable recanalization (*n* = 2/8). In comparative studies involving PDIA6 knockout mice (Cre^+^/PDIA6^fl/fl^) and their homozygous PDIA6^fl/fl^ control counterparts, we noted an absence of synergy between prophylactic treatment with SFNp and rtPA in the PDIA6 KO mice (Figure S24). This observation provides confidence in the therapeutic synergy between SFN and tPA occurring through PDIA6 modulation.

Finally, our study also demonstrated that the aforementioned dose (50 mg/kg) of SFNp did not cause excessive blood loss, as assessed through a standard 3 mm tail loop assay,^108^ measuring the amount of hemoglobin lost over the observation period (Figure 6C). Interestingly, this was despite an equivalent or additional number of rebleeding events compared to saline-treated mice (Figure 6C), which may relate to the stability of the blood clot. This data affirms the *in vivo* safety of the SFN mimic SFNp alongside its thrombolytic efficacy in a murine model of thrombolysis.

## Conclusions

The pursuit of selective intervention with platelet-mediated thrombus formation without disturbing hemostatic balance is a hot topic in cardiovascular research and an ongoing design challenge intimately associated with the intertwined signaling pathways underlying platelet activation. In this study, we combined a streamlined cell preparation method with the antiplatelet phenotyping of electrophilic phytochemicals to uncover previously unknown agonist selectivity profiles. These profiles are associated with the impact of electrophilic protein modifications induced by these phytochemicals, thereby revealing new insights into their mode of action. In particular, by combining chemoproteomics, molecular simulation, and mass spectrometry analysis, we demonstrated that SFN serves as a novel chemotype for targeting PDIA6 in platelets, with mapping of the covalent modification sites revealing unparalleled levels of PDI isoform selectivity. Through interactome coprecipitation in conjunction with bioinformatic analysis, we elucidated the impact of SFN on PDIA6’s substrate preference, which aligns with the observed *in vitro* and *in vivo* antiplatelet phenotypes. Importantly, SFN displayed important characteristics of prophylactic agents and was able to improve the clot-busting performance of rtPA in an *in vivo* electrolytic injury model of thrombosis. These results provide new insights into the studies of the molecular pharmacology of naturally occurring isothiocyanates as novel antithrombotic leads, particularly in combination with approved therapies. Our findings together with previous reports^22,58,59^ on SFN’s roles in suppressing neuroinflammation and oxidative stress provide the impetus to investigate the molecular mechanisms underlying dietary antiplatelets with a view to discovering novel preventive and therapeutic mechanisms for thrombosis and strokes without significant bleeding risks.

## Supporting Information Available

^1^H and ^13^C NMR spectra (PDF)
Human platelet sample preparation and assessment protocols, *in vitro* biological assays, chemical biological methods performed in cells and in mice, proteome profiling and bioinformatic analysis methods, molecular modeling, chemical synthesis procedures, compound characterization data, and figure legends for Figures S21–S24 (PDF)
Donors A–C and a kinetic study (XLSX)
Original list of all proteins identified using label-free shotgun proteomics, list of all proteins confidently identified for ingenuity pathway analysis, heat map visualization of all dramatically changed proteins in the proteome of SFN+PA16 vs PA16 only, list of protein pathways related to all protein changes in the proteome of SFN+PA16 vs PA16 only, list of IPA predicted upstream regulators related to all protein changes in the proteome of SFN+PA16 vs PA16 only, and heat map visualization of the expression of PIK3 and ITG familes in the proteome of SFN+PA16 vs PA16 only (XLSX)
