# Supplementary material for: Integrating Phenotypic and Chemoproteomic Approaches to Identify Covalent Targets of Dietary Electrophiles in Platelets
Source: ACS Cent Sci. 2024 Jan 29;10(2):344–57. doi: 10.1021/acscentsci.3c00822 (PMC10906253; doi:10.1021/acscentsci.3c00822)
Supplement: Supplementary file 1 — oc3c00822_si_001.pdf [file oc3c00822_si_001.pdf]

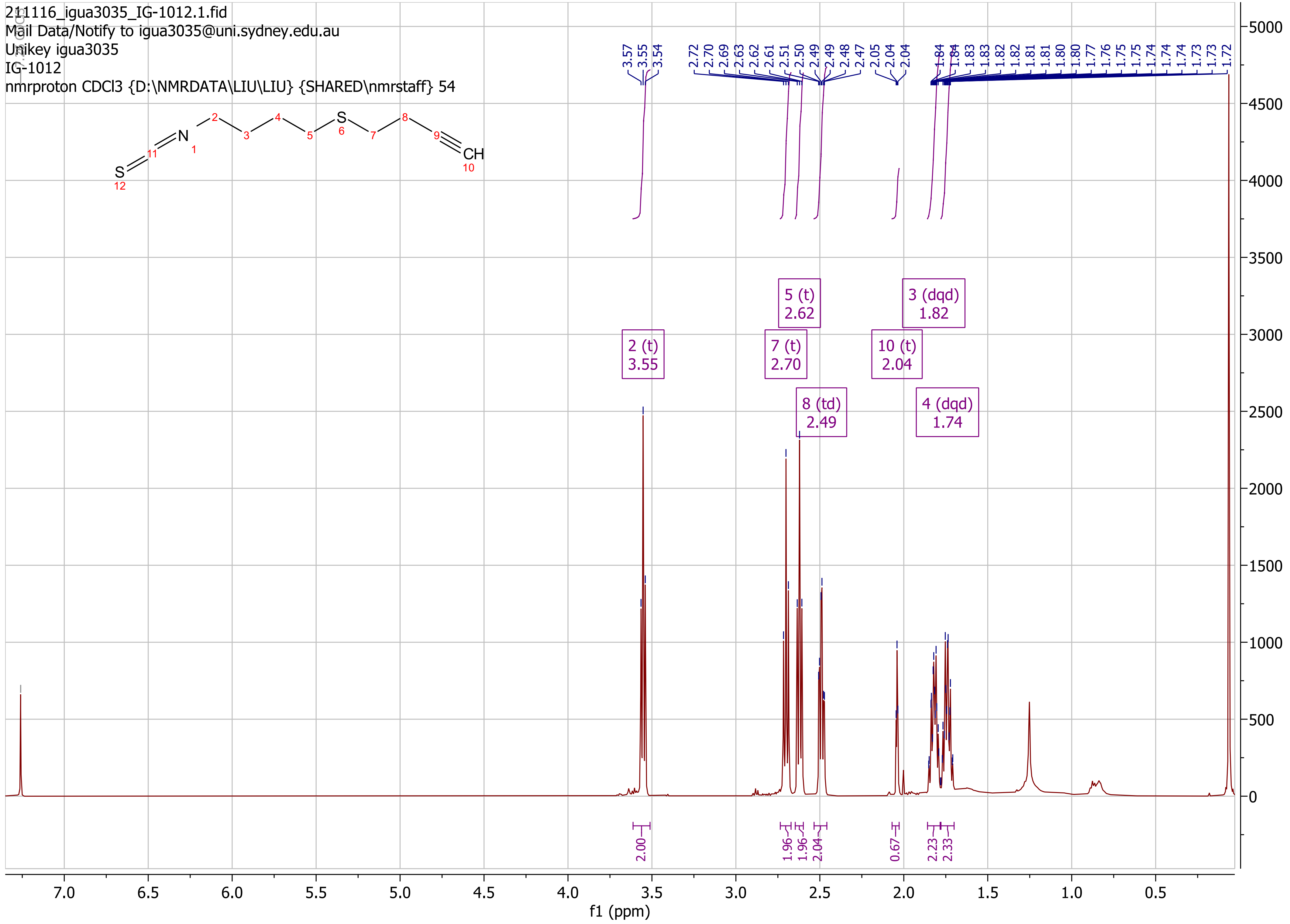

211116_igua3035_IG-1012.1.fid
Mail Data/Notify to igua3035@uni.sydney.edu.au
Unikey igua3035
IG-1012
nmrproton CDCl3 {D:\NMRDATA\LIU\LIU} {SHARED\nmrstaff} 54
3.57 3.55 3.54
2.72 2.70 2.69 2.63 2.62 2.61 2.51 2.50 2.49 2.49 2.48 2.47
2.05 2.04 2.04
1.84 1.84 1.83 1.83 1.82 1.82 1.81 1.81 1.80 1.80 1.77 1.76 1.75 1.75 1.74 1.74 1.74 1.73 1.73 1.72
S
N
CH
1 2 3 4 5 6 7 8 9 10 11 12
2 (t)
3.55
7 (t)
2.70
5 (t)
2.62
8 (td)
2.49
10 (t)
2.04
3 (dqd)
1.82
4 (dqd)
1.74
2.00
1.96
1.96
2.04
0.67
2.23
2.33
7.0 6.5 6.0 5.5 5.0 4.5 4.0 3.5 3.0 2.5 2.0 1.5 1.0 0.5
f1 (ppm)
5000 4500 4000 3500 3000 2500 2000 1500 1000 500 0

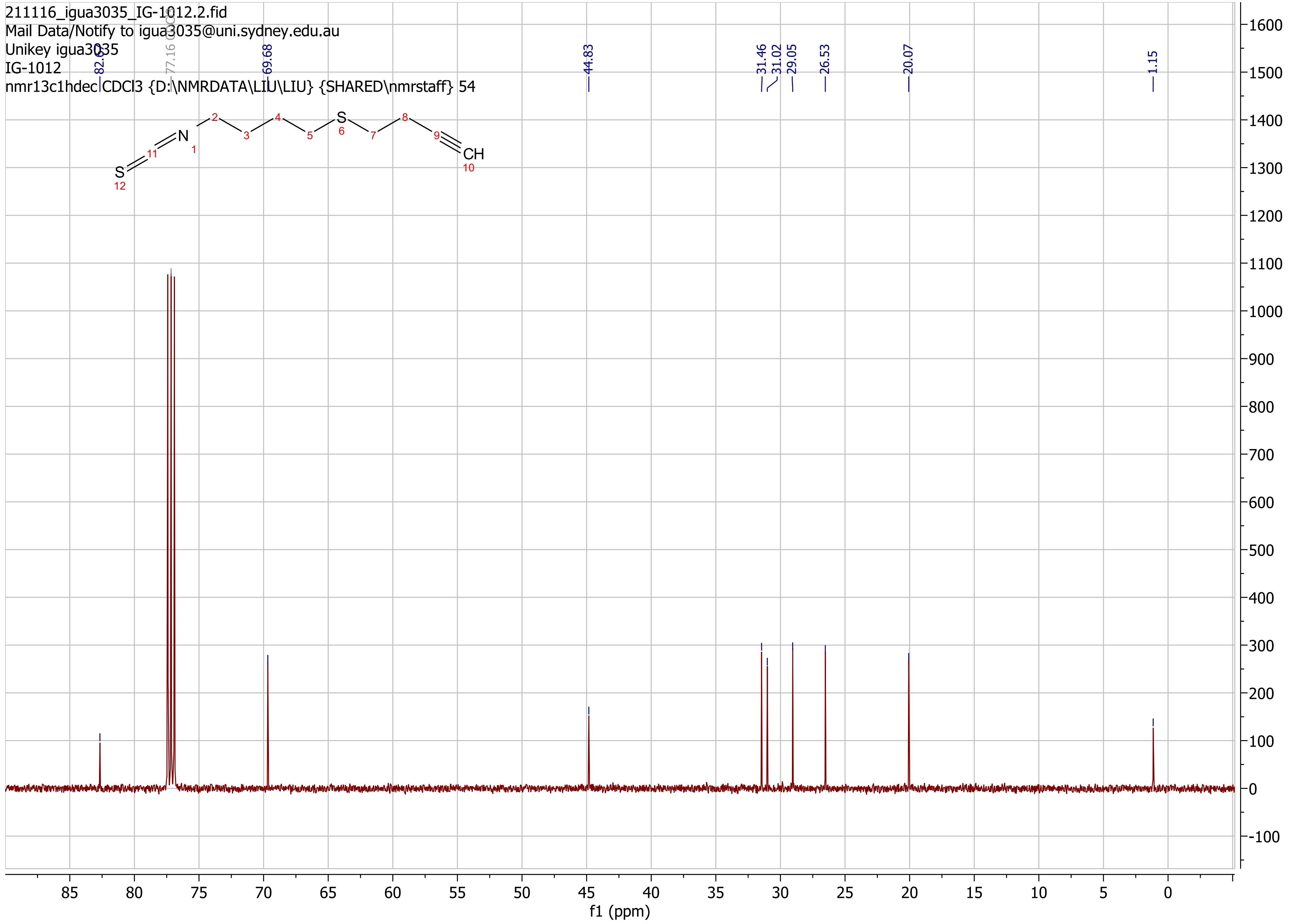

211116_igua3035_IG-1012.2.fid
Mail Data/Notify to igua3035@uni.sydney.edu.au
Unikey igua3035
IG-1012
nmr13c1hdec CDCl3 {D:\NMRDATA\LIU\LIU} {SHARED\nmrstaff} 54
77.16 CDCl3
69.68
44.83
31.46
31.02
29.05
26.53
20.07
1.15
S
N
CH
f1 (ppm)

Generic Display Report

Analysis Info

Analysis Name	D:\OneDrive - The University of Sydney (Staff)\Instrument_Data\BrukerSolarix2XR\Data\MS Facility\Chianna Dane\20220728\20220728_Service_HiRes_ESI_000018.d	Acquisition Date	7/28/2022 8:32:43 AM
Method	20220728 ESI Pos Neg 150-3000 4M-updatedshim	Operator	Admin
Sample Name	But-3-yn-1-yl(4-isothiocyanatobutyl)sulfane (Compound 4)	Instrument	solariX 2xR
Comment	MeOH		

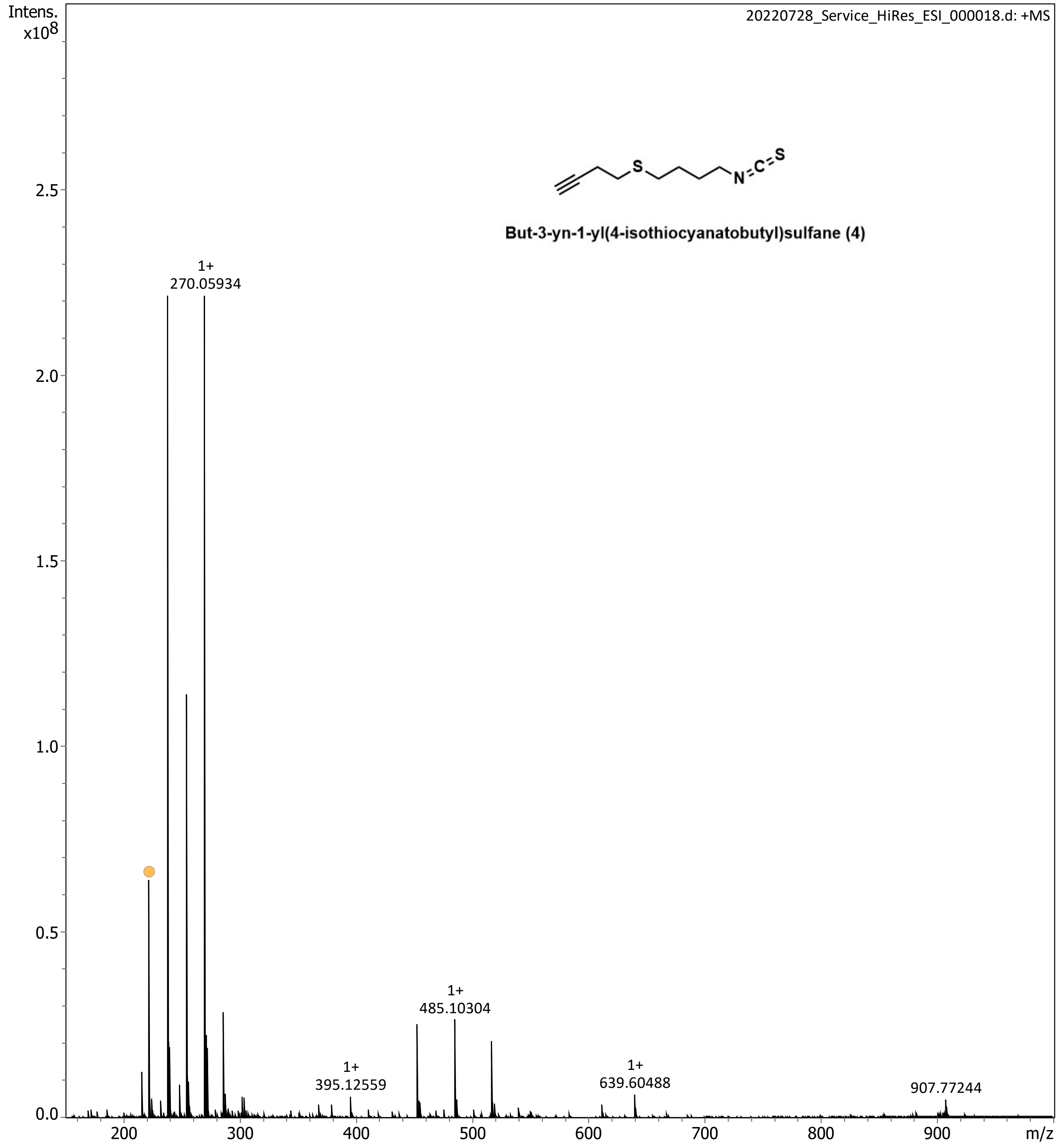

But-3-yn-1-yl(4-isothiocyanatobutyl)sulfane (4)

Generic Display Report

Method	ESI Pos Neg 150-3000 4M-updatedshim	Operator	Admin
Sample Name	But-3-yn-1-yl(4-isothiocyanatobutyl)sulfane (Compound 4)	Instrument	solariX 2xR
Comment	MeOH		

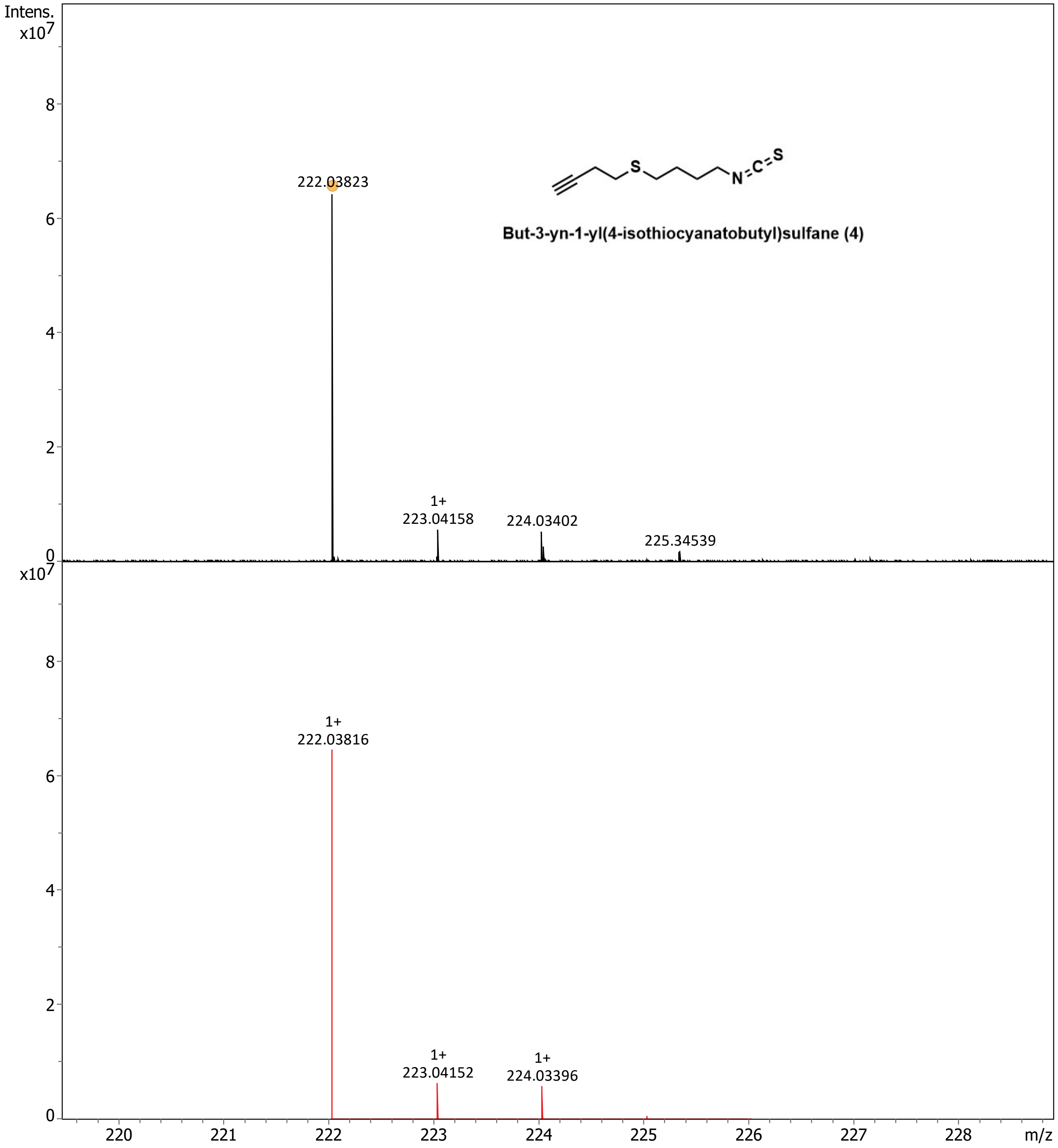

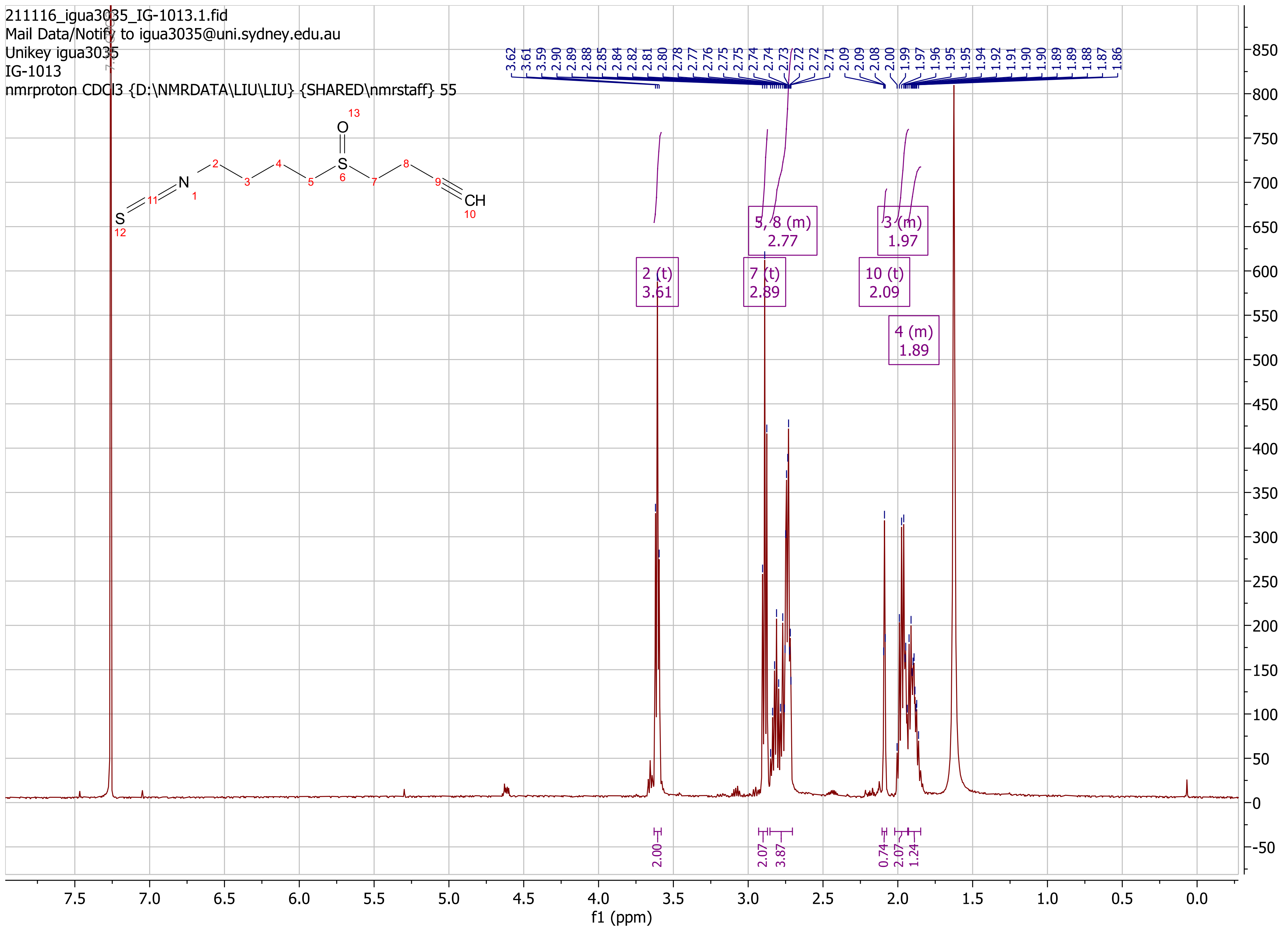
211116_igua3035_IG-1013.1.fid
Mail Data/Notify to igua3035@uni.sydney.edu.au
Unikey igua3035
IG-1013
nmrproton CDCl3 {D:\NMRDATA\LIU\LIU} {SHARED\nmrstaff} 55
3.62 3.61 3.59 2.90 2.89 2.88 2.85 2.84 2.82 2.81 2.80 2.78 2.77 2.76 2.75 2.75 2.74 2.74 2.73 2.72 2.72 2.71 2.09 2.09 2.08 2.00 1.99 1.97 1.96 1.95 1.95 1.94 1.92 1.91 1.90 1.90 1.89 1.89 1.88 1.87 1.86
2 (t) 3.61
7 (t) 2.89
5, 8 (m) 2.77
10 (t) 2.09
3 (m) 1.97
4 (m) 1.89
2.00
2.07
3.87
0.74
2.07
1.24
f1 (ppm)

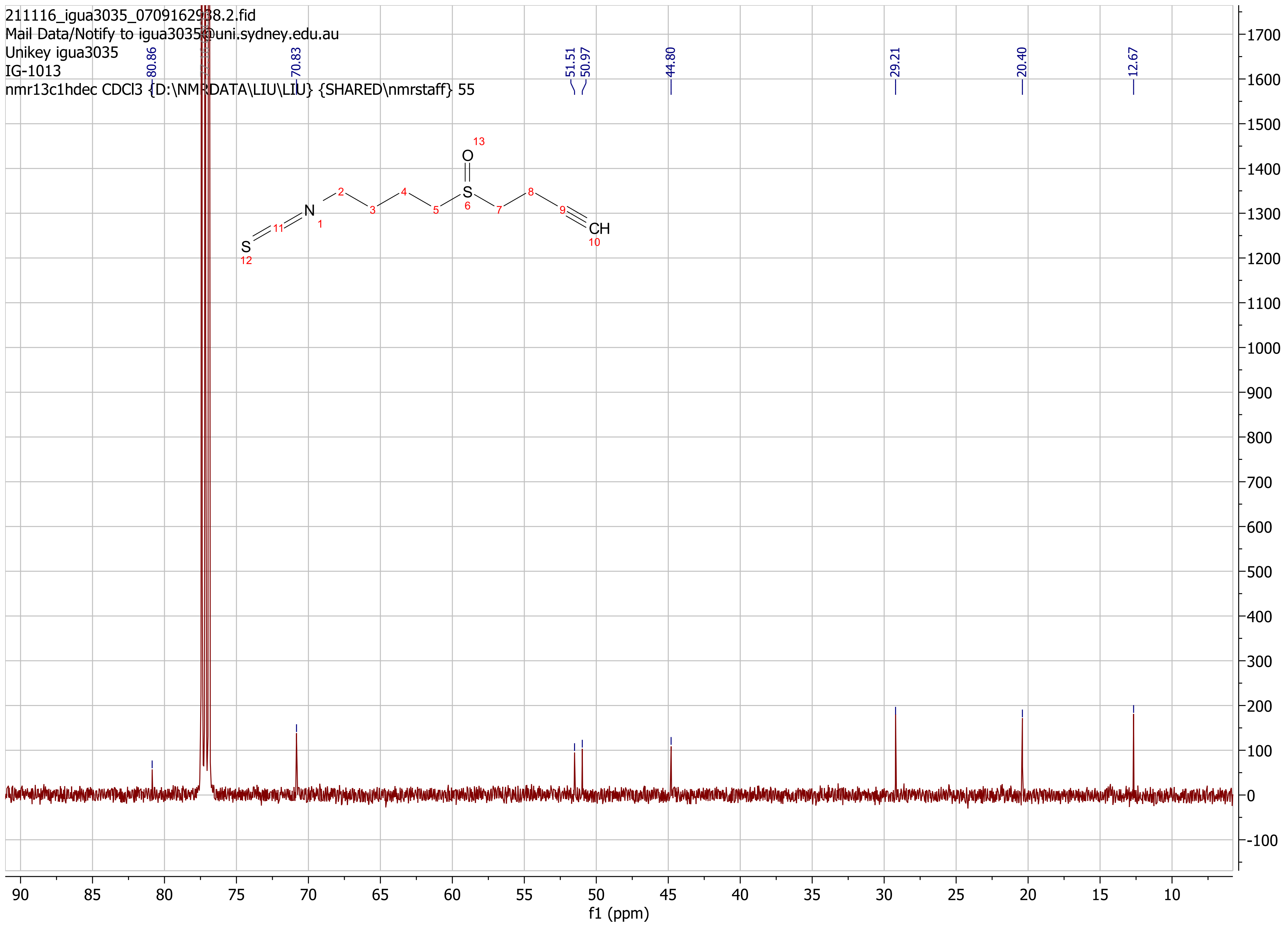

211116_igua3035_0709162938.2.fid
Mail Data/Notify to igua3035@uni.sydney.edu.au
Unikey igua3035
IG-1013
nmr13c1hdec CDCl3 {D:\NMRDATA\LIU\LIU} {SHARED\nmrstaff} 55
80.86
70.83
51.51
50.97
44.80
29.21
20.40
12.67
O
S
N
S
CH
1
2
3
4
5
6
7
8
9
10
11
12
13
f1 (ppm)

Generic Display Report

Method	ESI Pos Neg 150-3000 4M-updatedshim	Operator	Admin
Sample Name	4-((4-Isothiocyanatobutyl)sulfinyl)but-1-yne (Compound 2)	Instrument	solariX 2xR
Comment	MeOH		

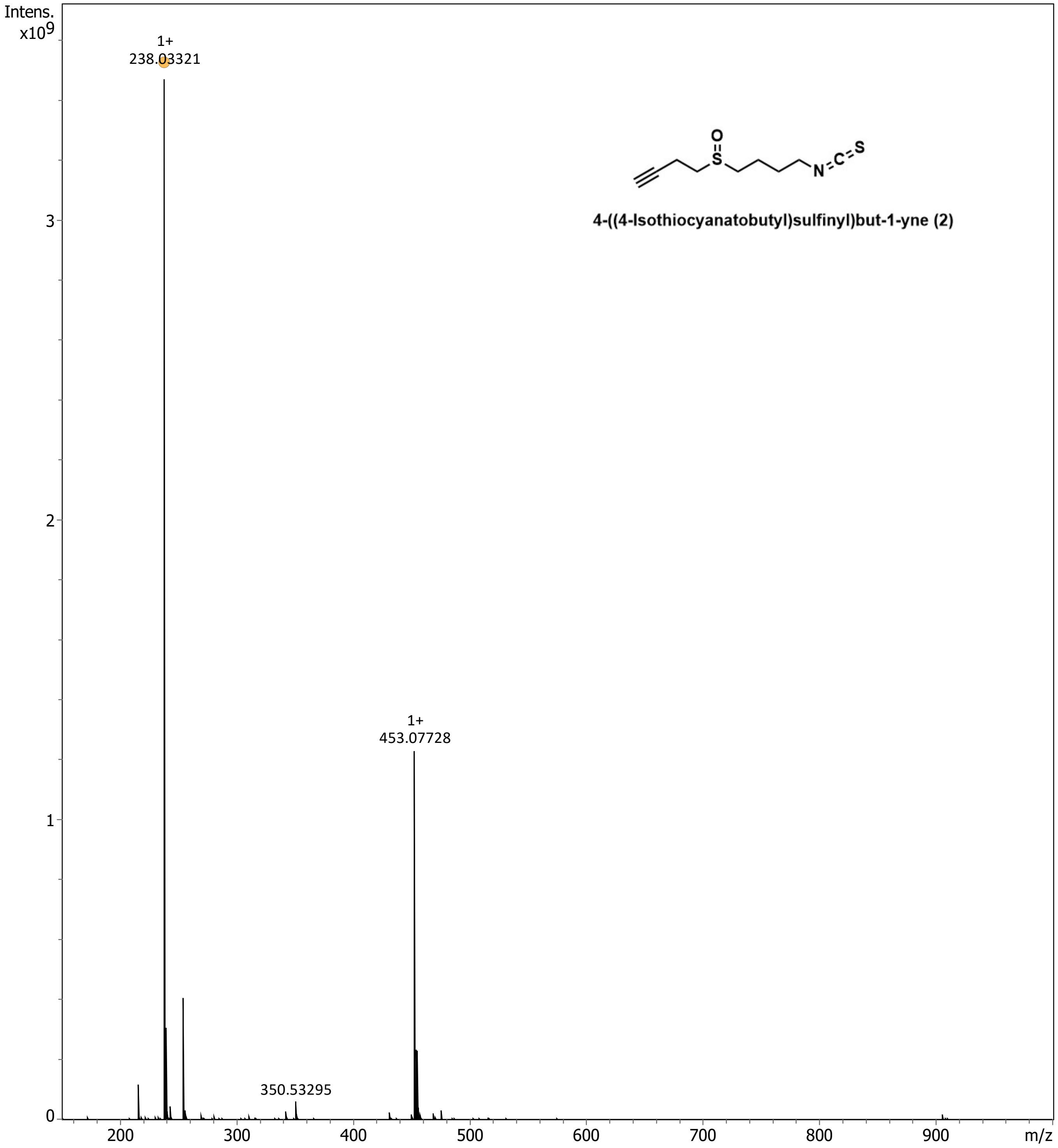

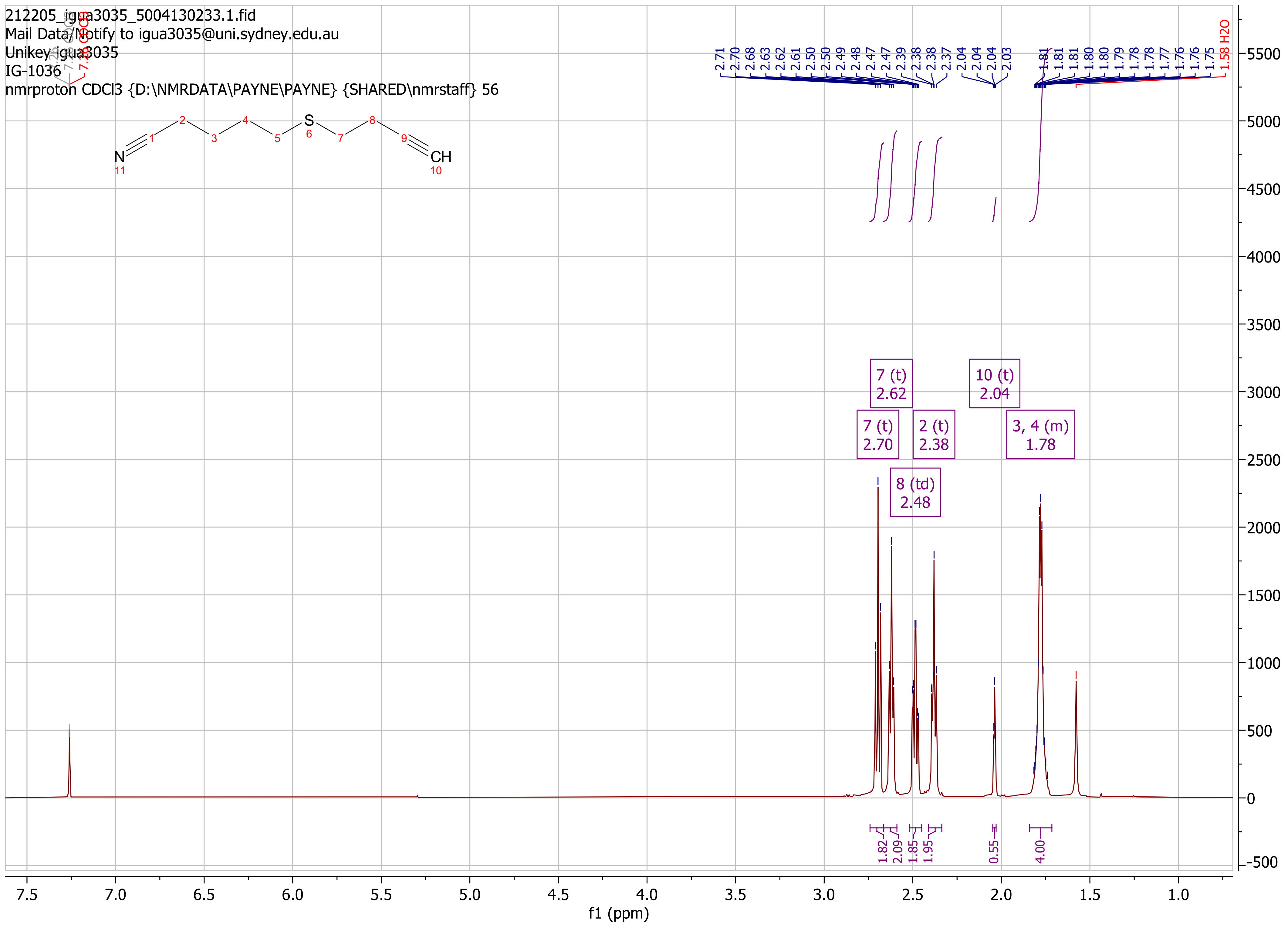
212205_igua3035_5004130233.1.fid
Mail Data/Notify to igua3035@uni.sydney.edu.au
Unikey igua3035
IG-1036
nmrproton CDCl3 {D:\NMRDATA\PAYNE\PAYNE} {SHARED\nmrstaff} 56
7.26 CDCl3
2.71
2.70
2.68
2.63
2.62
2.61
2.50
2.50
2.49
2.48
2.47
2.47
2.39
2.38
2.38
2.37
2.04
2.04
2.04
2.03
1.81
1.81
1.81
1.80
1.80
1.79
1.78
1.78
1.77
1.76
1.76
1.75
1.58 H2O
N
CH
S
7 (t)
2.62
7 (t)
2.70
2 (t)
2.38
8 (td)
2.48
10 (t)
2.04
3, 4 (m)
1.78
1.82
2.09
1.85
1.95
0.55
4.00
f1 (ppm)

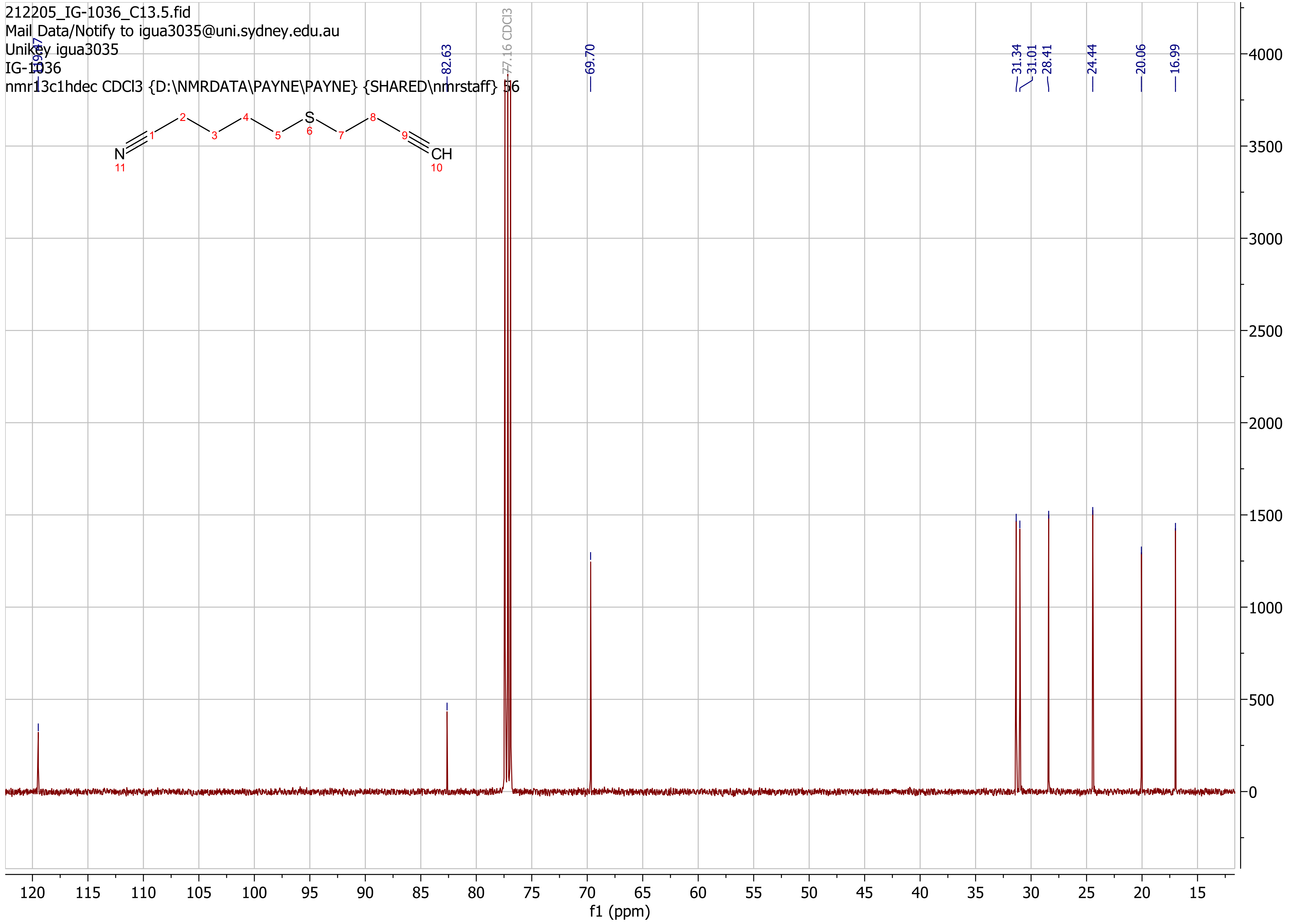

212205_IG-1036_C13.5.fid
Mail Data/Notify to igua3035@uni.sydney.edu.au
Unikey igua3035
IG-1036
nmr13c1hdec CDCl3 {D:\NMRDATA\PAYNE\PAYNE} {SHARED\nmrstaff} 56
N
CH
S
1 2 3 4 5 6 7 8 9 10 11
82.63
77.16 CDCl3
69.70
31.34
31.01
28.41
24.44
20.06
16.99
f1 (ppm)

Generic Display Report

Method	ESI Pos Neg 150-3000 4M-updatedshim	Operator	Admin
Sample Name	5-(But-3-yn-1-ylthio)pentanenitrile (Compound 5)	Instrument	solariX 2xR
Comment	MeOH		

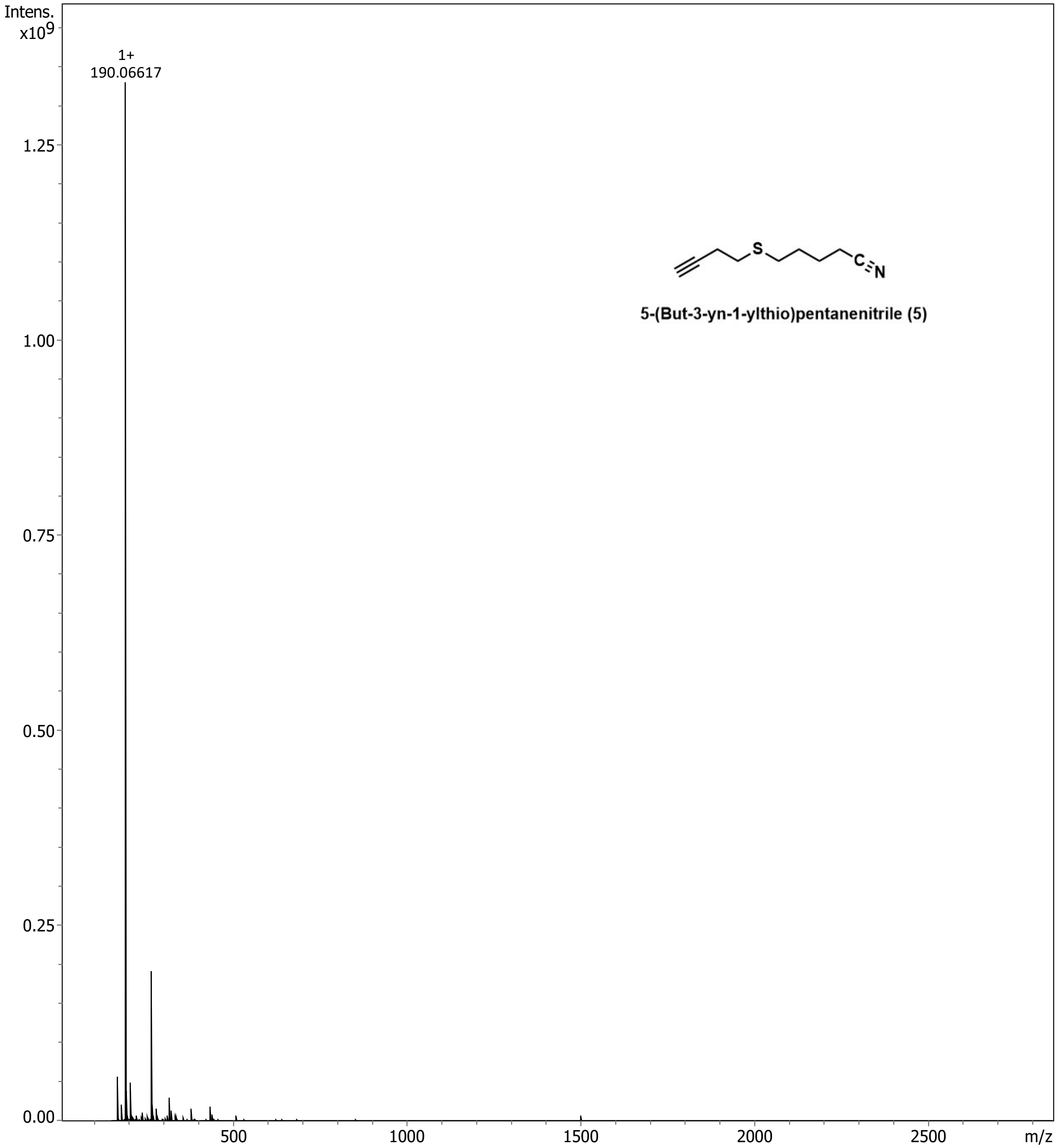

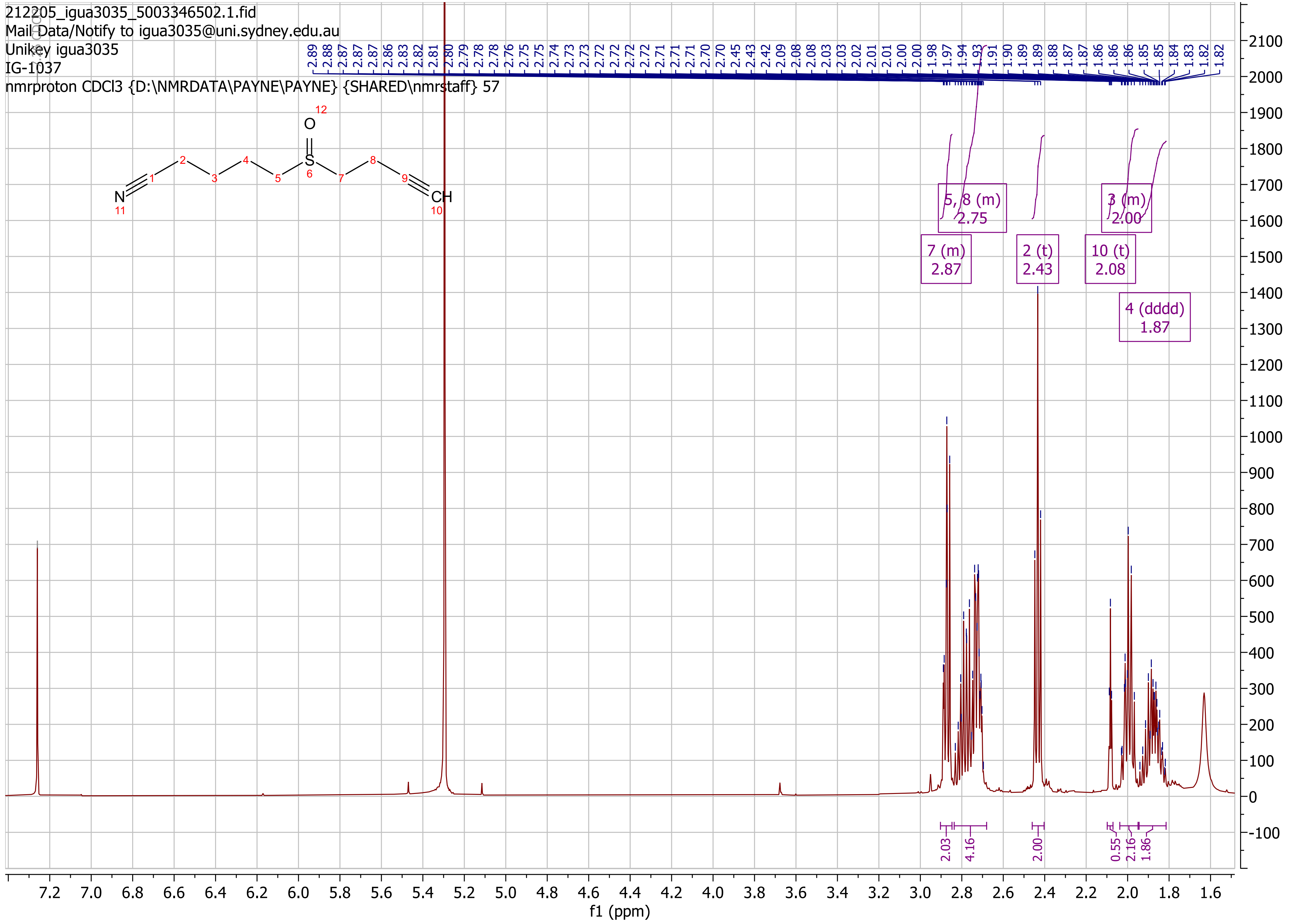
212205_igua3035_5003346502.1.fid
Mail Data/Notify to igua3035@uni.sydney.edu.au
Unikey igua3035
IG-1037
nmrproton CDCl3 {D:\NMRDATA\PAYNE\PAYNE} {SHARED\nmrstaff} 57
5, 8 (m)
2.75
7 (m)
2.87
2 (t)
2.43
3 (m)
2.00
10 (t)
2.08
4 (dddd)
1.87
2.03
4.16
2.00
0.55
2.16
1.86
f1 (ppm)

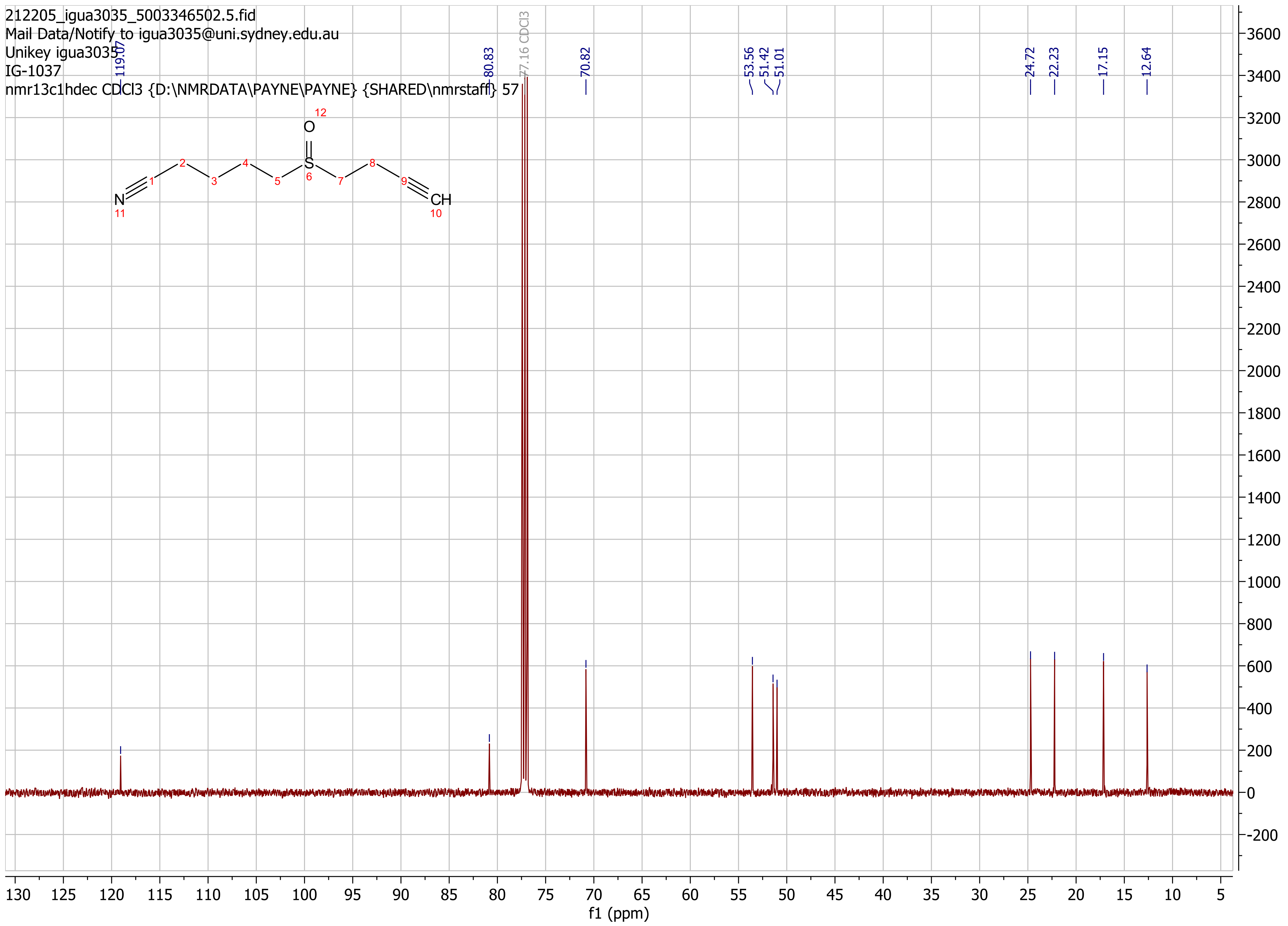
212205_igua3035_5003346502.5.fid
Mail Data/Notify to igua3035@uni.sydney.edu.au
Unikey igua3035
IG-1037
nmr13c1hdec CDCl3 {D:\NMRDATA\PAYNE\PAYNE} {SHARED\nmrstaff} 57
119.07
80.83
77.16 CDCl3
70.82
53.56
51.42
51.01
24.72
22.23
17.15
12.64
N
CH
O
S
f1 (ppm)

Generic Display Report

Method	ESI Pos Neg 150-3000 4M-updatedshim	Operator	Admin
Sample Name	5-(But-3-yn-1-ylsulfinyl)pentanenitrile (Compound 6)	Instrument	solariX 2xR
Comment	MeOH		

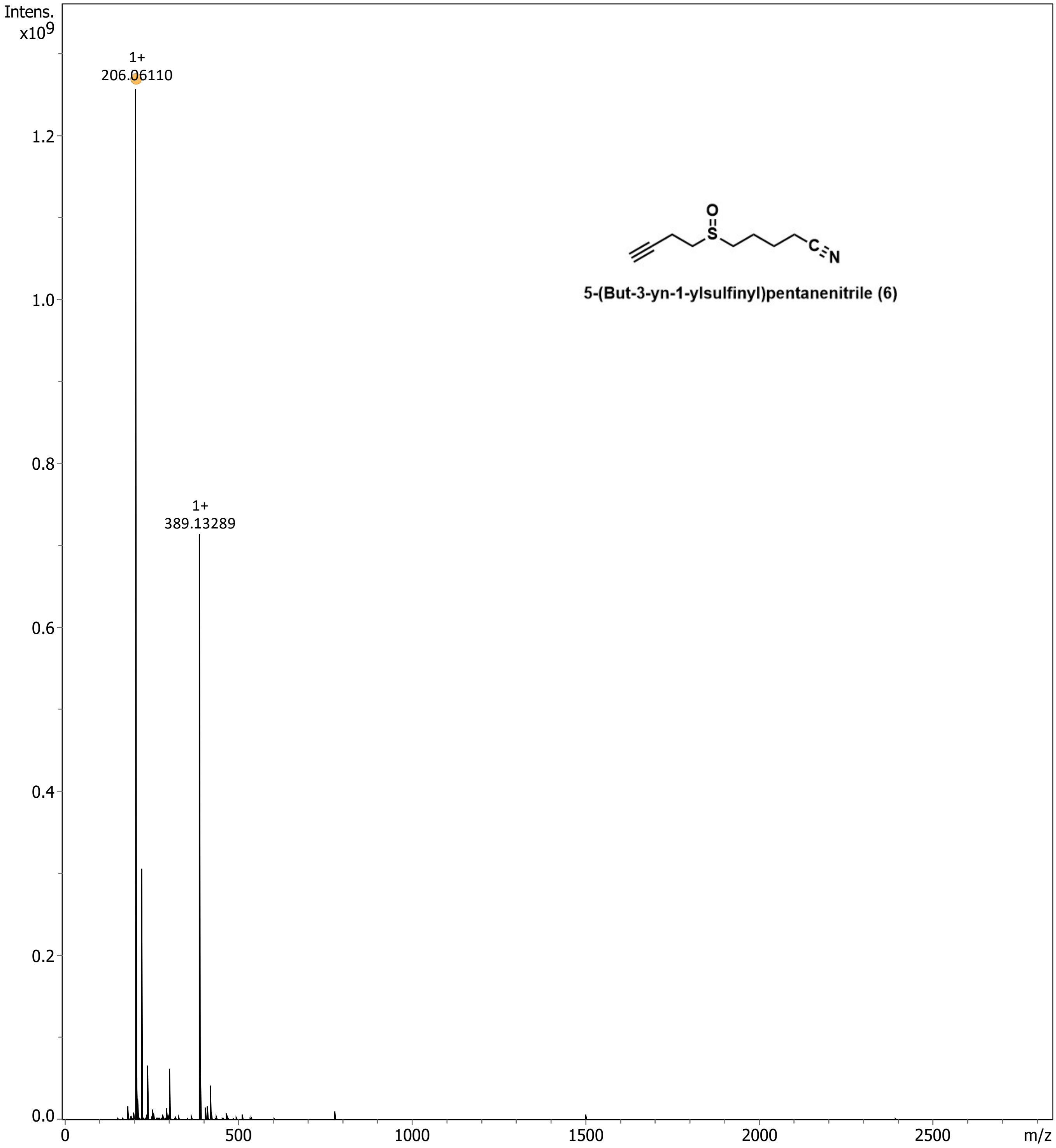

5-(But-3-yn-1-ylsulfinyl)pentanenitrile (6)
