# Supplementary material for: Integrating Phenotypic and Chemoproteomic Approaches to Identify Covalent Targets of Dietary Electrophiles in Platelets
Source: ACS Cent Sci. 2024 Jan 29;10(2):344–57. doi: 10.1021/acscentsci.3c00822 (PMC10906253; doi:10.1021/acscentsci.3c00822)
Supplement: Supplementary file 2 — oc3c00822_si_002.pdf [file oc3c00822_si_002.pdf]

Integrating Phenotypic and Chemoproteomic Approaches to Identify Covalent Targets of Dietary Electrophiles in Platelets

Supporting Information

Ivy A. Guan[1,2], Joanna S.T. Liu[2,3], Renata C. Sawyer[1,2], Xiang Li[4,5], Wanting Jiao[6], Yannasittha Jiramongkol[1,8], Mark D. White[1], Lejla Hagimola[3], Freda H. Passam[3], Denise P. Tran[7], Xiaoming Liu[3], Simone M. Schoenwaelder[2,3], Shaun P. Jackson[2,8], Richard J. Payne[1,9], Xuyu Liu[1,2]*

* Corresponding author. Email: xuyu.liu@sydney.edu.au

[1]School of Chemistry, Faculty of Science, The University of Sydney, Sydney, New South Wales 2006, Australia

[2]The Heart Research Institute, The University of Sydney, Newtown, New South Wales 2042, Australia

[3]School of Medical Sciences, Faculty of Medicine and Health, The University of Sydney, Sydney, New South Wales 2006, Australia

[4]Department of Medicine, Washington University in St. Louis, St. Louis, MO 63110, United States

[5]McDonnell Genome Institute, Washington University in St. Louis, St. Louis, MO 63108, United States

[6]Ferrier Research Institute, Victoria University of Wellington, Wellington 6140, New Zealand; Maurice Wilkins Centre for Molecular Biodiscovery, Auckland 1142, New Zealand.

[7]Sydney Mass Spectrometry, The University of Sydney, Camperdown, New South Wales 2006, Australia

[8]Charles Perkins Centre, The University of Sydney, Sydney, New South Wales 2006, Australia

[9]Australian Research Council Centre of Excellence for Innovations in Peptide and Protein Science, The University of Sydney, Sydney, New South Wales 2006, Australia

Section 1. General Material and Methods

Section 1.1 Chemical and Biological Reagents

The following natural products: murrayone (CFN98505), nardosinone (CFN90178), piperine (CFN99919), pellitorine (CFN96143), ethyl ferulate (CFN99767), praeruptorin A (CFN98139), praeruptorin C (CFN98143), sophocarpine (CFN99182), withaferin A (CFN91895), isoliquiritigenin (CFN97542), baicalein (CFN98783), cornin (CFN98160), myricetin (CFN98877), xanthohumol (CFN98958), nootkatone (CFN98699), (*E*,*Z*)-guggulsterone (CFN93007), and visnagen (CFN97314) were purchased from ChemFaces. Stocks of the natural products were prepared at 80 mM and 20 mM concentrations in dimethyl sulfoxide (D4540-1L, Sigma Aldrich) and stored at -80 °C until assayed. Sulforaphane (10496) and erucin (14017) were obtained from Cayman. PACMA-31 (SML0838), 5-hydroxy-1,4-naphthoquinone (H47003-1G, juglone), allyl isothiocyanate (36682-1G), and phenethyl isothiocyanate (253731-5G) were purchased from Sigma Aldrich. The agonists employed in the light transmission aggregometry studies included adenosine 5'-diphosphate (ADP) (A4386, Sigma Aldrich), thrombin (10445720; Dade Bovine Thrombin 10 x 1 mL; Siemens), U46619 (thromboxane A_2 receptor agonist, 16450-5MG, Sapphire Bioscience), collagen related peptide (CRP) (synthesized by Dr D. Bihan, Department of Biochemistry, University of Cambridge). The fibrinogen additive used in the light transmission aggregometry assay was sourced from Sigma Aldrich. Reagents involved in the human whole blood and platelet washing steps include Clexane 100 mg/mL (anti-Xa: 10,0000 IU) purchased from SANOFI, 500 U/mL Apyrase (Grade VII) (A6535) was prepared based on ADPase activity into 50 µL aliquots of 0.5, 1, and 1.5 U/mL using ice-cold Tyrode's buffer and 0.05% bovine serum albumin (BSA) to prevent any loss of apyrase in tubes. Sodium citrate stock was made into 3.8% solution using sodium citrate dihydrate from Sigma Aldrich. Sodium Chloride 0.9% for Irrigation (AHF7123) was purchased from Baxter and used to dilute the above agonists, as well as setting the light transmission aggregometer baseline prior to platelet testing. All other chemicals, unless specified otherwise, were procured from Sigma Aldrich.

Section 1.2 General Human Platelet Washing Protocol

Human ethics protocol 2014/244, approved by the University of Sydney, permitted blood collection from consenting donors. For aggregation experiments, platelets collected from healthy adults (aged 18 - 60 and who had not taken antiplatelet medication in the prior 2 weeks) was immediately anticoagulated with acid citrate dextrose (ACD) (12.1 mM sodium citrate, 10.4 mM citric acid, 15.7 mM D-glucose, and 10 mM theophylline), and supplemented with 20 U/mL enoxaparin sodium (Clexane) and 0.005 U/mL apyrase (A6535-500UM, Sigma Aldrich). This was followed by a rest period at 37 °C for 15 minutes. Blood was then spun at 200 x *g* for 10 minutes. Platelet-rich plasma (PRP) was retained and rested for 10 minutes, before being centrifuged at 1700 x *g* for 5 minutes. Platelet-poor plasma (PPP) was removed, and pelleted platelets resuspended in an equal volume of platelet washing buffer (PWB) (4.3 mM K_2HPO_4, 4.3 mM Na_2HPO_4, 24.3 mM Na_3PO_4, 113 mM NaCl, 5.5 mM D-glucose, 10 mM theophylline, and 0.5% BSA, pH 6.5) containing 20 U/ml enoxaparin sodium (Clexane) and 0.01 U/mL apyrase. The resuspended platelets were rested at 37 °C for 10 minutes before being centrifuged at 1500 x *g* for 5 minutes. The PWB was removed and pelleted platelets were resuspended in Tyrode's buffer (12 mM $NaHCO_3$, 10 mM HEPES, 137 mM NaCl, 2.7 mM KCl, 5.5 mM D-glucose, 5 mg/mL BSA, 1 mM $CaCl_2$, pH 7.3) with the addition of 0.02 U/mL apyrase. The final platelet concentration was approximately 3 × 10^8 cells/mL. Platelets in Tyrode's buffer were allowed to rest for 30 minutes prior to the commencement of experiments.

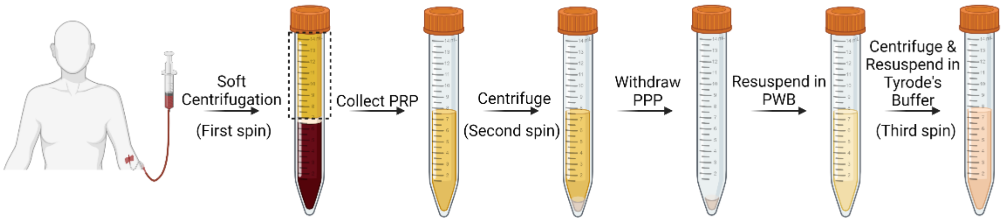

Figure S1. Schematic platelet washing workflow.

Section 1.3 General Procedure for Platelet Lysis and Lysate Collection

Platelets were lysed in a buffer composed of 50 mM HEPES, 150 mM NaCl, 1% Triton-X, 1 mM tris-(2-carboxyethyl)phosphine (TCEP), and 1x cOmplete™ EDTA-free Protease Inhibitor Cocktail tablet (COEDTAF-RO, Roche Diagnostics) at pH 7.4. Following a 10-minute centrifugation at 13,000 *x g* at 4 ºC, the supernatant was collected. The protein concentration was determined using the Bradford assay (23200, ThermoFisher Scientific) relative to BSA as the reference standard.

Section 1.4 General Procedure for SDS-PAGE

10% SDS-PAGE gels were prepared according to the Bio-Rad protocol. The protein samples, once prepared, were denatured using 4x Laemmli Sample Buffer (1610747, Bio-Rad) combined with 6 mM TCEP. These were then loaded into gel wells, one of which included 4 μL of Precision Plus Protein Dual Color Standards (1610374, Bio-Rad). Electrophoresis was carried out at 120 V until completion.

Section 1.5 General Procedure for In Vitro CuAAC Conjugation

Platelet lysates or protein solutions were treated with activity-based probes. Protein concentrations were determined using either Bradford assay or using the Nanodrop 2000c Spectrophotometer (ThermoFisher Scientific). CuAAC conjugation was performed with final concentrations of 5% t-BuOH, 1% SDS, 1 mM $CuSO_4$, 0.1 mM Cu(II)-TBTA (21050, Lumiprobe), 2 mM sodium ascorbate, 5 μM Cy5-azide (777323, Sigma Aldrich) or 200 μM biotin-PEG3-azide (D3730, Lumiprobe). Reactions were conducted at 37 °C for 30 minutes.

Section 1.6 General Procedure for In-Gel Protein Digestion and Peptide Extraction

Proteins from SDS-PAGE were first imaged with either QC colloidal Coomassie (161-0803, Bio-Rad) or SYPRO Orange (S6651, ThermoFisher Scientific), and then excised using a sterile scalpel blade and cut into 1 mm cubes. Gel pieces were destained using 12 mM ammonium bicarbonate in 40% acetonitrile with rotation. Proteins in the gel pieces were reduced with 5 mM dithiothreitol (DTT) in 25 mM ammonium bicarbonate followed by alkylation with 10 mM iodoacetamide in 25 mM ammonium bicarbonate in the dark. Gel pieces were dried using 100% acetonitrile and then under vacuum. Trypsin (T7575, Sigma Aldrich), at a trypsin/protein ratio of 1:100, was added to the dried gel pieces and diluted with 25 mM ammonium bicarbonate, pH 8 – 8.5. In-gel digestion was allowed to proceed overnight at 37 °C, then stopped by adding a final concentration of 1% formic acid. Gel pieces were sonicated for 5 minutes, and the peptide solution was collected. To fully extract peptides, gel pieces were treated with 25 mM ammonium bicarbonate with 5% formic acid in 50% acetonitrile for 45 minutes, and 25 mM ammonium bicarbonate with 5% formic acid in 90% acetonitrile for 5 minutes; the solution was combined with the trypsin extraction. The peptide extraction was then dried under vacuum.

Section 1.7 General Procedure for Peptide Sample Clean-up (Desalting)

A ZipTip with 0.6 µL C_{18} resin (ZTC18S, Sigma Aldrich) was activated using the wetting/elution solution (50% acetonitrile, 0.1% formic acid) and then equilibrated with the equilibration/wash solution (5% acetonitrile, 0.1% formic acid). Peptides, dissolved in 10 uL of loading buffer (3% acetonitrile, 0.1% formic acid), were loaded onto the ZipTip and washed 6 times with the equilibration/wash solution, followed by elution using the wetting/elution solution. Finally, the desalted peptides were dried under vacuum.

Section 2 In Vitro Biological Examination

Section 2.1 Human Platelet Preparation Protocols for Assessing the Impact of Electrophilic Natural Products on Platelet Activity

All natural product and agonist incubation procedures were conducted at 37 °C. Three distinct natural product incubation approaches were used: direct addition, jump-dilution, and washout. In the direct addition approach, washed platelets in Tyrode's buffer were rested for 30 minutes, followed by a 5-minute incubation with natural products or DMSO as a vehicle control before activation with an agonist. For the jump-dilution and washout approaches, natural product incubation occurred when platelets were resuspended in PWB. In jump-dilution, after a 2-hour incubation with the natural product of interest (typically at 20 μM unless stated otherwise), platelets were centrifuged at 1500 *x g* for 5 minutes, followed by removal of the supernatant and then resuspended in Tyrode's buffer for activity assessment. In the washout method, platelets were first incubated for 120 minutes with the chosen natural product in PWB, typically at a concentration of 20 μM in PWB unless otherwise specified. Following the incubation, the platelets were centrifuged at 1500 *x g* for 5 minutes to form a pellet. The supernatant was then discarded, and the pellet was resuspended in fresh PWB. After a rest period of 10 minutes, the platelets were then centrifuged again at 1500 *x g* for 5 minutes to remove the PWB, and the platelets were resuspended in Tyrode's buffer in preparation for subsequent biological assays.

Throughout the experiments, the concentration of DMSO to which the platelet samples were exposed was maintained at or below 0.1%. This concentration was predetermined as a level that does not affect platelet activity. All platelets in Tyrode's buffer were allowed to rest for 30 minutes prior to the commencement of experiments. Unless otherwise specified, all data related to the antiplatelet activities of natural products illustrated in the heatmap representation in **Figure S4** were generated through the jump-dilution approach.

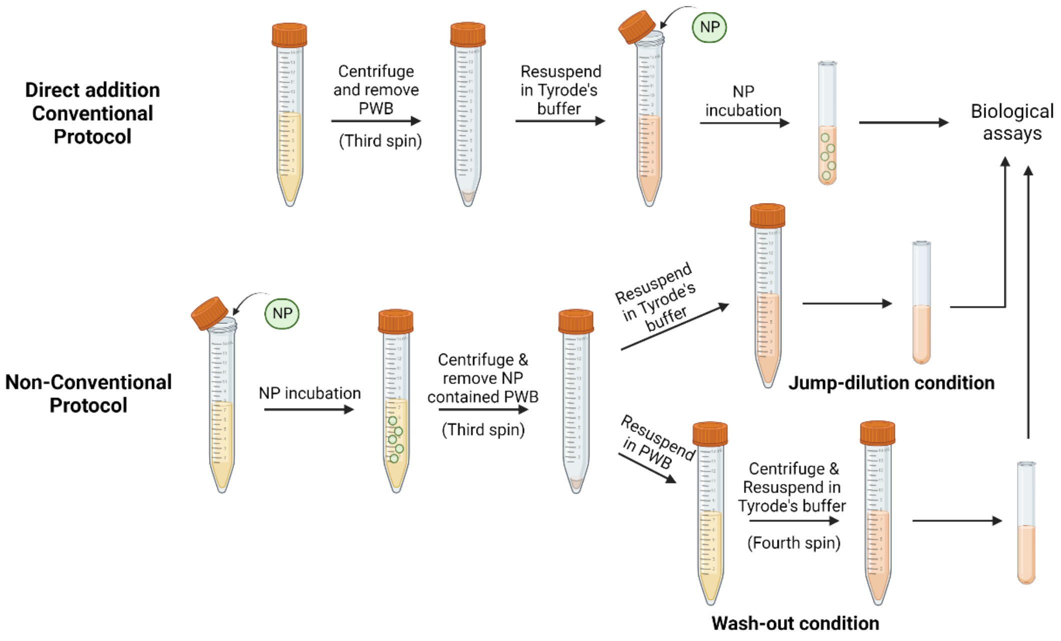

Figure S2. Schematic workflow of direct addition, jump-dilution and washout approaches for platelet natural product incubation.

Section 2.2 Platelet Aggregometry

Human platelet aggregation was measured via a 4-channel light transmission aggregometer (AggRAMTM, Helena Laboratories, VIC, AUS) employing the HemoRAM1.3 program. The turbidimetric measurement of clot formation was indicative of the percentage of aggregation over 10 minutes. Platelets were stirred at 800 rpm at 37 °C during measurement. Fibrinogen (0.5 mg/mL) was added prior to the start of measurement. Platelets were activated by adding 2–5 µM adenosine diphosphate (ADP), 0.5 µg/mL CRP, 0.05 U/mL or 0.1 U/mL Thrombin, or 0.2–0.5 µM thromboxane A2 receptor agonist (U46619) at 1 minute measurement. The concentration of ADP and U41699 used were donor-dependent, where titrations were conducted using vehicle-treated platelets to determine the optimal agonist concentration for platelet activity examination.

Relative platelet aggregation capacity (%) was presented as a percentage of the maximum aggregation of treatment platelets to the maximum aggregation of control platelets derived from the same donor (**Figure S3**).

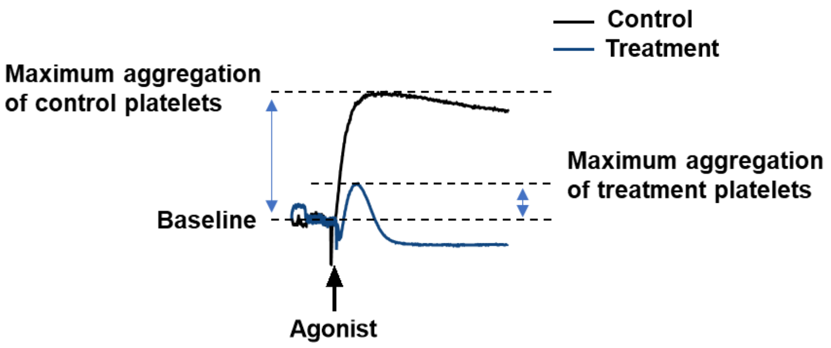

Figure S3. Illustration of relative platelet aggregation calculation from aggregometry traces obtained from analysis.

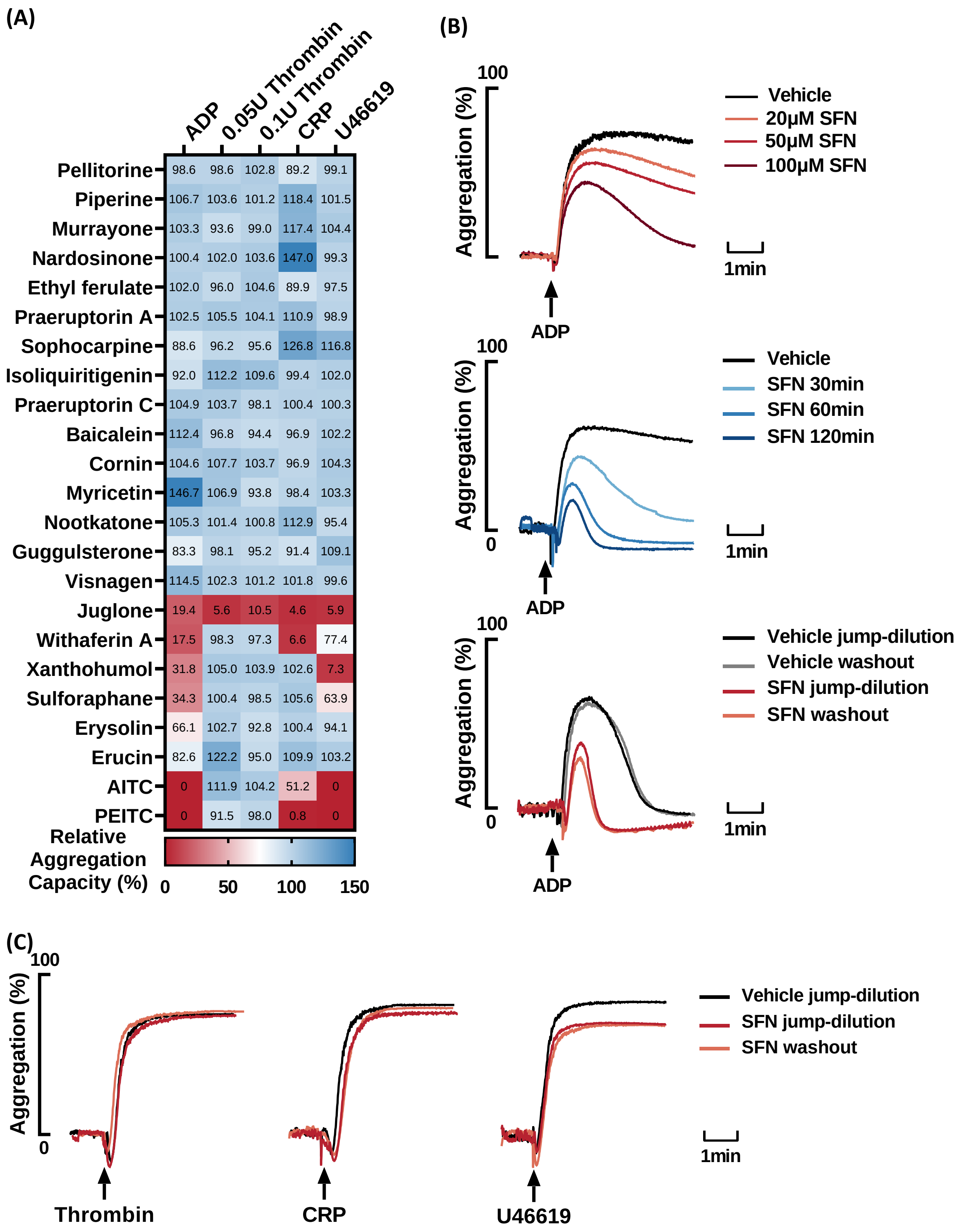

Figure S4. The antiplatelet activity of natural products tested on platelet aggregometry. **(A)** is replica of **Figure 2A** but showing the numerical quantification. It illustrates the relative aggregation activities of platelets following treatment with natural products through the jump-dilution protocol. ADP, thrombin, CRP and U46619 were selected in these experiments. The

term “Relative aggregation capacity (%)” denotes the maximum aggregation capacity of platelets treated with natural products in comparison to those treated with the vehicle control. Each entry represents the average taken from measurements performed on two distinct donors except the entries corresponding to SFN activities represent the average of 12 donors. **(B)** provides representative traces from ADP-activated platelet aggregometry experiments where platelets were exposed to different concentrations of SFN using a direct addition approach (upper graph), where platelets were treated with 20 μM SFN for various durations using a jump-dilution approach (middle graph) and where platelets were subjected to jump-dilution and washout conditions to compare the impacts of SFN (lower graph). **(C)** provides platelet aggregometry representative traces where platelets were subjected to jump-dilution and washout conditions for comparative analysis of the impacts on thrombin, CRP and U46619-mediated platelet aggregation.

Natural Product	Donor	Final Concentration (μM)	Relative Aggregation Capacity %			
			ADP	Thrombin	Collagen	U46619
Pellitorine (1)	Mouse	20	109*	57^	116*	118*
Piperine (2)	Rabbit	100	118*	110*	87^	93^
Murrayone	Human	20	133*	121*	130*	117*
Nardosinone	Human	20	100*	105*	112*	111*
Ethyl Ferulate	Human	100	108*	106*	105*	117*
Praeruptorin A (3)	Human	20	40*	127*	78*	4*
Sophocarpine	Human	20	115*	115*	121*	122*
Isoliquiritigenin	Human	19.5	102*	86*	37*	95*
Praeruptorin C	Human	20	75*	96*	87*	11*
Baicalein (4)	Rats	25	36^	61^	12^	107*
Cornin	Human	20	102*	118*	102*	106*
Myricetin (5)	Human	30 (†1mg/mL plant extract)	35†	58†	52^	171*
Nootkatone (6)	Rat	30	99*	90^	97^	95*
Guggulsterone (7)	Human	100	30^	117*	88*	99*
Visnagen	Human	20	120*	104*	87*	133*
Juglone (8)	Human	†1mg/mL plant extract	93†	16†	1†	1*
Withaferin A (9)	Human	5	144*	43^	40^	114*
Xanthohumol (10)	Human	3	105*	95^	5^	99^
Sulfurophane (11)	Human	100	40*	98*	18^	85^
Erysolin (12)	Human	†1mg/mL plant extract	36†	124*	84†	118*
Erucin (12)	Human	†1mg/mL plant extract	36†	124*	84†	91*
AITC (13)	Rat	30	92^	89^	91^	97^
PEITC	Human	20	103*	113*	119*	125*

Table S1. Antiplatelet activities of the natural products of choice following the direct addition method. Data collected and transformed from the light transmission aggregometry traces reported in the literature (^). Data generated in this study (*). Values reported from literature where platelets were treated with the corresponding plant extract containing the natural product (†). The antiplatelet activity of natural products was reported against four platelet aggregation agonists according to literature – ADP (2- 10 μM), thrombin (0.05 – 3 U/mL), CRP (0.5 – 10 μg/mL), U46619 (0.66 – 2 μM).

Section 2.3 Microslide Flow Assays and Volumetric Analysis

The setup was as follows [**Figure S5(A)**]: An intermediate microslide was placed on the platform of a differential interference contrast (DIC) microscope (63x water objective, Leica Microsystems DM IRB). From right to left, the blood reservoir was a 20 mL Terumo syringe with the plunger removed, connected to a 3-way stopcock. To the right of the stopcock, a 20 mL Terumo syringe was connected, and to the left, a Male Luer Lock Barb (Qosina Corp., NY, USA). The barb was attached to 50 cm silicone thin-wall tubing (0.8 mm diameter, Watson-Marlow, Falmouth, UK) that was in turn connected to the microslide. Similarly, from the left of the microslide, the following items were sequentially attached: 50 cm tubing, a Male Luer Lock Barb, a 3-way stopcock, and a 30 mL Terumo syringe (23.36 mm inner diameter) for withdrawal, which was secured to a syringe pump (PHD ULTRA™, Harvard Apparatus). A 20 mL Terumo syringe was connected to the final opening on the stopcock. Live observation was captured using the Leica DM IRB microscope (Leica Microsystems, Wetzlar, Germany), with an ORCA-Flash4.0 camera (Hamamatsu Photonics, Shizuoka, Japan) and the Micro-Manager-1.4.20 program. Videos and images were subsequently visualized using ImageJ (v1.53k, National Institutes of Health, Bethesda, MD, USA).

Intermediate glass microslides (0.2 x 2 mm diameter, 10cm; VitroTubes™ #3520) (Vitrocom, NJ, USA) were coated with fibrillar collagen (Type 1, 100 µg/mL) by capillary action and incubated for 2 hours at room temperature. To remove non-adherent collagen, microslides were flushed with Tyrode's buffer (12 mM $NaHCO_3$, 10 mM HEPES, 137 mM NaCl, 2.7 mM KCl, 5.5 mM D-glucose, pH=7.3, 37 °C) for 1 minute post-2 hour coating. Treated (vehicle/ SFN/SFNp) whole blood was then flowed through the setup from the reservoir for 2 minutes. Non-adherent cells were flushed with Tyrode's buffer successively for 2 minutes without introducing bubbles. All liquids were perfused through microslides via the syringe pump at a flow rate of 1.44 mL/min, replicating the arterial shear rate (1800 s^{-1}) for a 30 mL Terumo syringe. Microslides were removed from the tubing, perfused with 2% paraformaldehyde (PFA) via capillary action, and fixed for 1 hour.

(A)

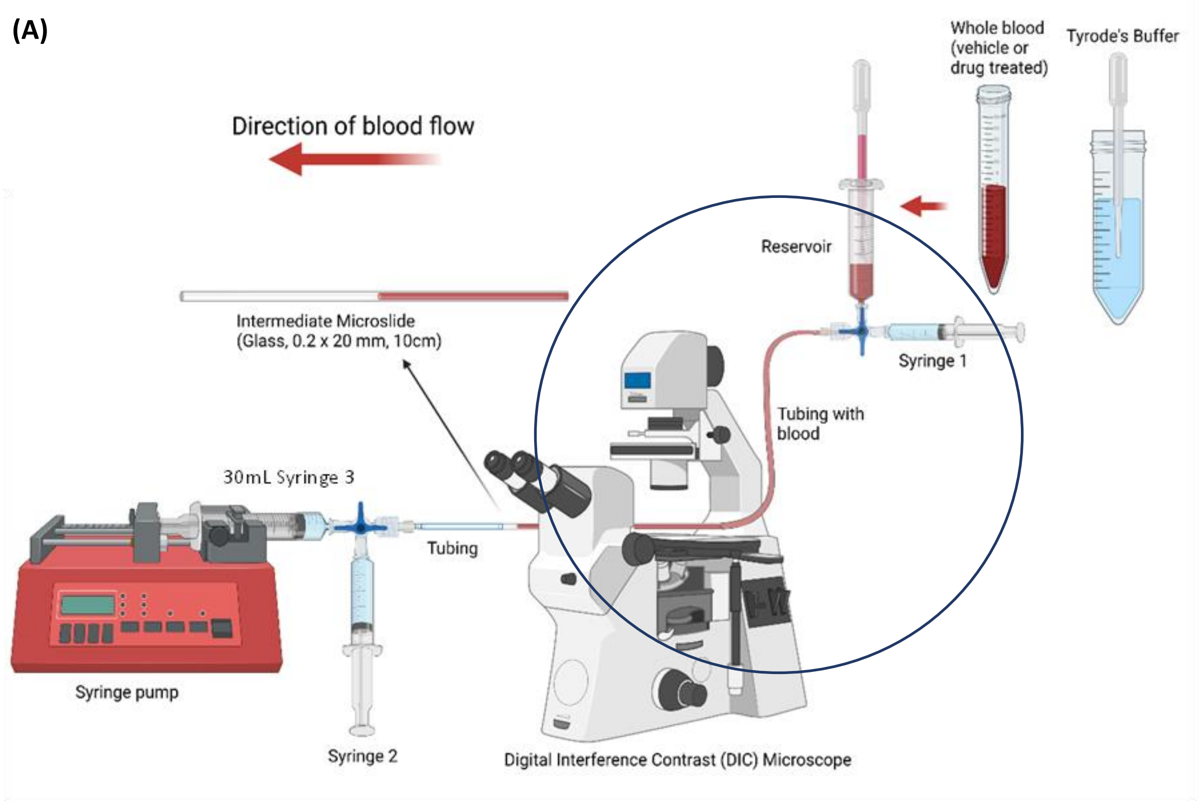

(B)

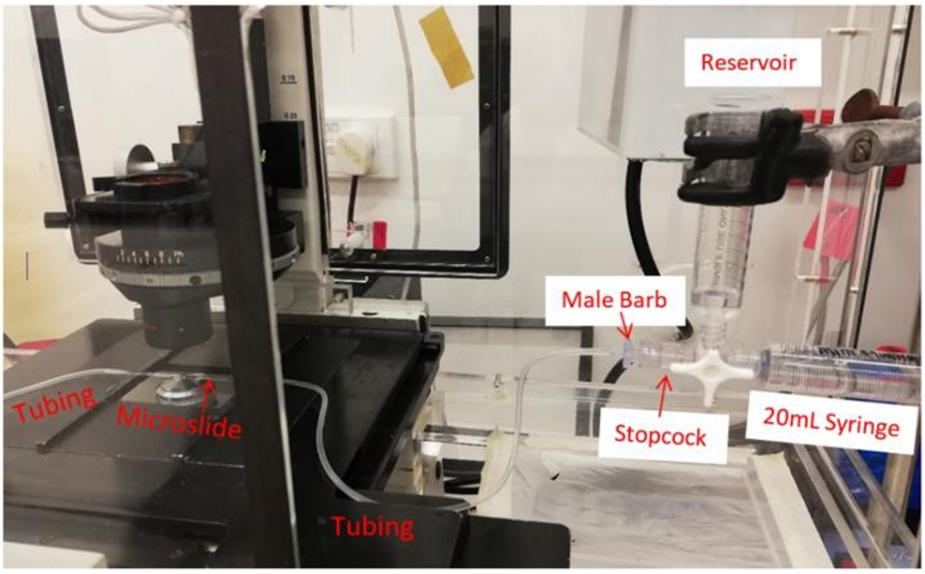

Figure S5. Setup for Microslide Flow Assay. **(A)** Schematic of microslide setup as described in the method. **(B)** Photographic image of the setup corresponding to the region circled in **(A)**.

PFA-fixed microslides were perfused with the green fluorescent lipophilic membrane dye DiOC6 (ThermoFisher Scientific, MA, USA) for 1 hour for fluorescent imaging and quantification of platelet thrombi volume via conjugation with fluorescein isothiocyanate (FITC).

Volumetric analysis was conducted using confocal microscopy (Nikon Eclipse Ti, 40x water objective) through the program NIS-Elements AR.5.21.03. Microslides were gently flushed with Tyrode's buffer immediately prior to imaging. 6 x 3 tiled z-stack images were taken in the middle, left, and right of each microslide, as seen in **Figure S6**. A corresponding DIC image was simultaneously taken for each capture. Quantification was completed using NIS Elements AR Analysis (5.21.03). Statistical significance was calculated using one-way ANOVA.

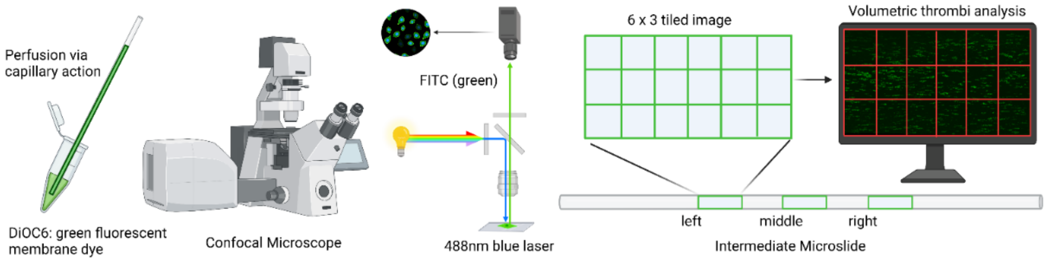

Figure S6. Workflow of Microslide Volumetric Acquisition. Microslides fixed for 1 hour with PFA were perfused with the platelet membrane dye DiOC6 (green) conjugated with fluorescein isothiocyanate (FITC). Three 3-dimensional 6 x 3 z-stack tiled images (left, middle, right) were acquired for each microslide, using a confocal microscope (40x, water objective).

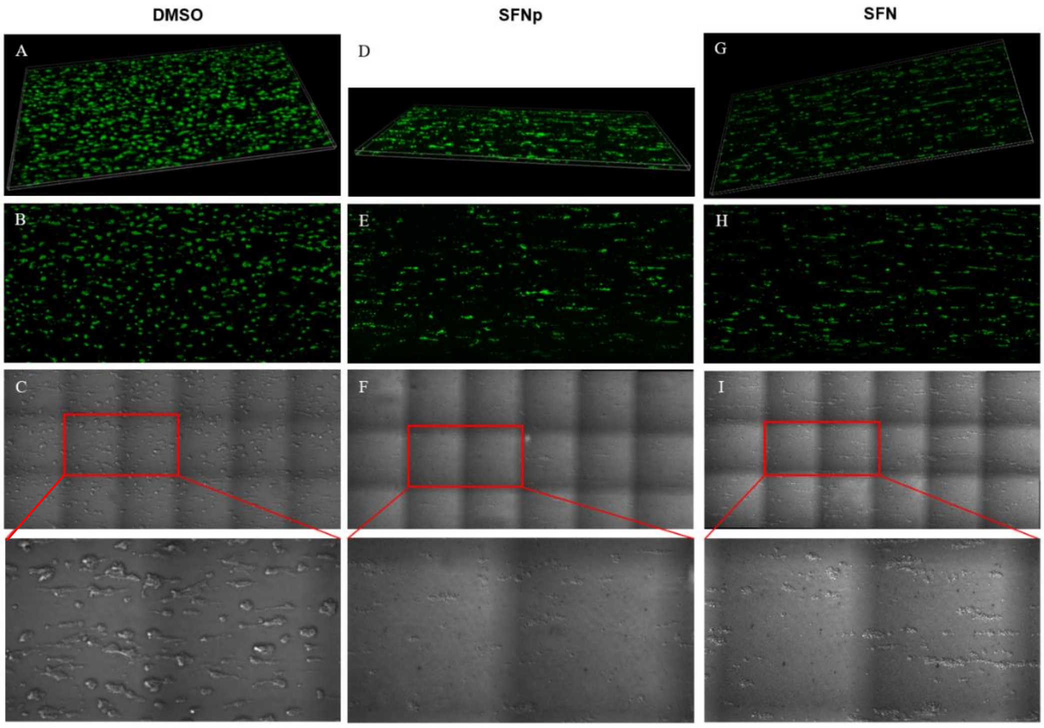

Figure S7. Images of PFA-fixed Thrombi on Microslides perfused with DiOC6. The following are representative fluorescent (A, B, D, E, G, H) and DIC images (C, F, I) of DMSO-treated thrombi (A, B, C), SFNp-treated (D, E, F), and SFN-treated (G, H, I) thrombi through the middle horizontal plane (40x, water objective). 3-dimensional models are shown in (A, D, G) whereas (B, C, E, F, H, I) are 2-dimensional captures. Boxed areas (red) in (C, F, I) are magnified for clearer distinction of thrombi difference.

Section 3. Using a SFN Alkyne Probe to Investigate the Targeted Proteome in Platelets.

Note: Refer to **Section 7** for the detailed chemical synthesis of the SFN alkyne probe (SFNp) and the nitrile analog probe (NCOp).

Section 3.1 Phenotypic Validation of SFNp and NCOp.

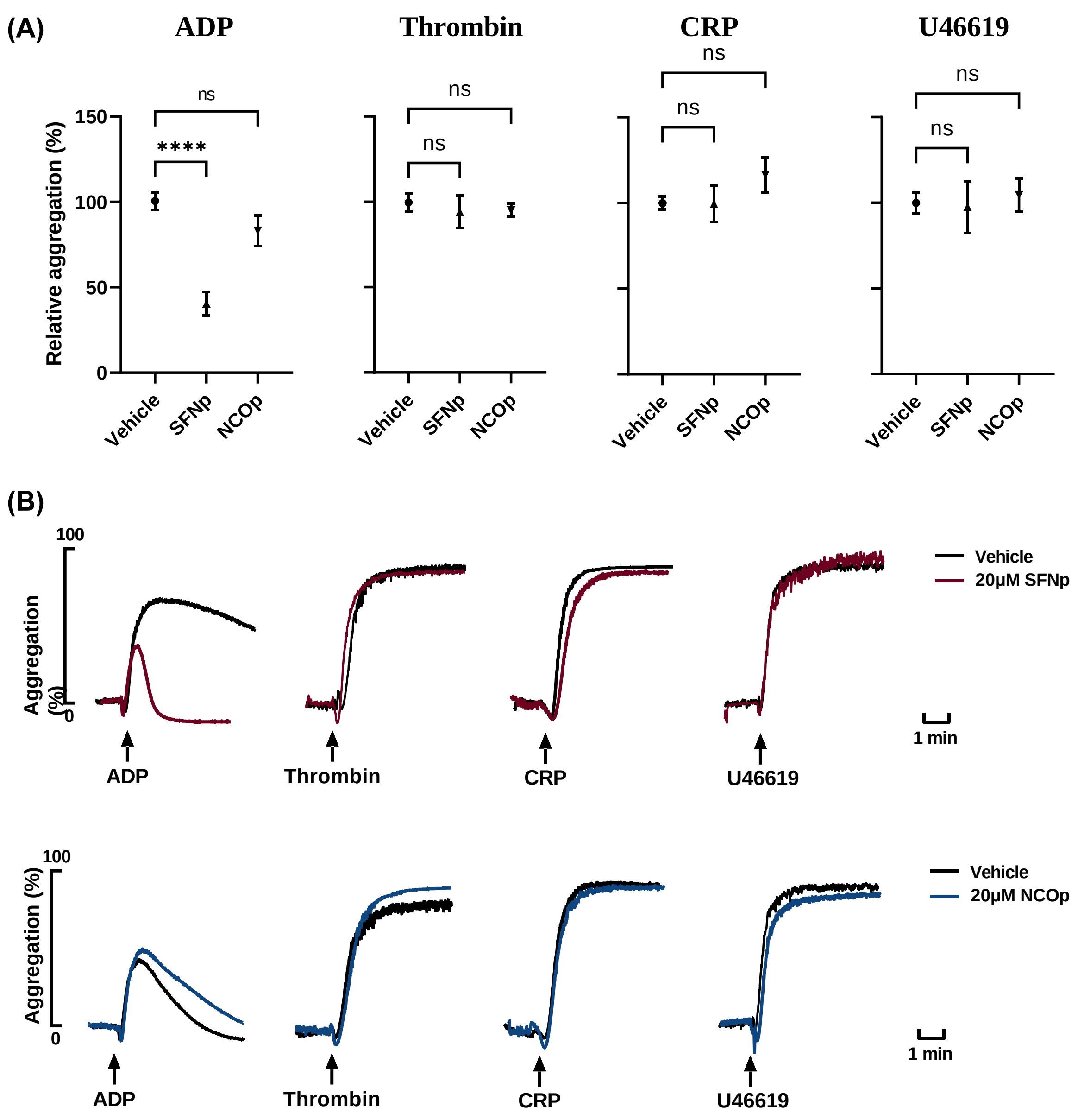

Figure S8. Comparative analysis of antiplatelet activities between vehicle, SFNp and NCOp. The platelet aggregometry experiments reveal that SFNp exhibits an ADP inhibition profile,

while NCOp exhibits negligible antiplatelet activities. **(A)** presents the quantitative analysis of the relative aggregation capacity of platelets subject to treatment with vehicle, SFNp or NCOp. The platelets were activated by ADP (n=8), thrombin (n=4), CRP (n=4) and U46619 (n=3). Relative aggregation capacities were depicted as percentages and are expressed as mean ± SEM. Statistical analysis was conducted using one-way ANOVA. **(B)** provides representative traces from the platelet aggregometry experiments for platelets treated with SFNp and NCOp.

Section 3.2 In-Gel Cy5 Fluorescence to Visualize Proteome Modified by SFNp

Washed platelets in PWB were incubated with 20 μM activity-based probes or vehicle control for 2 hours before cell lysis. Platelet lysates (1 mg/mL) were subjected to the Cy5 CuAAC conjugation conditions as outlined in the general method section. After 30 minutes, samples were quenched with 4x Laemmli Sample Buffer (1610747, Bio-Rad) with 6 mM TCEP and loaded into a 10-well gel. The proteome was resolved following a standard SDS-PAGE procedure. In-gel Cy5 fluorescence was visualized using the ChemiDoc Imaging System (Bio-Rad, CA, USA) and the proteome was stained with Coomassie Blue (1610436, Bio-Rad), the respective image of which was used as loading control (**Figure S9**).

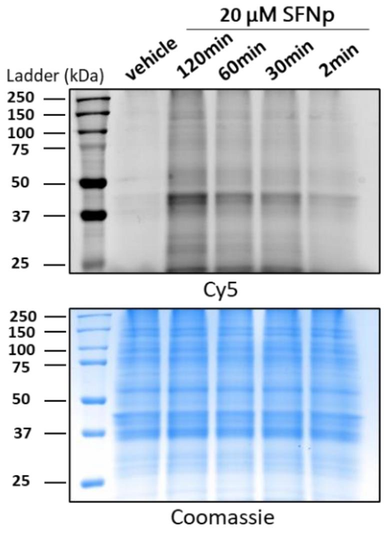

Figure S9. Kinetic analysis of SFNp covalent labeling of the platelet proteome. The in vitro Cy5 CuAAC conjugation was conducted with platelet lysates liberated from live platelets that

were pre-treated with 20 μM SFNp for various durations (2 minutes, 30 minutes, 1 hour and 2 hours, respectively). The labeling intensity is depicted as a function of incubation time, while the Coomassie-stained proteome serves as a loading control.

Section 3.3 Biotin-Streptavidin Mediated Protein Enrichment

Platelets were treated with an activity-based probe, either SFNp or NCOp, at a concentration of 20 μM, or with a vehicle (DMSO), in PWB for a duration of 2 hours. This is followed by cell lysis. CuAAC conjugation to biotin azide was subsequently conducted following the general procedure at a concentration of 10 mg/mL platelet lysate at 37 °C for 30 minutes before protein precipitation using MeOH/$CHCl_3$ (lysate/MeOH/$CHCl_3$, 4:4:1 v/v/v). Protein precipitates were washed twice with MeOH/$CHCl_3$ (1:1) and one time with acetone. Air-dried protein samples were resuspended in 50 mM HEPES and 150 mM NaCl with 0.5% SDS. Streptavidin enrichment was performed using Pierce™ High Capacity Streptavidin Agarose (20359, ThermoFisher Scientific) following the commercial protocol. The biotinylated proteome was eluted in 50 mM HEPES/150 mM NaCl (pH 7.4) with 50 mM biotin and 2% SDS. Proteins were separated on a standard SDS-PAGE gel and stained with SYPRO Orange (S6651, ThermoFisher Scientific) for visualization. The excised gel samples were then sent to Sydney Mass Spectrometry for protein identification.

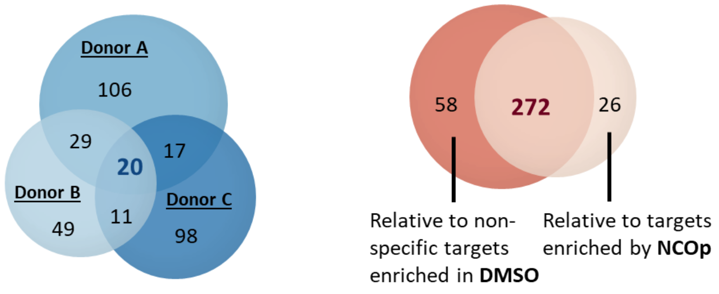

Figure S10. The Venn diagrams illustrate the protein targets enhanced by SFNp across three healthy donors through CuAAC-mediated biotinylation and streptavidin enrichment. The data is normalized against the vehicle (DMSO) sample (left diagram). The right diagram represents

the aggregate number of targets enriched from the three donors by SFNp, relative to either the vehicle-treated or NCOp-treated sample.

Section 3.4 Nanoflow LC-MS/MS

Peptides were resuspended in 10 μL of 3% (v/v) acetonitrile/0.1% (v/v) formic acid, and briefly sonicated. Samples were separated by nano-LC using an Ultimate 3000 HPLC and autosampler system (Thermo Fisher Scientific, Scoresby) coupled to an in-house fritless nano 75 μm × 40 cm column packed with ReproSil Pur 120 C18 stationary phase (1.9 μm, Dr, Maisch GmbH, Germany). LC mobile phase buffers were comprised of buffer A: 0.1% (v/v) formic acid and B: 80% (v/v) acetonitrile/0.1% (v/v) formic acid. Peptide elution employed a 5-40 % buffer B gradient for 21 minutes followed by 40-98 % buffer B gradient for 2 minutes at flow rate of 250 nL/min. The total acquisition time, including a pre-equilibrium, 95 % buffer B wash and re-equilibration, was 45 minutes. The LC was coupled to a QExactive Plus Orbitrap mass spectrometer (Thermo Fisher Scientific, Scoresby). Column voltage was 2300 V and the heated capillary set to 275 °C. Positive ions were generated by electrospray and the Orbitrap operated in data-dependent acquisition mode. A survey scan of 350 – 1550 m/z was acquired (resolution = 70,000, with an accumulation target value of 1,000,000 ions), and target value of 100,000 ions collected. Ions selected for MS/MS were dynamically excluded for 20 seconds.

The raw files generated were analyzed on Proteome Discoverer 2.5 (ThermoFisher, Waltham, MA, USA) using Mascot as a search engine against the human proteome database unless otherwise specified. Briefly, the workflow editor was used to create customized searches and result reports, where RAW data files were processed and converted to MSF (Magellan Server Files), then these files were subjected to a processing algorithm.

The search algorithm was set to a trypsin digestion, with a maximum of 2 missed cleavages, precursor mass tolerance was 10 ppm while the fragment tolerance was 0.1 Da. General variable dynamic modifications included N-terminus acetylation (mass shift of + 42.011 Da),

glutamine and asparagine side-chain deamidation (mass shift of + 0.984 Da) and methionine oxidation (mass shift of + 15.995 Da), modifications for specific study were indicated in the corresponding session. The raw data was processed accordingly and MSF files generated were directed to the consensus workflow. No delta mass filter was applied, and all peptide spectrum matches (PSMs) were grouped and validated. The automatic mode of validation was used for recovery of peptides where confidences were assigned to the PSMs and peptides based on the defined target FDRs. Finally, the proteins were grouped by applying strict parsimony principle and the output file was generated. All spectra presented in **Figure 4D** and **S11** have not been edited.

Section 3.5 Analysis of LC-MS/MS Proteomic Outputs

For data analysis for the biotin-streptavidin mediated protein enrichment, label-free quantification (LFQ) through mass spectrometry was conducted via Proteome Discoverer 2.5 (ThermoFisher, Waltham, MA, USA) to compare the abundance of protein precursor ions from the SFNp, NCOp and vehicle samples. Proteins with more than one unique peptides were considered sufficient confidence for protein identification. Abundance ratios (R) were given as the analysis output of LFQ, which a ratio above 4 was considered significant enrichment.

Section 4. Unravel the Covalent Interactions Between SFN and PDIA6

Section 4.1 Generation of PDIA6 Mutants

pET-15b plasmid containing PDIA6 gene sequence was used as a template for Polymerase chain reaction (PCR) reactions using Phusion® High-Fidelity PCR Master Mix with HF Buffer (New England Biolabs; NEB), where the corresponding designed primers were incorporated to generate the desired PDIA6 mutant plasmids (**Table S2**). The generated plasmids were then validated through Sanger sequencing serviced by Australian Genome Research Facility.

Primers	Forward (5’ to 3’)	Reverse (5’ to 3’)
**PDIA6 C291A**	gagctggtgctcctcagccgtcctcttggcaatg	cattgccaagaggacggctgaggagcaccagctc
**PDIA6 C297A**	agcacagccacaacagcgagctggtgctcctc	gaggagcaccagctcgctgttgtggctgtgct

Table S2. Site-directed mutagenesis primers for PDIA6 C291A and C297A mutants. Primers were designed using QuickChange Primer Design (Agilent) using PDIA6 sequence (NCBI Reference Sequence: XM_011510308.2).

Positive hits from Sanger sequencing were transformed into Origami 2 (DE3)pLysS competent cells and were grown using 2-YT Broth (Thermo Fisher Scientific) containing 100 μg/mL ampicillin (Thermo Fisher Scientific) at 37 °C and 110 rpm to an approximate optical density (OD) of 0.6-0.8. The protein expression was induced using 0.5 mM Isopropyl β-D-1-thiogalactopyranoside (IPTG) overnight at 20 °C and 110 rpm. The cells were harvested by centrifugation at 3400 x g at 4 °C for 20 minutes.

Cell pellets were resuspended in a resuspension buffer containing 50 mM Tris (pH7.5; Thermo Fisher Scientific), 400 mM sodium chloride (NaCl; Thermo Fisher Scientific) and 20 mM Imidazole (Thermo Fisher Scientific) which was supplemented with DNase I (Roche) and cOmplete™, EDTA-free Protease Inhibitor Cocktail (Roche) and lysed by sonication (60% amplification, five rounds of 10-second pulse on and 10-second pulse off) at 4 °C. Cellular

debris was removed by centrifugation at 17,000 x g for 30 minutes at 4 °C and filtered through a 0.45 µm membrane. The soluble supernatant was then loaded onto Äkta with HisTrap HP His tag protein purification columns (Cytiva) and purified with a gradual increase in imidazole concentration from 20 mM to 1 M. Imidazole was removed using a PD 10 desalting column (Cytiva). The eluted fraction was then further purified using Superdex® 200 Increase 10/300 GL (Cytiva). The proteins were validated by SDS-PAGE, and protein concentrations were estimated using a Nanodrop spectrophotometer.

Section 4.2 In-Gel Cy5 Fluorescence Analysis Showcasing the Kinetics of PDIA6 and PDIA6 Mutants Labeling with SFNp

PDIA6 (mutant) was reduced with 10 mM DTT for 30 minutes at room temperature, followed by desalting and buffer exchange using 7K MWCO Zeba spin desalting columns (89882, Thermo Scientific). The concentration of the resulting protein sample was determined by Nanodrop. 20 µM of protein in 50 mM HEPES and 150 mM NaCl (pH 7.4) was incubated with SFNp at 37 °C. At various time points, 1 µL of the mixture was collected and diluted in 20 µL pre-chilled dilution buffer (50 mM HEPES, 150 mM NaCl, 0.5% SDS, 50 µM GSH, pH 7.4). Standard in vitro CuAAC conjugation to Cy5-azide was conducted, followed by resolution of the protein sample on an SDS-PAGE gel. In-gel Cy5 fluorescence was visualized using the ChemiDoc Imaging System (Bio-Rad, CA, USA) and the proteome was stained with Coomassie Blue (1610436, Bio-Rad), the respective image of which was used as loading control. Four technical replicates were conducted. The kinetic labeling was found to reach the plateau at 30 minutes, as evident by Cy5 gel analysis [See **Figure 4(A)**] and ion chromatogram analysis of the SFNp-labeled b-domain peptides of PDIA6 C291A and C297A mutants at 30 and 60 minutes [**Refer to Figure S14**]. Cy5 labeling studies at longer time points (0, 10, 30, 60, and 120 min) refer to **Figure S11**.

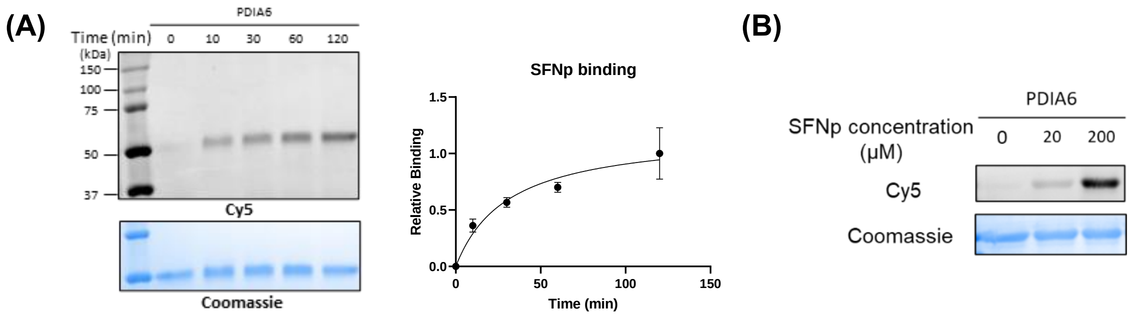

Figure S11. (**A**) Cy5 fluorescence analysis of PDIA6's covalent modification by SFNp at later time points. (**B**) Differential covalent modifications of PDIA6 by SFNp at concentrations of 20 and 200 μM over 60 minutes.

Section 4.3 Mapping PDIA6 Covalent Modification Sites

PDIA6 (mutant) was reduced with DTT and desalted as described before. Proteins were incubated with SFN (or SFNp) for 0, 30 and 60 minutes at 37°C followed by capping the remaining protein cysteines with 1 mM N-propargylmaleimide for 15 minutes and 1.1 mM DTT for 10 minutes to quench any unreacted small molecules. Small molecules were removed using 7K MWCO Zeba spin desalting columns (89882, Thermo Scientific). Proteins were denatured at 85 °C for 10 minutes before trypsin digestion (T7575, Sigma Aldrich) at a 1:50 (trypsin/protein) ratio, which was carried out overnight at 37 °C. The resulting tryptic digests were desalted using a ZipTip with 0.6 µL C_{18} resin (ZTC18S, Sigma Aldrich). The peptides were eluted with elution buffer (80 % acetonitrile, 0.1 % formic acid) and subsequently dried under vacuum.

For the PACMA31 labeling, PDIA6 was reduced and desalted using the previously described method. The proteins were then incubated with or without PACMA31 for 1 hour at 37 °C. Following this, the remaining protein cysteines were capped by treating them with 1 mM iodoacetamide for 15 minutes in the dark, followed by treatment with 1.1 mM DTT for 10

minutes. The protein cleanup and trypsinization procedures were performed as mentioned above.

Dried peptides were resuspended in 10 µL of 3% (v/v) acetonitrile/0.1% (v/v) formic acid and submitted to nanoflow LC-MS/MS analysis following the general method described in **Section 3.4**. The peptide elution employed a 5-50 % buffer B gradient for 31 minutes followed by 50-98 % buffer B gradient for 25 minutes at flow rate of 250 nL/min. The total acquisition time, including a pre-equilibrium, 95 % buffer B wash and re-equilibration, was 90 minutes.

For PDIA6 modification site analysis, the raw files generated were analyzed on Proteome Discoverer 2.5 (ThermoFisher, Waltham, MA, USA) using Mascot as a search engine against the PDIA6 (mutant) sequences for SFN and PACMA31 modification site identification. (**Section 3.4**) Label-free quantification (LFQ) through mass spectrometry was conducted for the kinetic labeling experiments of PDIA6 wild-type and mutants for SFNp modification site identification. Additional variable dynamic modifications included N-propargylmaleimide (mass shift of + 135.0320 Da), SFN (mass shift of + 177.2876 Da), SFNp (mass shift of + 215.3356 Da), carbamidomethyl (mass shift of + 57.0214 Da) and PACMA31 (mass shift of + 430.4742 Da).

(A)

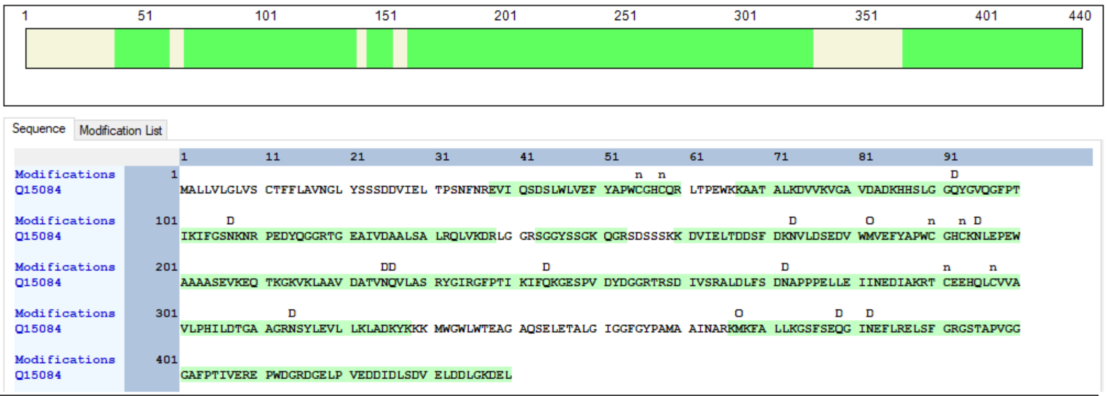

(B)

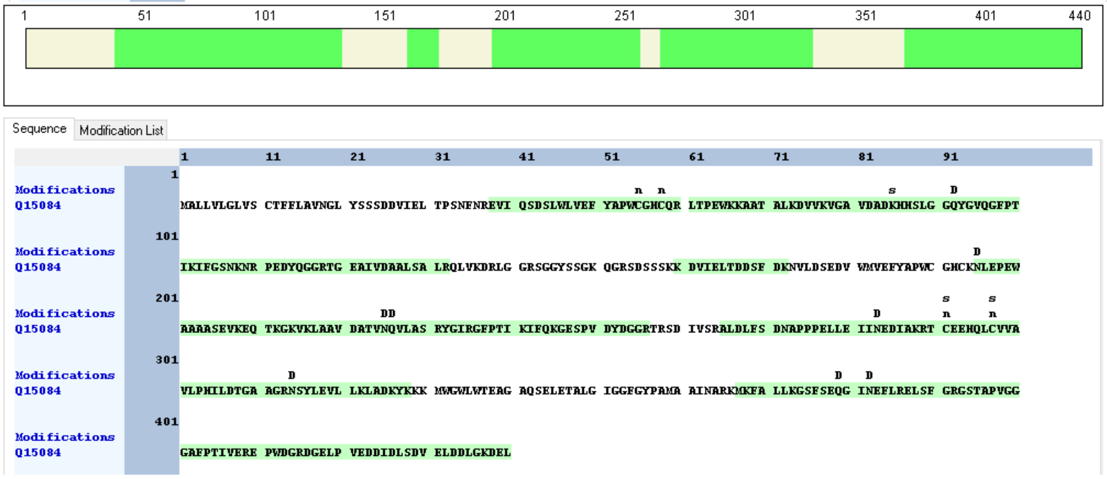

(C)

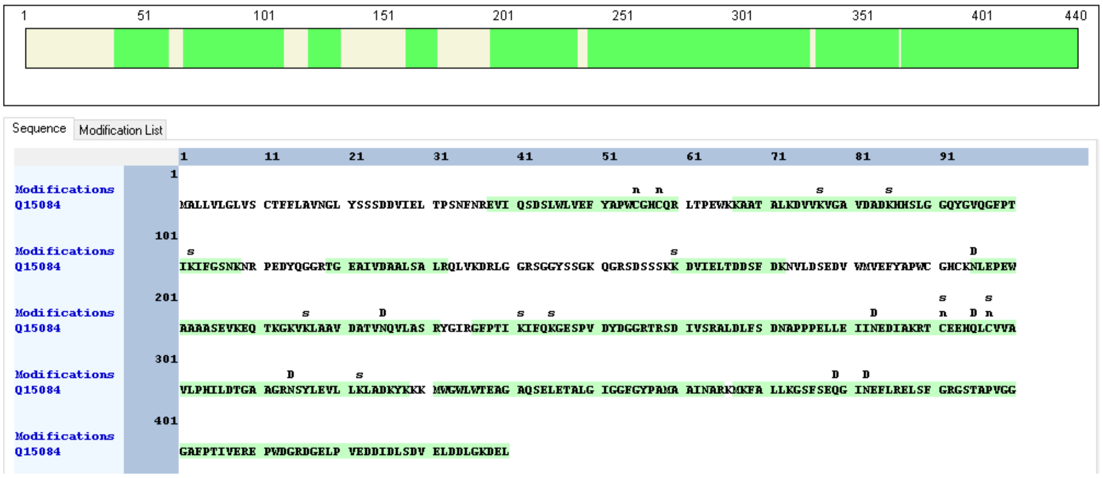

(D)

Amino acid (AA)	*No. of AAs in PDIA6*	*Percentage of SFN-labelled AA*
Cysteine	7	28.6%
Lysine	29	3.4%
Histidine	9	0%

Figure S12. Protein sequence coverage and SFN modification site in PDIA6 were analyzed through Proteome Discoverer 2.5. The panels present the analysis output following treatment with N-propargylmaleimide alone (**A**), 1.1 equivalents of SFN treatment for 1 hour followed by

N-propargylmaleimide capping (**B**), and 10 equivalents of SFN for 1 hour followed by N-propargylmaleimide capping (**C**). Modifications were abbreviated, “n” refers to N-propargylmaleimide, “s” refers to sulforaphane, “D” refers to deaminated and “O” refers to oxidation. (**D**) Table of relative labeling efficiency of SFN on Cys, Lys and His within PDIA6.

(A)

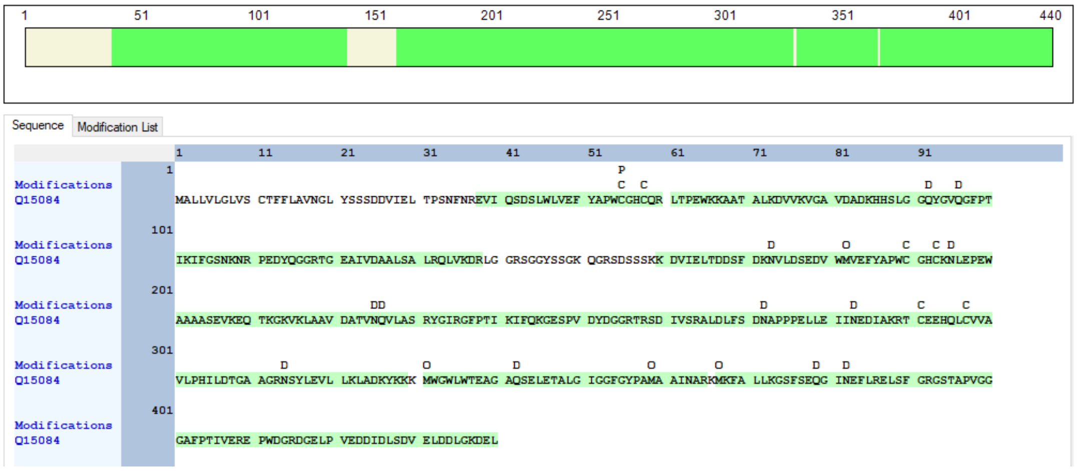

(B)

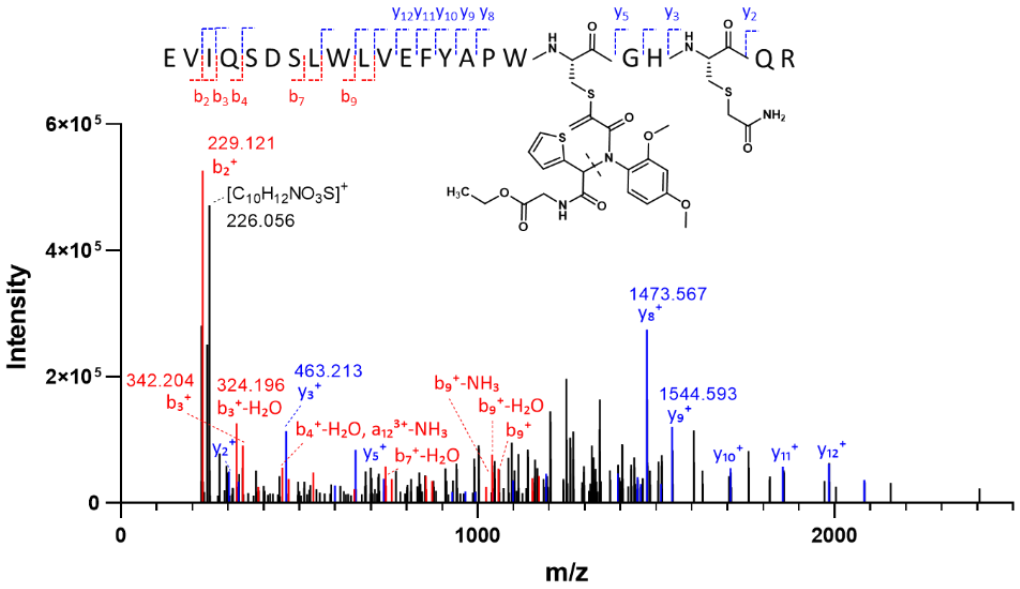

Figure S13. PACMA31 labeling of PDIA6 analyzed using Proteome Discoverer 2.5. **(A)** depicts the protein sequence coverage and PACMA modification site in PDIA6. Reduced PDIA6 was incubated with 10 equivalents of PACMA31 for 1 hour followed by a 15-minute incubation with 1 mM iodoacetamide in the dark. Modifications were abbreviated, “C” refers to carbamidomethyl, “P” refers to PACMA31, “D” refers to deaminated and “O” refers to oxidation.

(B) illustrates the fragmentation spectrum of PDIA6 [38-60] with a PACMA31 modification at Cys55.

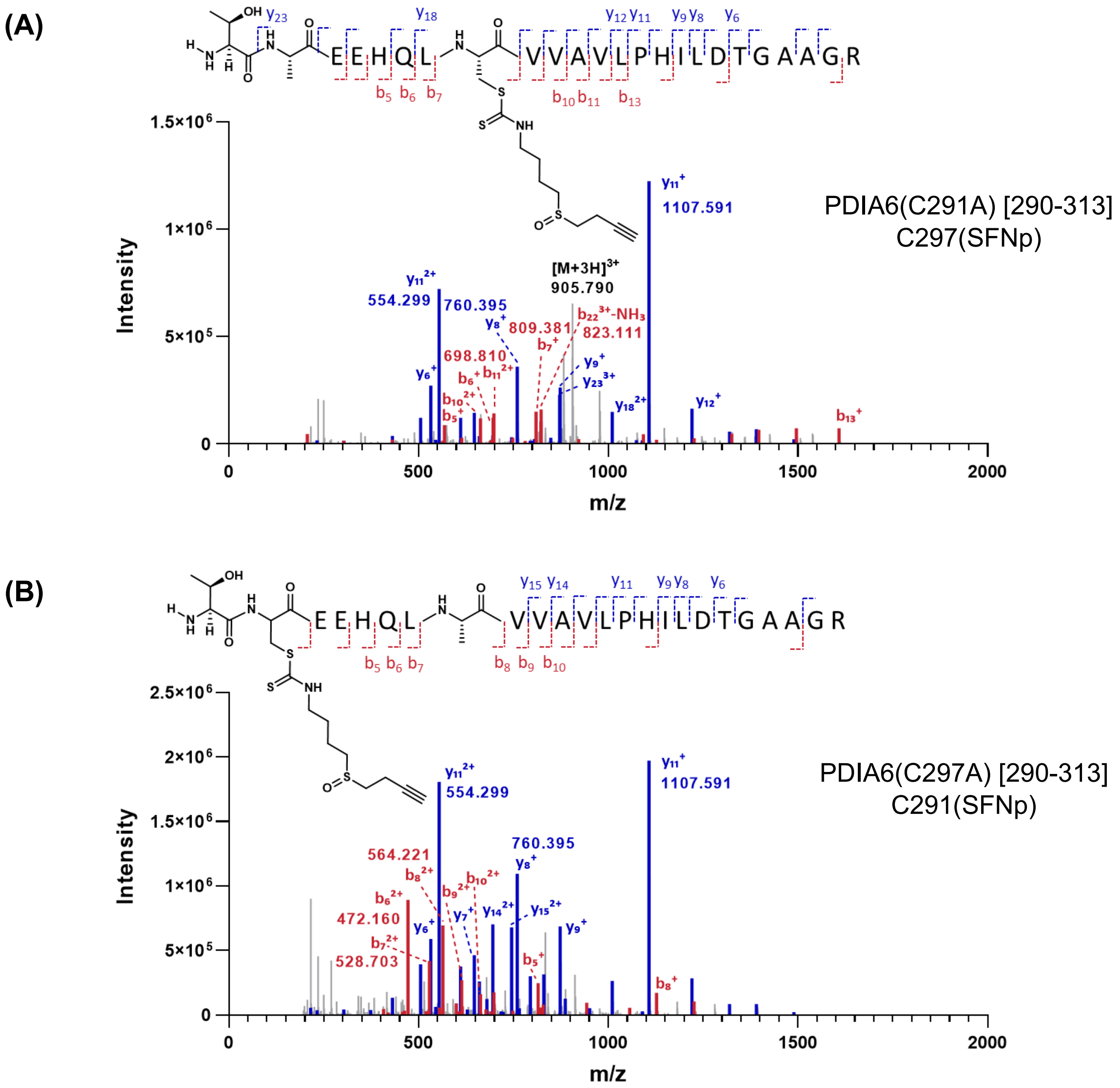

(C)

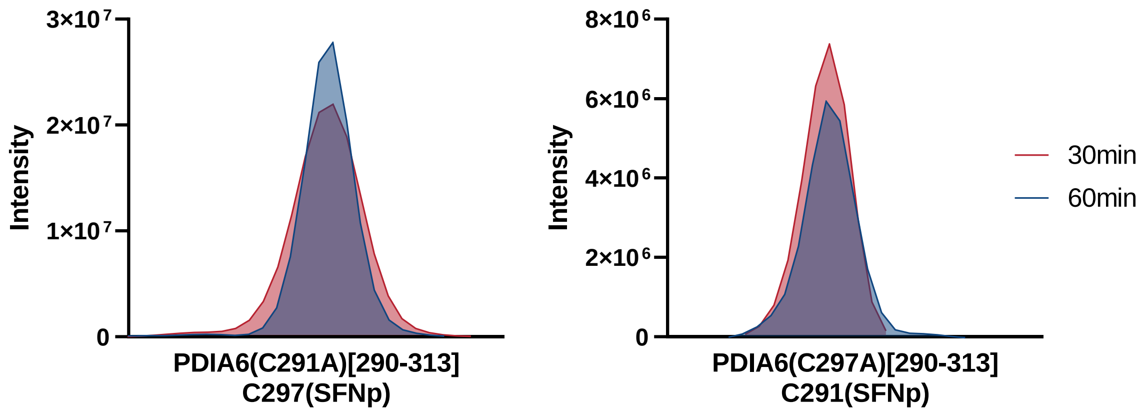

Figure S14. SFNp labeling of PDIA6 mutants was analyzed using Proteome Discoverer 2.5. (**A**) illustrates the fragmentation spectrum of PDIA6(C291A)[290-313] with a SFNp modification at Cys297. (**B**) illustrates the fragmentation spectrum of PDIA6(C297A)[290-313] with a SFNp modification at Cys291. (**C**) Extracted ion chromatograms of the SFNp-labeled peptide 290-313 of PDIA6 C291A and PDIA6 C297A mutants, at 30 and 60 minutes, from LFQ mass spectrometry analysis.

Section 4.4 Computational Molecular Modeling of PDIA6

The workflow that led to the identification of the binding pocket and binding conformations for SFN on PDIA6 is outlined in **Figure S15**. AlphaFold model (14) for full-length human PDIA6 (AF-Q15084-F1) was retrieved from the UniProt database (15) (accession code Q15084). The coordinates for residues 154 to 410 that constitute domains **a'-b** of PDIA6 [PDIA6 (154-410)] were extracted for use in this molecular modeling study. The structural model PDIA6 (154-410) was first prepared for modeling using Protein Preparation Wizard (16) from Schrödinger Suite (Schrödinger Release 2022-3), during which the disulfide bond between Cys297 and Cys291 present in the original AlphaFold model was reduced. Hydrogen bonding assignment was optimized using PROPKA for pH 7.4, and the structure was minimized converging heavy atoms to RMSD of 0.30 Å.

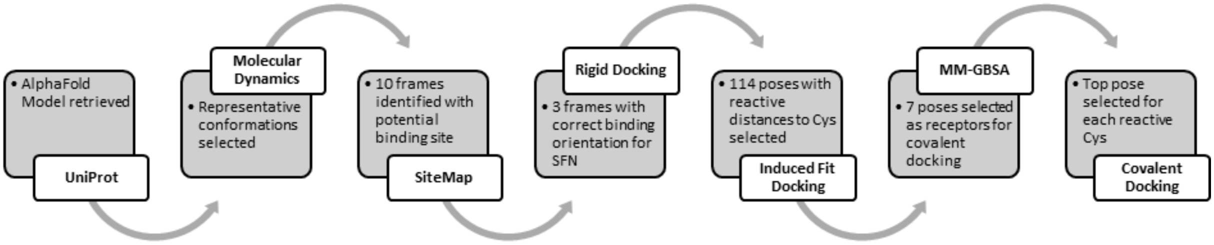

Figure S15. General workflow to identify the binding pocket for SFN on PDIA6 and predict the conformations of SFN reacting with either Cys291 or Cys297 on PDIA6.

As the AlphaFold model did not contain any binding pocket near the two cysteine residues of interest (Cys291 and Cys297), a molecular dynamics (MD) simulation was conducted to explore the conformational space and identify any potential binding pockets in the **b**-domain. The system for MD simulation was built using the OPLS4 force field. The prepared structure of PDIA6 (154-410) was placed in the center of an orthorhombic box filled with explicit TIP3P water molecules, and the size of the box was defined by having 10 Å of buffer region in all x, y, and z directions. The protein molecule was rotated to minimize the box volume. The system was then neutralized by adding eight Na^+ ions. The MD simulation was conducted using Desmond (17), running at a constant temperature of 310 K and constant pressure of 1.01325 bar. The system temperature and pressure were modeled using Langevin thermostat and barostat. Coulombic interactions were cut off beyond 9.0 Å. The system was first relaxed using the default relaxation process for the NPT ensemble in Desmond before MD production run of 400 ns. Trajectory frames were collected at 0.1 ns interval.

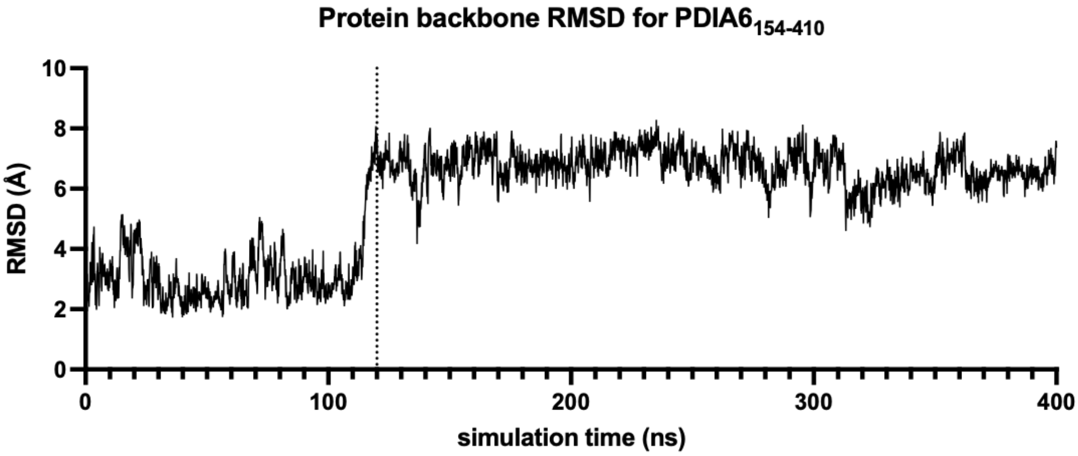

Figure S16. Protein backbone RMSD values for PDIA6(154-410) during MD simulations.

The MD simulation for PDIA6(154-410) equilibrated after 120 ns into the production run (**Figure S16**), therefore, trajectory frames between 120 ns to 400 ns of the MD simulation were analyzed further. Conformational clustering analysis was conducted for the region of interest (residues 288-293, 398-406 and 364-368) based on the backbone RMSD differences between the frames. A total of 27 representative conformations were identified. SiteMap (18, 19) (Schrödinger Release 2022-3) was used to analyze the 27 representative conformations and identified 10 top-ranked potential receptor binding site conformations near the two cysteine residues (**Table S3**).

Table S3. Potential binding sites and scores reported by SiteMap.

**Representative conformation**	**Volume (Å^3)**	**Residues**
**13**	29.2	A:335;A:362;A:404;A:406
**1**	55.2	A:288;A:291;A:292;A:403;A:406
**4**	65.5	A:288;A:291;A:403;A:404;A:406
**9**	34.0	A:288;A:291;A:292;A:403;A:405
**15**	50.8	A:288;A:292;A:406
**17**	85.4	A:288;A:291;A:292;A:362;A:364;A:403;A:404;A:406
**19**	93.0	A:288;A:292;A:362;A:403;A:404;A:405;A:406
**25**	79.6	A:288;A:291;A:292;A:345;A:364;A:365;A:403;A:404;A:405;A:406
**26**	89.2	A:288;A:291;A:292;A:345;A:362;A:364;A:403;A:404;A:405;A:406
**8**	52.8	A:288;A:291;A:292;A:364

Rigid receptor ligand docking calculations were then conducted to assess which of the 10 identified binding site conformations could accommodate SFN. Firstly, receptor grids were generated for all of the ten identified binding site conformations. The center of each grid was defined as the centroid of the residues lining the binding sites as identified by SiteMap (**Table S3**). The size of the grid was defined to allow docking of ligands with lengths smaller or equal to 20 Å. Rotatable groups were allowed for S-H group on Cys297 and Cys291. The structure of SFN was built and prepared using Maestro and LigPrep (Schrödinger Release 2022-3), respectively. The prepared structures of SFN were then modeled into the 10 receptor conformations using Glide (20-22) (Schrödinger Release 2022-3).

Examination of the predicted binding poses of SFN in the ten receptor grids showed that the binding site conformations from frames 19, 25 and 4 accommodated SFN in the correct binding orientation, i.e. with the isothiocyanate C atom of SFN in proximity to the S atom in Cys291 or Cys297 (**Figure S17**).

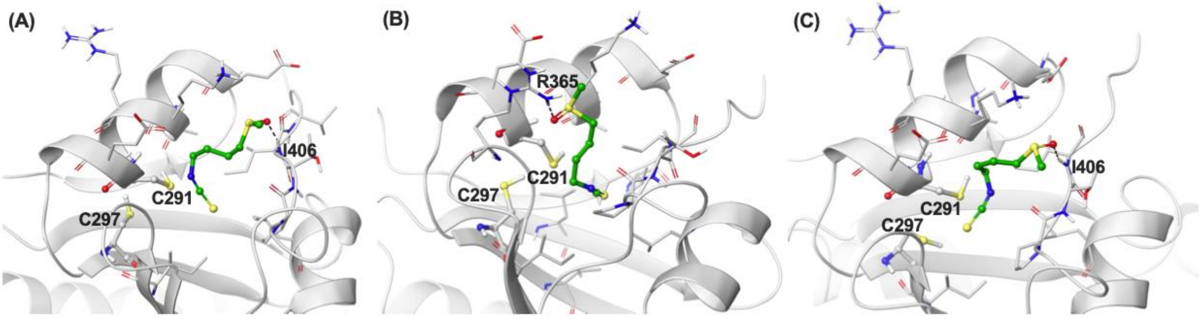

Figure S17. Binding pose of SFN predicted by rigid receptor ligand docking calculations to binding site conformations in **(A)** frame 19, **(B)** frame 25 and **(C)** frame 4. Protein carbon atoms are shown in white and SFN carbon atoms are shown in green. Both Cys291 and Cys297 are labeled. Hydrogen bonds are displayed as black dashed lines and the residues contributing to hydrogen bonds with SFN are also labeled.

SFN binding site conformations were further refined by modeling SFN into the three frames (19, 25, and 4) using induced fit docking (IFD) calculations (23-25), as this type of calculation allows the movement of side chains to optimize center of the grid was defined as the centroid of residues lining the pocket as identified by SiteMap (**Table S3**). The size of each grid was defined by allowing docking of ligands with lengths shorter or equal to 20 Å. IFD extended sampling protocol was used.

Predicted SFN-PDIA6 complex conformations from IFD calculations were then examined and those with potentially "reactive" distances (less than 5 Å) between the isothiocyanate C atom of SFN and the S atom on Cys291 or Cys297 were evaluated using molecular mechanics with generalized Born and surface area (MM-GBSA) calculations in Prime (26). The SFN binding conformation was further minimized and the MM-GBSA binding affinity scores were used to rank the different binding poses for SFN. A total of 7 SFN-PDIA6 complex conformations were identified, which show short distances (less than 5 Å) between isothiocyanate C atom of SFN and S atoms of the two Cys residues, as well as favorable predicted binding affinities that are lower than -30 kcal/mol (**Figure S18**).

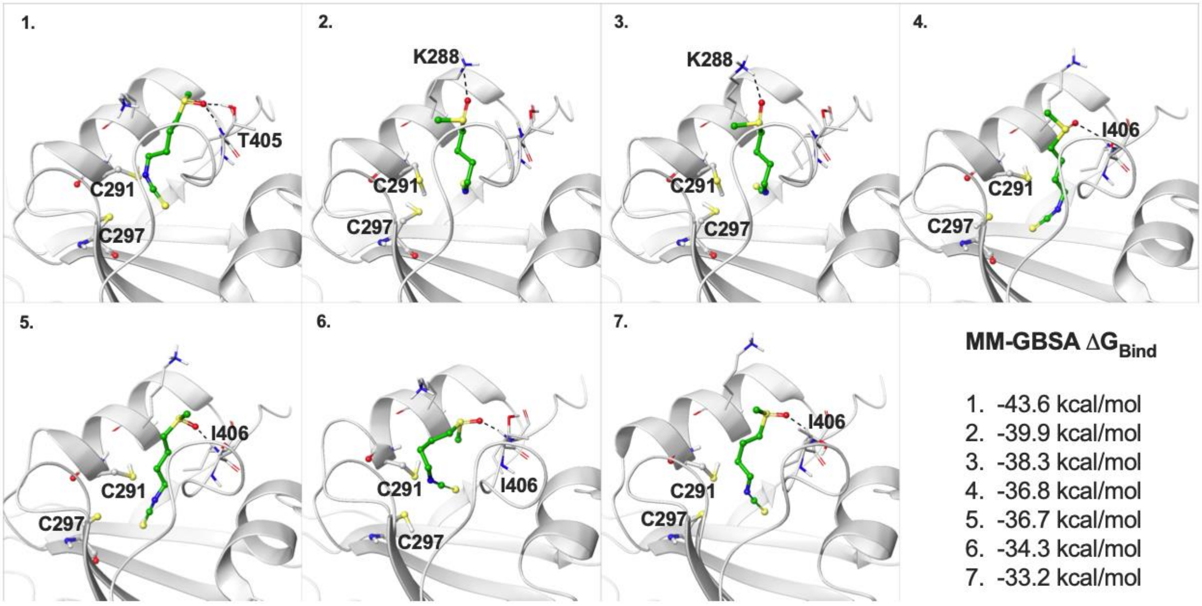

Figure S18. SFN-PDIA6 complex conformations selected after IDF and MMGBSA scoring. Protein carbon atoms are shown in white and SFN carbon atoms are shown in green. Both Cys291 and Cys297 are labeled. Hydrogen bonds are displayed as black dashed lines and the residues contributing to hydrogen bonds with SFN are also labeled.

Finally, we modeled SFN into all the 7 binding site conformations identified above using covalent docking calculations (27) to predict the conformation of SNF when it is covalently linked to either Cys291 or Cys297. For these covalent docking calculations, the reactive residue was selected as either Cys291 or Cys297. The centre of the enclosing receptor box was defined as the centroid of the corresponding SFN ligand already modeled in each receptor conformation from the docking calculations above, and the size of the box was defined to allow modeling of ligands similar in size to SFN. For this docking calculation, a custom reaction type had to be defined using keywords available in the covalent docking software (**Table S4**). The thorough Pose Prediction mode was used for the docking and MM-GBSA scoring was performed after docking. One best scored (best MMGBSA score) pose was selected for SFN linked to each Cys residue (**Figure S17**).

Table S4. Values for corresponding keywords to set up custom reaction type for covalent docking of SFN to PDIA6.

<table>
<tr><th>Keywords</th><th>Value</th></tr>
<tr><td>LIGAND_SMARTS_PATTERN</td><td>2, [S]=[C]=[N]</td></tr>
<tr><td>RECEPTOR_SMARTS_PATTERN</td><td>2, [C]-[S;H1,-1]</td></tr>
<tr><td>CUSTOM_CHEMISTRY</td><td>(“<1>|<2>”,(“bond”,1,(1,2)))</td></tr>
<tr><td>CUSTOM_CHEMISTRY</td><td>(“<2>=[N]”,(“bond”,1,(1,2)))</td></tr>
</table>

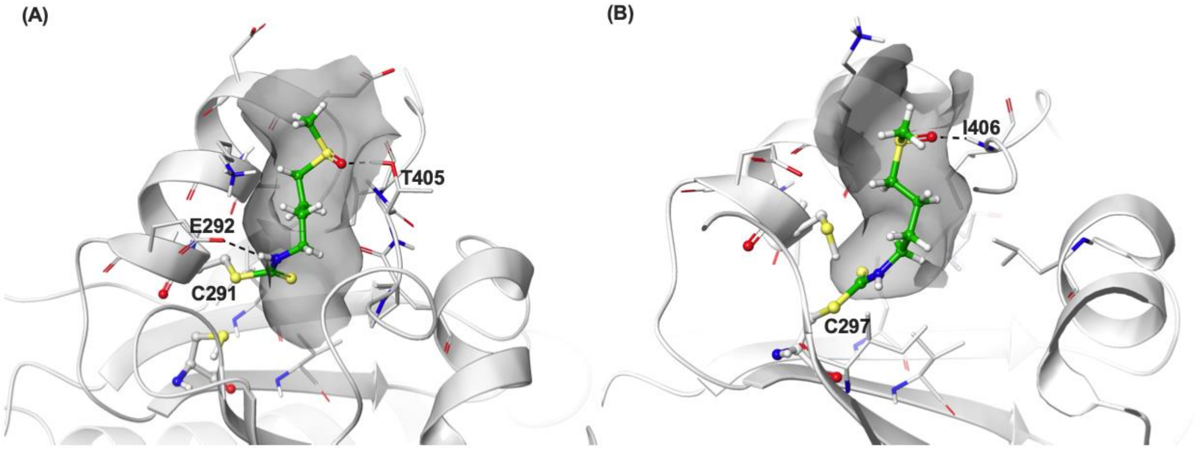

Figure S19. Top predicted conformation of SFN covalently linked to **(A)** Cys291 and **(B)** Cys297, obtained from covalent docking calculations. Protein carbon atoms are shown in white and SFN carbon atoms are shown in green. Cys291 and Cys297 are labeled. Hydrogen bonds are displayed as black dashed lines and the residues contributing to hydrogen bonds with SFN are also labeled.

Section 4.5 Insulin Turbidity Assay

PDIA6 (mutant) in its reduced state (5 μM) was preincubated with an array of SFN concentrations (10, 20, 80, and 200 μM) at 37 °C for a duration of 1 hour. Following this incubation, the mixture was promptly diluted with an assay buffer (180 μL) consisting of 0.1 mM potassium phosphate (pH 7.0), 2 mM EDTA, 0.13 mM bovine insulin, and 0.1 mM DTT, which resulted in a final PDIA6 concentration of 100 nM. This diluted mixture was then transferred to a 96-well plate. Turbidity measurements were performed at 650 nm utilizing CLARIOstar plate reader from BMG Labtech.

(A)

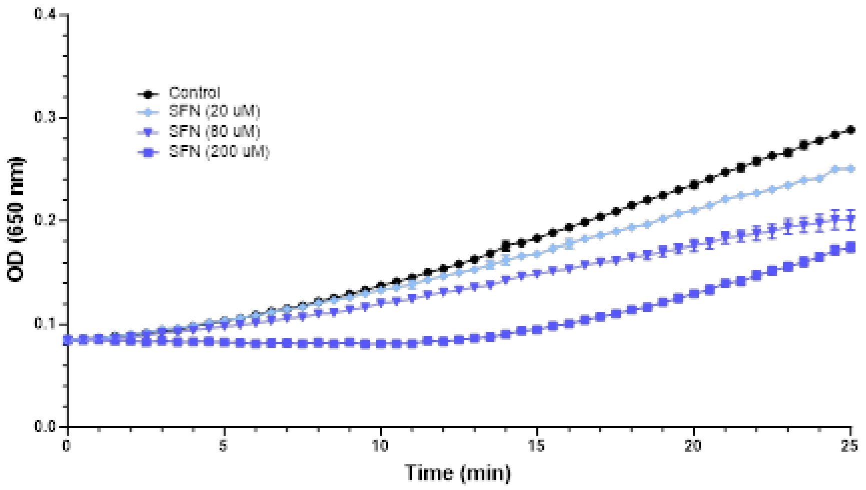

(B)

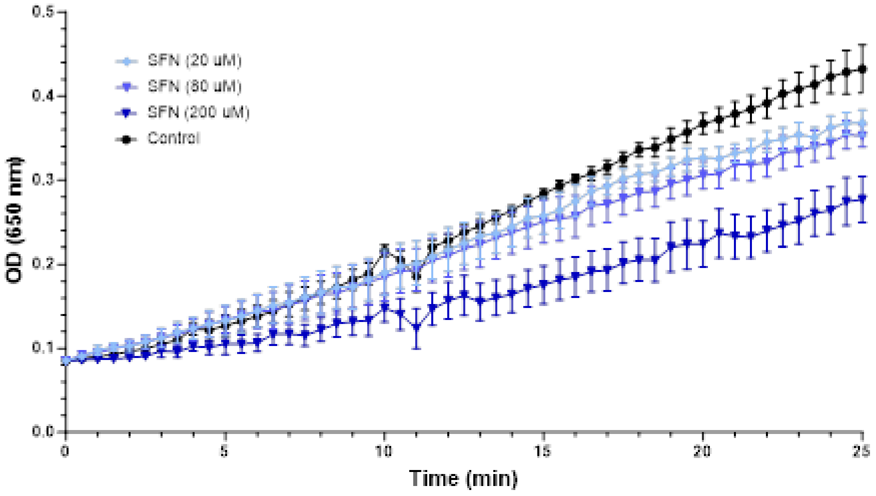

Figure S20. Insulin turbidity assay demonstrated the comparative impact on PDIA6 C297A (**A**) and C291A (**B**) mutant via covalent modulation by SFN.

Section 5. Uncovering the Interactome of PDIA6-SFN Covalent Complex through Co-Precipitation and Bioinformatic Approaches

Approximately 20 μg of His6-PDIA6 protein in 50 mM HEPES and 150 mM NaCl (pH 7.4, 490 μL), with or without prior treatment with SFN, was added to 50 μL of Ni-NTA agarose beads and incubated at room temperature for 1 hour. As a control, 50 μL of Ni-NTA agarose beads without the addition of PDIA6 was also prepared. Following the incubation, fresh platelet lysate (500 μg proteins at 1 mg/mL concentration) was subsequently added to each treatment beads and incubated for another hour at room temperature. Next, the beads were incubated with 500 μL of lysis buffer supplemented with 20 mM imidazole for 3 minutes at room temperature before centrifugation to remove the supernatant. This step was repeated once more using the same buffer. For the final wash, 50 mM HEPES buffer, supplemented with 300 mM NaCl and 20 mM imidazole, was added, and the mixture was incubated for another 3 minutes at room temperature before centrifugation and the removal of supernatant. The beads were resuspended in 40 μL of Laemmli buffer supplemented with 6% mercaptoethanol. The suspension was heated at 95 °C for 5 minutes, then centrifuged to collect the supernatants. A fresh buffer solution containing 50 mM HEPES, 300 mM NaCl, and 250 mM imidazole was subsequently added (20 μL), and the beads were centrifuged again to extract the supernatants. The respective supernatants were combined loaded onto a 10-well gel for SDS-PAGE analysis. The derived proteomes were stained using SYPRO Orange (S6651, ThermoFisher Scientific), and each individual sample lane was carefully excised and sectioned into multiple 1 mm cubes. These cubes were then subjected to in-gel trypsin digestion, followed by LC-MS/MS analysis to reveal the protein identities.

Section 5.1 Proteome Profiling and Ingenuity Pathway Analysis

Shotgun proteomic raw data were analyzed to generate a protein identification and label-free quantification (LFQ) report using the Sequest HT search engine in Thermo Proteome

Discoverer 2.5 against all reviewed human entries (canonical only) in Uniprot proteome database (downloaded 13/01/2023, with a total of 20594 entries). The hierarchical clustering analysis for heatmap visualization was conducted in TBtools (version v1.113) (28). The searching parameters for Proteome Discoverer and hierarchical clustering analysis were used as previously described in (29). For the purpose of comparison and data analysis, proteins with a coefficient of variation (%CV) ≤ 30% across replicates of the same sample type were included in further analysis to minimize the variance of LFQ analysis. The average protein abundance of every individual protein from the unmodified PDIA6 sample was calculated as the baseline. The average protein abundance of every individual protein influenced by the presence of SFN-modified PDIA6 was compared with this baseline. The proteins were considered as upregulated or downregulated based on following rules: proteins only expressed or disappeared in SFN treatment sample; proteins that increased or decreased by ≥ 0.5 (Z-scale) in SFN-modified PDIA6 compared to PDIA6. All upregulated or downregulated proteins in the SFN treatment sample were included and further analyzed using Ingenuity Pathway Analysis (IPA, version 90348151). The searching and statistic parameters for IPA were previously described in (30). Major protein signal transduction pathways underlying platelet aggregation and influenced by the SFN-modified PDIA6 were reconstructed based on IPA results and literature searching.

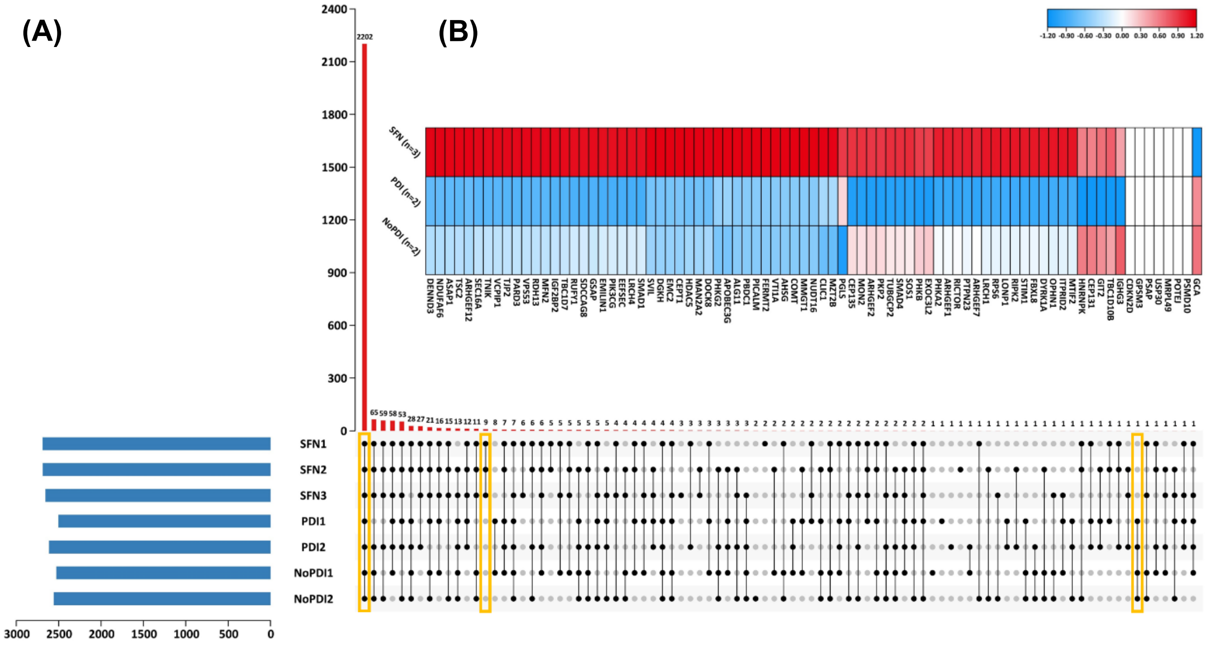

Figure S21. (A) Upset plot shows the distribution of all identified proteins across samples by shotgun proteomics, three clusters of proteins involved in further heatmap expression analysis were highlighted in orange **(B)** Heatmap shows the major protein changes in SFN/PDIA6.

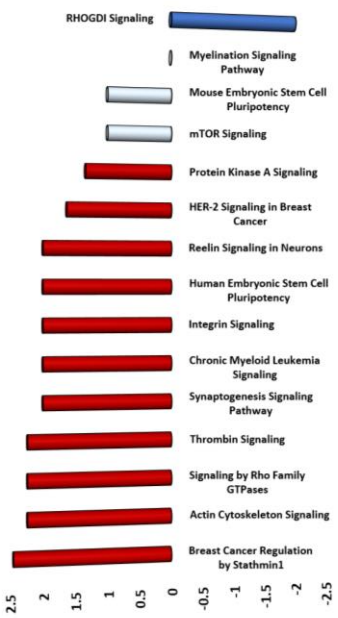

Figure S22. The pathway enrichment analysis was conducted in Ingenuity Pathway Analysis (IPA) software, and all significant pathways changed by SFN treatment to PDIA6 are displayed.

Section 5.2 Addition Flow Cytometry Assay to Support the Co-IP Analysis Results

Whole blood was treated with enoxaparin sodium (Clexane) and incubated with vehicle (DMSO) or 20 μM natural products (sulforaphane) for 20 minutes. Integrin activation was tested via fluorescence-activated cell sorting (FACS) with PAR-4 activating peptide (300 μM), PAR1 activating peptide (100 μM), and thrombin (0.1 U) via a BD AccuriTM C6 flow cytometer (BD Biosciences, NJ, USA). 2 μL FITC Mouse Anti-Human PAC-1 Antibody (BD Biosciences, NJ, USA) was used per 50 μL blood. Samples were diluted 1:200 before reading with Tyrode's Buffer (without calcium) and % PAC-1 binding was measured.

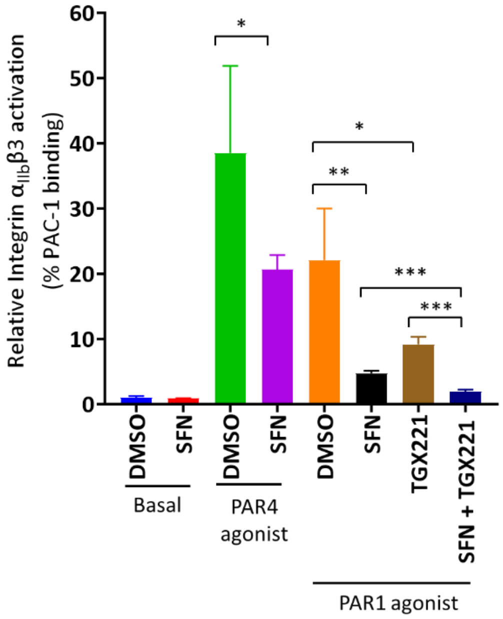

Figure S23. Flow cytometry analysis of platelet subpopulations exhibiting active integrin $\alpha_{IIb}\beta_3$ conformation (PAC-1 binding) under various treatment and stimulation conditions. Washed platelets were preincubated with DMSO or SFN as described previously, then incubated with DMSO or TGX-221, prior to stimulation with PAR-1 or PAR4-activating peptide for 10 minutes in the presence of FITC-PAC-1 (Mouse anti-human, clone PAC-1, BD). The experiment was conducted with three biological replicates for each treatment condition.

Section 6. In Vivo Biological Experiments

Section 6.1 Electrolytic injury

C57BL/6J male mice aged between 8-12 weeks, (22-30 grams) from Australian BioResources (ABR, NSW, Australia) were housed in the PC2 Rodent Holding Facility (Heart Research Institute) under a 12-hour light/dark cycle. Mice had *ad libitum* access to food and water. Studies were approved by the Sydney Local Health District Animal Welfare Committee (2021-027), in accordance with requirements of the Australian Code of Practice for the Care and Use of Animals for Scientific Purposes.

In brief, occlusion of the mouse left common carotid artery (CCA) was generated by electrolytic induction via an electrode composed of 2 platinum hooked arms (separated by 1 mm), manufactured by Ugo Basile (Comerio, VA, Italy). With the electrode held by a clamp, the mouse CCA was carefully placed atop the prongs of the electrode, positioned to produce stasis in blood flow distally. A clamp was additionally placed distally (Micro Serrefine Clamp, 18055-05; Fine Science Tools, North Vancouver, BC, Canada), and an 8 mA electrical current was delivered for 3 minutes via a lesion-making device (Model 53500; Ugo Basile). Post-injury, the clamp was removed, and the carotid was released from the electrode, resuming measurement of blood flow. The full procedure of electrolytic injury is described in depth in (1).

Mice treated prophylactically were injected with SFNp (50 mg/kg) via the femoral vein 1 hour prior to electrolytic injury. Other mice cohorts were treated with either rtPA alone (1 mg/kg bolus, 9 mg/kg infusion) or were co-treated with SFNp (50 mg/kg), 15 minutes post stable occlusion as determined using a 0.5 mm Transonic flow probe (0.5PSB, Transonic Systems Inc., Ithaca, New York, USA). rtPA was obtained from Boehringer Ingelheim Pty Ltd (Actilyse™, Alteplase; North Ryde, NSW, Australia) and dialyzed to remove arginine components as described (31). All experiments involving rtPA, were dosed at 1 mg/kg bolus over 30 seconds followed by 9 mg/kg bolus over 30 minutes.

Section 6.2 Tail Lop and Haemoglobin Assay

Mice were housed in the Laboratory Animal Services (LAS) Facilities (Charles Perkins Centre, University of Sydney) at similar conditions to above. Studies were approved by the University of Sydney Animal Ethics Committee (2021-1912).

Standard 3 mm tail lop procedures (32) were carried out with C57BL/6J mice male mice aged between 8-12 weeks, (22-30 g) from Australian BioResources (ABR, NSW, Australia), and bleeding was assessed. A hemoglobin assay (MAK115-1KT, Sigma Aldrich) was performed on blood collected from tail lop procedures according to the commercial protocol, and the number of bleeding events was counted. Statistical significance was calculated via an unpaired t-test.

Section 6.3 Generation of Platelet PDIA6 Knockout Mice

Mice were bred by crossing platelet factor 4 (Pf4) Cre^{+}/$PDIA6^{fl/fl}$ with $PDIA6^{fl/fl}$ mice to generate $Pf4Cre^{+}$ mice with PDIA6-deficient platelets and $Pf4Cre^{-}$ littermate controls. All mice were genotyped using tail clips as a source of genomic DNA to detect the Pf4Cre and floxed PDIA6 alleles [as we have described in reference 33]. Ten PDIA6 KO ($Pf4Cre^{+}$/$PDIA6^{fl/fl}$) along with the respective littermate controls ($PDIA6^{fl/fl}$) across both genders were generated for dedicated thrombolysis experiments (See **Figure S24**). These experiments were covered by University of Sydney Animal Ethics Approval Nr 2022/2040.

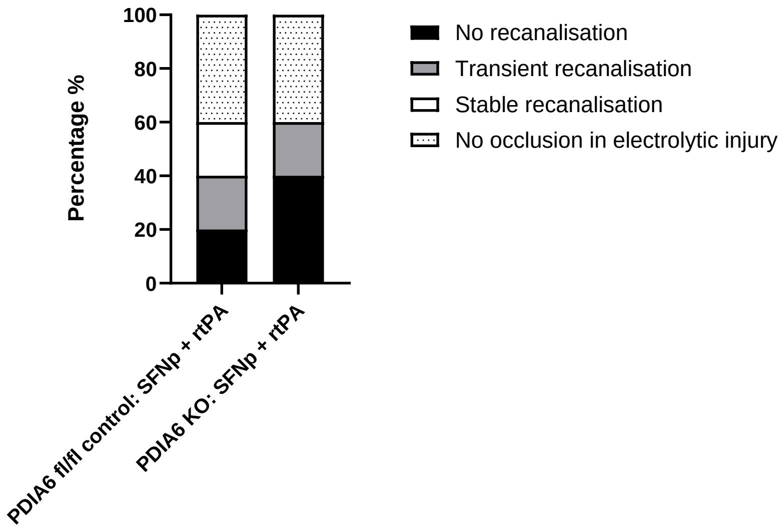

Figure S24. Recanalization outcomes of PDIA6 fl/fl (n=5) and PDIA6 KO (n=5) cohorts subject to SFN prophylactic treatment followed by rtPA-mediated clot lysis therapy. Note: mice falling into the category of “no occlusion in electrolytic injury” could not form a stable, occlusive clot and therefore were not delivered rtPA.

Section 7. Chemical Synthesis and Characterization

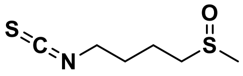

Sulforaphane, 1

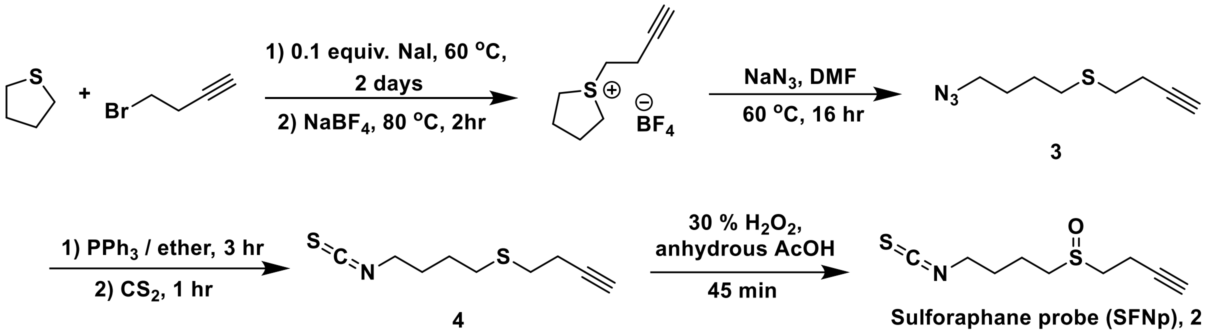

Scheme 1. Chemical structure of sulforaphane and the synthesis of the sulforaphane probe (SFNp).

But-3-yn-1-yl(4-azidobutyl)sulfane (**3**)

To the mixture of 4-bromo-1-butyne (1.5 g, 11.3 mmol, 1 equiv.) and tetrahydrothiophene (1.0 g, 11.3 mmol, 1 equiv.) was added sodium iodide (0.2 g, 1.1 mmol, 0.1 equiv.). The resulting mixture was stirred at 60 °C for 2 days under nitrogen atmosphere. The reaction mixture was diluted with n-butanol (10 mL), followed by the addition of $NaBF_4$ (1.2 g, 11.3 mmol, 1 equiv.) and stirring at 80 °C for 2 hours under nitrogen atmosphere. The resulting mixture was evaporated under a stream of nitrogen before dissolving in DMF (20 mL) followed by the addition of NaN_3 (2.2 g, 33.8 mmol, 3 equiv.). The resulting solution was stirred at 60 °C overnight under nitrogen atmosphere. The reaction was quenched with water (200 mL) and the aqueous fraction was extracted with Et_2O (3 × 20 mL). The organic fractions were combined, dried over Na_2SO_4, and concentrated under reduced pressure. The crude compound **3** (0.58 g) was obtained and used in the next step without further purification. ^{1}H NMR (300 MHz, $CDCl_3$): δ = 3.30 (t, *J* = 6.1 Hz, 2 H), 2.70 (t, *J* = 7.3 Hz, 2 H), 2.60 (t, *J* = 6.7 Hz, 2 H), 2.48 (td, *J* = 7.3 Hz, 2.6 Hz, 2 H), 2.03 (t, *J* = 2.6 Hz, 1 H), 1.70 (t, *J* = 3.3 Hz, 4 H); IR v_{max}/cm^{-1}: 3294, 2928, 2100, 1457, 1269.

But-3-yn-1-yl(4-isothiocyanatobutyl)sulfane (**4**)

To a solution of crude **3** (0.29 g) in Et_2O (10 mL) was added PPh_3 (0.42 g, 1.6 mmol, 1.0 equiv.). The resulting mixture was stirred at room temperature for 3 hours. Carbon disulfide (0.12 g, 1.6 mmol, 1.0 equiv.) was added to the reaction after solvent evaporation *in vacuo*. The reaction was stirred at room temperature overnight followed by the addition of 1M HCl (10 mL). The aqueous fraction was extracted with DCM (3 × 10 mL). The organic fractions were combined, dried over Na_2SO_4, and concentrated under reduced pressure. The crude oil was purified by flash chromatography (19:1 v/v, petroleum benzine:EtOAc) to provide compound **4** (110 mg, 35%) as light yellow oil. ^{1}H NMR (500 MHz, $CDCl_3$): δ = 3.55 (t, *J* = 6.3 Hz, 2 H), 2.70 (t, *J* = 7.3 Hz, 2 H), 2.62 (t, *J* = 7.0 Hz, 2 H), 2.49 (td, *J* = 7.3 Hz, 2.6 Hz, 2 H), 2.04 (t, *J* = 2.6 Hz, 1 H), 1.79 – 1.85 (quint, *J* = 6.7 Hz, 2 H), 1.71 – 1.77 (quint, *J* = 7.2 Hz, 2 H); ^{13}C NMR (125 MHz, $CDCl_3$): δ = 81.5, 68.5, 43.7, 30.3, 29.9, 27.9, 25.4, 18.9; IR ν_{max}/cm^{-1}: 3289, 2925, 2181, 2097, 1448, 1347. HRMS (ESI+) m/z calcd. for $C_9H_{13}NS_2$ $[M+Na]^+$ 222.0382, found 222.0382.

4-((4-Isothiocyanatobutyl)sulfinyl)but-1-yne (**2**)

To a solution of **4** (0.11 g, 0.55 mmol, 1.0 equiv.) in anhydrous acetic acid (0.5 mL) was added 30% hydrogen peroxide (2.2 mmol, 4.0 equiv.). The resulting mixture was stirred at room temperature for 45 minutes and reaction quenched with 1M NaOH (1 mL). The aqueous fraction was extracted with DCM (5 × 1 mL). The organic fractions were combined, dried over Na_2SO_4, and concentrated under reduced pressure to provide **2** (110 mg, 92%) as a white solid. ^{1}H NMR (500 MHz, $CDCl_3$): δ = 3.60 (t, *J* = 6.2 Hz, 2 H), 2.88 (t, *J* = 7.0 Hz, 2 H), 2.71 – 2.84 (m, 4 H), 2.08 (t, *J* = 2.5 Hz, 1 H), 1.84 – 2.00 (m, 4 H); ^{13}C NMR (125 MHz, $CDCl_3$): δ = 80.7, 70.7, 51.4, 50.8, 44.7, 29.1, 20.3, 12.5; IR ν_{max}/cm^{-1}: 3289, 3224, 2925, 2185, 2107, 1437, 1350, 1026; HRMS (ESI+) m/z calcd. for $C_9H_{13}NOS_2$ $[M+Na]^+$ 238.0331, found 238.0332.

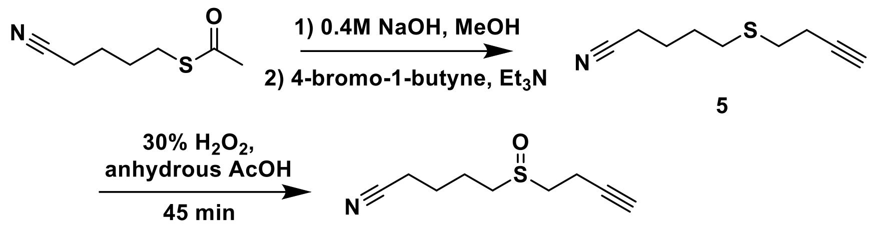

Scheme 2. Chemical synthesis of the negative control probe (NCOp).

5-(But-3-yn-1-ylthio)pentanenitrile (**5**)

To a solution of S-(4-cyanobutyl)thioacetate (0.50 g, 3.2 mmol, 1.0 equiv.) in MeOH (2 mL) was added NaOH to achieve a concentration of 0.4 M NaOH. The resulting mixture was stirred at room temperature for 1.5 hours. 4-Bromo-1-butyne (0.43 g, 6.4 mmol, 2.0 equiv.) and Et_3N (1.61 g, 15.9 mmol, 5.0 equiv.) were subsequently added to the reaction. The resulting mixture was stirred under reflux overnight. The reaction was quenched with 1M HCl (10 mL) followed by extraction with EtOAc (3 × 10 mL). The organic fractions were combined, dried over Na_2SO_4, and concentrated under reduced pressure. The crude oil was purified by flash chromatography (9:1 v/v, petroleum benzine:EtOAc) to provide compound **5** (330 mg, 61%) as a colorless oil. ^{1}H NMR (500 MHz, $CDCl_3$): δ = 2.70 (t, *J* = 7.3 Hz, 2 H), 2.62 (t, *J* = 6.4 Hz, 2 H), 2.49 (td, *J* = 7.3 Hz, 2.4 Hz, 2 H), 2.38 (t, *J* = 6.4 Hz, 2 H), 2.04 (t, *J* = 2.3 Hz, 1 H), 1.78 (t, *J* = 3.3 Hz, 4 H); 13C NMR (125 MHz, $CDCl_3$): δ = 119.4, 82.6, 69.7, 31.3, 31.0, 28.4, 24.4, 20.0, 17.0; IR vmax/cm^{-1}: 3286, 2930, 2240, 1422; HRMS (ESI+) m/z calc. for $C_9H_{13}NS$ $[M+Na]^+$ 190.0661, found 190.0662.

5-(But-3-yn-1-ylsulfinyl)pentanenitrile (**6**)

To a solution of compound **5** (0.24 g, 1.4 mmol, 1.0 equiv.) in anhydrous acetic acid (1 mL) was added 30% hydrogen peroxide (5.7 mmol, 4.0 equiv.). The resulting mixture was stirred at room temperature for 6 hours and subsequently quenched with 1M NaOH (2 mL). The

aqueous fraction was extracted with EtOAc (3 × 2 mL). The organic fractions were combined, dried over Na_2SO_4, and concentrated under reduced pressure. The crude solid was purified by flash chromatography (39:1 v/v, DCM:MeOH) to provide compound **6** (230 mg, 86%) as a white solid. ^{1}H NMR (500 MHz, $CDCl_3$): δ = 2.86 (t, *J* = 7.1 Hz, 2 H), 2.69 – 2.82 (m, 4 H), 2.42 (td, *J* = 7.0 Hz, 2 H), 2.07 (t, *J* = 2.6 Hz, 1 H), 1.99 (quint, *J* = 7.6 Hz, 2 H), 1.81 – 1.93 (m, 2 H); ^{13}C NMR (125 MHz, $CDCl_3$): δ = 119.1, 80.8, 70.8, 51.4, 51.0, 24.7, 22.2, 17.1, 12.8; IR vmax/cm^{-1}: 3438, 3286, 2934, 2247, 1426, 1017; HRMS (ESI+) m/z calc. for $C_9H_{13}NOS$ $[M+Na]^+$ 206.0610, found 206.0611.

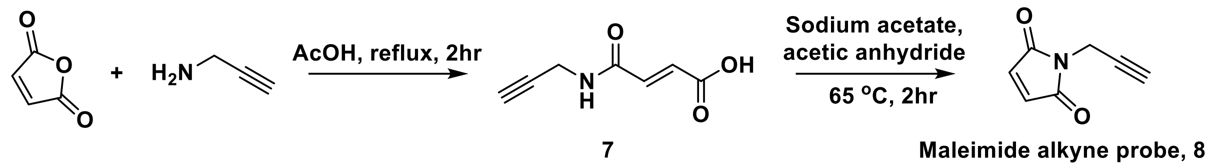

Scheme 3. Chemical synthesis of the maleimide alkyne probe.

4-Oxo-4-(prop-2-yn-1-ylamino)but-2-enoic acid (**7**)

To a solution of maleic anhydride (0.89 g, 9.1 mmol, 1.0 equiv.) in acetic acid (10 mL) was added propargylamine (0.5 g, 9.1 mmol, 1.0 equiv.). The resulting mixture was stirred under reflux for 2 hours. The reaction mixture was then concentrated under reduced pressure with toluene as the azeotropic solvent (3 × 20 mL). The crude compound **7** was obtained and used in the next step without further purification. ^{1}H NMR (300 MHz, CD_3OD): δ = 6.86 (s, 1 H), 6.43 (d, *J* = 12.6 Hz, 1 H), 6.26 (d, *J* = 12.6 Hz, 1 H), 4.08 (d, *J* = 2.4 Hz, 2 H), 2.67 (t, *J* = 2.5 Hz, 1 H).

N-Propargylmaleimide (**8**)

To a solution of compound **7** in acetic anhydride (8 mL) was added sodium acetate (0.74 g, 9.1 mmol, 1.0 equiv.). The resulting mixture was stirred at 65 °C for 2 hours. The reaction was allowed to cool to room temperature and poured into ice-cold water (80 mL). The aqueous

fraction was extracted with Et_2O (3 × 80 mL). The organic fractions were combined, dried over Na_2SO_4, and concentrated under reduced pressure. The crude solid was purified by flash chromatography (1:1 v/v, petroleum benzine:EtOAc) to provide compound **8** (0.76 g, 62%) as a pale yellow solid. ^1H NMR (300 MHz, $CDCl_3$): δ = 6.75 (s, 2 H), 4.29 (d, *J* = 2.5 Hz, 2 H), 2.21 (t, *J* = 2.5 Hz, 1 H). ^1H NMR spectrum matches the reported literature (33).

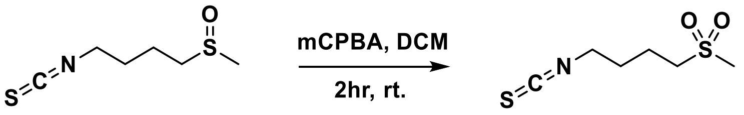

1 9

Scheme 4. Chemical synthesis of erysolin.

Erysolin (9)

To a solution of sulforaphane (0.02 g, 0.11 mmol, 1 equiv.) in anhydrous DCM (0.5 mL) was added m-chloroperoxybenzoic acid (0.23 mmol, 2 equiv.). The resulting mixture was stirred at room temperature for 2 hours under argon. The reaction was diluted with DCM (2 mL) and the organic fraction was washed with sat. $NaHCO_3$ (3 mL). The organic fractions were combined, dried over Na_2SO_4, and concentrated under reduced pressure. The crude product was purified by flash chromatography (DCM) to provide compound **9** (quant.) as a white solid. ^1H NMR (300 MHz, $CDCl_3$): δ = 3.62 (t, *J* = 6.2 Hz, 2 H), 3.07 (t, *J* = 7.5 Hz, 2 H), 2.95 (s, 3 H), 1.98 – 2.08 (m, 2 H), 1.86 – 1.95 (m, 2 H). ^1H NMR spectrum matches the reported literature (34).

References

1. Ku S, Lee I, Kim J, Bae J. Antithrombotic activities of pellitorine in vitro and in vivo. Fitoterapia. 2013;91:1-8.

2. Son D, Akiba S, Hong J, Yun Y, Hwang S, Park Y, Lee S. Piperine Inhibits the Activities of Platelet Cytosolic Phospholipase A2 and Thromboxane A2 Synthase without Affecting Cyclooxygenase-1 Activity: Different Mechanisms of Action Are Involved in the Inhibition of Platelet Aggregation and Macrophage Inflammat. Nutrients. 2014;6(8):3336-52.

3. Wang R, Zhou S, Zhang F, Mei Q. Study of pharmacological effects and mechanism of ethyl ferulate. Chinese J Pharmacol Toxicol. 2004;9(8):925.

4. Kim SD, Lee YJ, Baik JS, Han JY, Lee CG, Heo K, Park YS, Kim JS, Ji HD, Park SI, et al. Baicalein inhibits agonist- and tumor cell-induced platelet aggregation while suppressing pulmonary tumor metastasis via cAMP-mediated VASP phosphorylation along with impaired MAPKs and PI3K-Akt activation. Biochem Pharmacol. 2014;92(2):251-65.

5. Gaspar RS, da Silva SA, Stapleton J, Fontelles JLdL, Sousa HR, Chagas VT, Alsufyani S, Trostchansky A, Gibbins JM, AMdA P. Myricetin, the Main Flavonoid in Syzygium cumini Leaf, Is a Novel Inhibitor of Platelet Thiol Isomerases PDI and ERp5. Front Pharmacol. 2020;10(1678).

6. Seo EJ, Lee DU, Kwak JH, Lee SM, Kim YS, Jung YS. Antiplatelet effects of Cyperus rotundus and its component (+)-nootkatone. J Ethnopharmacol. 2011;135(1):48-54.

7. Mester L, Mester M, Nityanand S. Inhibition of Platelet Aggregation by „Guggulu" Steroids. Planta Med. 1979;37(12):367-9.

8. Amirou A, Razzok EM, Legssyer A, Ziyyat A, Aziz M, Bnouham M, Zaid Y, Berrabah M, Mekhfi H. Effects of Walnut Bark Extract on the Human Platelet Aggregation, Adhesion, and Plasmatic Coagulation In Vitro. Adv Pharmacol Pharm Sci. 2023;2023:1-9.

9. Ku S, Bae J. Antiplatelet, anticoagulant, and profibrinolytic activities of withaferin A. Vascul Pharmacol. 2014;60(3):120-6.

10. Lee YM, Hsieh KH, Lu WJ, Chou HC, Chou DS, Lien LM, Sheu JR, KH L. Xanthohumol, a Prenylated Flavonoid from Hops (Humulus lupulus), Prevents Platelet Activation in Human Platelets. Evid Based Complement Alternat Med. 2012;2012:1-10.

11. Chuang WY, Kung PH, Kuo CY, Wu CC. Sulforaphane prevents human platelet aggregation through inhibiting the phosphatidylinositol 3-kinase/Akt pathway. J Thromb Haemost. 2013;109(06):1120-30.

12. Fuentes E, Alarcón M, Fuentes M, Carrasco G, Palomo I. A Novel Role of Eruca sativa Mill. (Rocket) Extract: Antiplatelet (NF-κB Inhibition) and Antithrombotic Activities. Nutrients. 2014;6(12):5839-52.

13. Lee D-S, Kim T-H, Jung Y-S. Inhibitory Effect of Allyl Isothiocyanate on Platelet Aggregation. J Agric Food Chem. 2014;62(29):7131-9.

14. Jumper J, Evans R, Pritzel A, Green T, Figurnov M, Ronneberger O, Tunyasuvunakool K, Bates R, Žídek A, Potapenko A, et al. Highly accurate protein structure prediction with AlphaFold. Nature. 2021;596(7873):583-9.

15. Consortium TU. UniProt: the Universal Protein Knowledgebase in 2023. Nucleic Acids Res. 2022;51(D1):D523-D31.

16. Madhavi SG, Adzhigirey M, Day T, Annabhimoju R, Woody S. Protein and ligand preparation: parameters, protocols, and influence on virtual screening enrichments. J Comput Aided Mol Des. 2013;27(3):221-34.

17. Bowers KJ, Chow DE, Xu H, Dror RO, Eastwood MP, Gregersen BA, Klepeis JL, Kolossvary I, MA M, Sacerdoti FD, et al., editors. Scalable Algorithms for Molecular Dynamics Simulations on Commodity Clusters. ACM/IEEE Conference on Supercomputing; 2006: ACM Press.

18. Halgren TA. Identifying and Characterizing Binding Sites and Assessing Druggability. J Chem Inf Model. 2009;49(2):377-89.

19. Halgren T. New Method for Fast and Accurate Binding-site Identification and Analysis. Chem Biol Drug Des. 2007;69(2):146-8.

20. Friesner RA, Murphy RB, Repasky MP, Frye LL, Greenwood JR, Halgren TA, Sanschagrin PC, DT M. Extra Precision Glide: Docking and Scoring Incorporating a Model of Hydrophobic Enclosure for Protein−Ligand Complexes. J Med Chem. 2006;49(21):6177-96.

21. Halgren TA, Murphy RB, Friesner RA, Beard HS, Frye LL, Pollard WT, JL B. Glide: A New Approach for Rapid, Accurate Docking and Scoring. 2. Enrichment Factors in Database Screening. J Med Chem. 2004;47(7):1750-9.

22. Friesner RA, Banks JL, Murphy RB, Halgren TA, Klicic JJ, Mainz DT, Repasky MP, Knoll EH, Shelley M, Perry JK, et al. Glide: A New Approach for Rapid, Accurate Docking and Scoring. 1. Method and Assessment of Docking Accuracy. J Med Chem. 2004;47(7):1739-49.

23. Farid R, Day T, Friesner RA, Pearlstein RA. New insights about HERG blockade obtained from protein modeling, potential energy mapping, and docking studies. Bioorg Med Chem. 2006;14(9):3160-73.

24. Sherman W, Day T, Jacobson MP, Friesner RA, Farid R. Novel Procedure for Modeling Ligand/Receptor Induced Fit Effects. J Med Chem. 2006;49(2):534-53.

25. Sherman W, Beard HS, Farid R. Use of an Induced Fit Receptor Structure in Virtual Screening. Chem Biol Drug Des. 2006;67(1):83-4.

26. Jacobson MP, Friesner RA, Xiang Z, Honig B. On the Role of the Crystal Environment in Determining Protein Side-chain Conformations. J Mol Biol. 2002;320(3):597-608.

27. Zhu K, Borrelli KW, Greenwood JR, Day T, Abel R, Farid RS, E H. Docking Covalent Inhibitors: A Parameter Free Approach To Pose Prediction and Scoring. J Chem Inf Model. 2014;54(7):1932-40.

28. Chen C, Chen H, Zhang Y, Thomas HR, Frank MH, He Y, R X. TBtools: An Integrative Toolkit Developed for Interactive Analyses of Big Biological Data. Mol Plant. 2020;13(8):1194-202.

29. Liu X, Li X, Chen L, Hsu AC-Y, Asquith KL, Liu C, Laurie K, Barr I, Foster PS, Yang M. Proteomic Analysis Reveals a Novel Therapeutic Strategy Using Fludarabine for Steroid-Resistant Asthma Exacerbation. Front Immunol. 2022;13.

30. Li X, Liu H, Dun MD, Faulkner S, Liu X, Jiang CC, H H. Proteome and secretome analysis of pancreatic cancer cells. Proteomics. 2022;22(13-14):2100320.

31. Maclean JAA, Tomkins AJ, Sturgeon SA, Hofma BR, Alwis I, Samson AL, Schoenwaelder SM, SP J. Development of a carotid artery thrombolysis stroke model in mice. Blood Adv. 2022;6(18):5449-62.

32. Schoenwaelder SM, Jarman KE, Gardiner EE, Hua M, Qiao J, White MJ, Josefsson EC, Alwis I, Ono A, Willcox A, et al. Bcl-xL–inhibitory BH3 mimetics can induce a transient thrombocytopathy that undermines the hemostatic function of platelets. Blood. 2011;118(6):1663-74.

33. Xu L, Li N, Zhang B, Chen J, Kang E. PEGylated Fluorescent Nanoparticles from One-Pot Atom Transfer Radical Polymerization and "Click Chemistry". Polymers. 2015;7(10):2119-30.

34. Mays JR, Weller Roska RL, Sarfaraz S, Mukhtar H, Rajski SR. Identification, Synthesis, and Enzymology of Non-natural Glucosinolate Chemopreventive Candidates. ChemBioChem. 2008;9(5):729-47.
